# UBE4B promotes gastric cancer proliferation and metastasis by mediating FAT4 ubiquitination and degradation

**DOI:** 10.1038/s41419-025-07794-8

**Published:** 2025-07-23

**Authors:** Kaini Wu, Zixiang Guo, Yunfeng Fu, Sicheng Yang, Yating Pan, Runwei Yan, Xiaodong Zhou

**Affiliations:** Department of Gastroenterology, Jiangxi Provincial Key Laboratory of Digestive Diseases, Jiangxi Clinical Research Center for Gastroenterology, Digestive Disease Hospital, The First Affiliated Hospital, Jiangxi Medical College, Nanchang University, Nanchang, Jiangxi China

**Keywords:** Tumour biomarkers, Gastrointestinal cancer

## Abstract

The ubiquitin‒proteasome system (UPS), an intracellular protein degradation pathway, plays an important role in regulating tumorigenesis and development. Ubiquitination factor E4B (UBE4B/UFD2) has been shown to be associated with the development of several cancers. The aim of this study was to reveal the functional significance of UBE4B in gastric cancer (GC) development and its important mechanism. Bioinformatics analysis, immunohistochemistry (IHC), western blotting, and real-time PCR were performed to detect UBE4B expression in human GC samples and GC cell lines and a mouse xenograft tumour model was established. Our investigation revealed that UBE4B is highly expressed in GC and promotes the proliferation, migration and invasion of GC cells. The quantitative Tandem Mass Tag (TMT) analysis revealed that FAT oncogenic homologue 4 (FAT4) is a downstream gene of UBE4B. Western blot experiments and transmission electron microscopy (TEM) results for biological samples revealed that UBE4B inhibits autophagy in GC cells and directly binds to and degrades FAT4 through ubiquitination. These results suggest that UBE4B can inhibit autophagy and promote GC progression by mediating FAT4 ubiquitination and degradation, and our findings provide a new potential therapeutic target for GC management.

## Introduction

Gastric cancer (GC) is the fifth most common malignant tumour worldwide and the fifth most common cause of cancer-related death [1]. Although the incidence of GC is declining in most countries, clinical experts predict an increase in the number of GC cases in the future as the population continues to age [2]. As the pathogenesis of GC is still unclear, new and more promising targets must be identified for future GC treatments to improve the prognosis of GC patients.

Protein homeostasis is the basis for normal cell function and survival, and the regulation of protein degradation is an important strategy to maintain protein homeostasis [3]. Among these pathways, the ubiquitin‒proteasome system (UPS) is an ATP dependent, highly specific mode of intracellular protein degradation that plays key roles in cell differentiation, cell proliferation, DNA damage, and repair [4]. The UPS system includes ubiquitin, the ubiquitin-activating enzyme E1, the ubiquitin-conjugating enzyme E2, the ubiquitin ligase E3, and the 26S proteasome [5]. Dysregulated expression of E3 ubiquitin ligases, a central component of the ubiquitination step, leads to the accumulation of misfolded or dysfunctional proteins, which promotes cancer development and progression [6]. In recent years, enzymes targeting UPS, including ubiquitin ligases and deubiquitinating enzymes, have emerged as a cutting-edge area of cancer therapy. By regulating the stability, localization, or function of key proteins, they show great potential in overcoming tumor drug resistance and selectively killing cancer cells [7–9].

Ubiquitination factor E4B (UBE4B/UFD2) is the mammalian homologue of *Saccharomyces cerevisiae* UFD2. It is located on chromosome 1p and contains a conserved U-box catalytic structural domain of approximately 70 amino acids [10]. As an important E3/E4 ubiquitin ligase, UBE4B is involved in biological processes such as embryonic development and neuronal protection and is associated with the development of various diseases [11]. Indeed, functional abnormalities of UBE4B have been confirmed in several studies as a key regulatory factor of neurodegenerative diseases such as Alzheimer’s disease (AD), Machado-Joseph disease (MJD), and peroneal muscular dystrophy (CMT) [12–14]. The role of UBE4B in tumors is dual, acting as both a tumor suppressor and a tumor promoter, depending on the tumor type and cellular environment. In lung cancer, the potential of targeting the UBE4B-HuR-p27 axis as a therapeutic strategy was identified [15]. In oesophageal cancer, circUBE4B, encoding circUBE4B-173aa, interacts with the MAPK1 protein and activates the MAPK/ERK pathway, thereby promoting tumour cell proliferation [16]. In breast cancer, UBE4B further promotes tumorigenesis by promoting the ubiquitination and degradation of p53, thereby inhibiting apoptosis in cancer cells [17]. In contrast, findings have shown the aberrant downregulation of UBE4B gene expression in neuroblastoma and oral squamous cell carcinoma [18–20]. However, no study has investigated the expression of UBE4B in GC and its role in gastric carcinogenesis.

FAT atypical calcineurin 4 (FAT4) is the closest vertebrate homologue of Drosophila fat and is located on chromosome 4q28.1 [21]. It was originally discovered in a mouse mammary epithelial cell line and belongs to the family of FAT proteins (FAT1–4) found in mammals. FAT4 knockout mice die at birth and exhibit PCP defects in tissues such as the kidney, inner ear, neural tube, and cerebral cortex [22]. FAT4 is not only involved in regulating cell growth and planar cell polarity but has also been implicated as a tumour suppressor in a variety of tumours [23]. Several studies have shown that FAT4 has cancer-inhibiting effects on tumours such as breast cancer [24], cervical cancer [25], and GC [26–28]. More importantly, FAT4 promotes autophagy and inhibits the invasion, migration, and proliferation of lung and colorectal cancer cells [29, 30]. Regarding the mechanism by which FAT4 inhibits gastric carcinogenesis and progression, several aspects, including Yap nuclear translocation [26], the regulation of Wnt/β-catenin signalling [27], and the activation of the PI3K-AKT signalling pathway by miR-107, which targets FAT4 [28], have been reported in recent studies. Furthermore, most previous studies have focused on the regulation of downstream signalling molecules by FAT4 or the effects of other upstream regulators on FAT4 but not on the degradation of FAT4 via posttranslational modifications.

Therefore, the aim of this study was to investigate the expression of UBE4B in GC and its mechanism of action in the development of gastric cancer. We performed a bioinformatics analysis and immunohistochemistry (IHC) to detect the expression of UBE4B in human GC samples and found that UBE4B was highly expressed in GC and was closely associated with a poor prognosis for patients. Cellular functional assays revealed that the overexpression of UBE4B promoted the proliferation, migration, and invasion of GC cells. We performed a quantitative TMT analysis to further explore the role of UBE4B and its downstream mechanisms. In this study, we identified FAT4 as a key target gene of UBE4B. Moreover, we performed western blotting and Co-IP, and established a mouse xenograft tumour model to determine the relationship between these two molecules and the specific mechanism of action. Our results found that UBE4B could inhibit autophagy and promote GC progression by mediating the ubiquitination and degradation of FAT4. Finally, we similarly validated these findings in a large sample of human subjects. We investigated for the first time the role of UBE4B in GC and its molecular mechanism, providing new potential therapeutic targets for GC management.

### Statistical analysis

SPSS 26.0 software (SPSS, IL, USA) and GraphPad Prism software (GraphPad, CA, USA) were used for the statistical analyses of the experimental data. Unless otherwise indicated, the data were expressed as the mean ± SD from experiments conducted a minimum of three times. Comparisons of data that conformed to a normal distribution between two groups were performed using Student’s t test, while data that did not conform to a normal distribution were analysed using the rank-sum test, and comparisons between multiple groups of data were performed using one-way analysis of variance (ANOVA). Survival curves were generated via the Kaplan–Meier (K–M) method and compared using the log-rank test. *P* < 0.05 (***p < 0.001, **p < 0.01, *p < 0.05) was considered statistically significant.

## Results

### The expression of UBE4B is correlated with the progression of GC

**Fig. 1:**
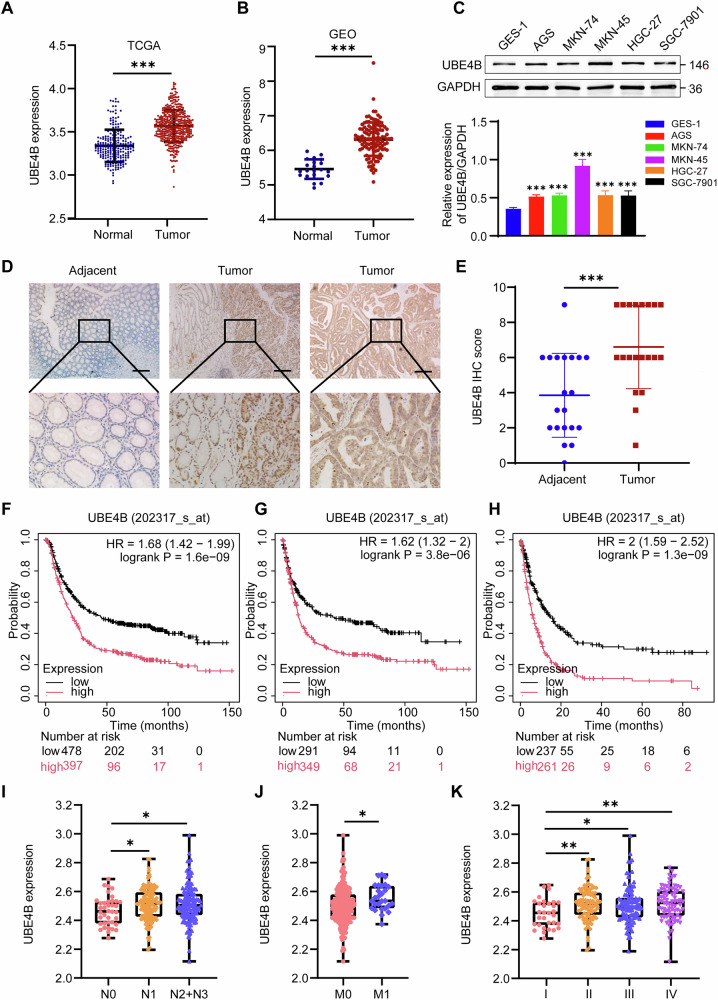
UBE4B was elevated in gastric cancer. **A** The real-time PCR assay of the mRNA expression of UBE4B in gastric cancer (GC) tissues and normal tissues from the TCGA database. **B** The real-time PCR assay of the mRNA expression of UBE4B in GC tissues and normal tissues from the GEO database. **C** The basic UBE4B protein level in different GC cell types. **D** Immunohistochemistry (IHC) staining for UBE4B in GC and adjacent normal tissue. Bar length: 50 μm. **E** IHC score analyzed the expression levels of UBE4B in GC and adjacent normal tissue. **F**–**H** The Kaplan–Meier plot of Overall survival (OS), progression-free survival (FP), and post-progression survival (PPS) by the expression of UBE4B in the GC patients, the data was carried out from the TCGA database. **I**–**K** Clinicopathologic features of UBE4B expression and N-stage, M-stage, and stage in GC patients, the data was carried out from the GEO database.

We downloaded and analysed the mRNA expression data for GC patients from the Gene Expression Omnibus (GEO) and The Cancer Genome Atlas (TCGA) databases to explore UBE4B expression in gastric cancer tissues. The results revealed that the expression level of UBE4B was greater in tumour tissues than in adjacent normal tissues (Fig. 1A, B). Moreover, we measured the expression levels in different GC cell lines and found higher UBE4B protein levels in GC cells than in GES-1 cells (Fig. 1C). Immunohistochemical (IHC) staining revealed that the expression level of UBE4B in GC tissues was significantly higher than that in normal tissues (*p* < 0.001), and the lower the degree of differentiation was, the higher the expression of UBE4B in GC tissues (Fig. 1D, E).

The correlation between UBE4B expression and the prognosis of GC patients was analysed using TCGA data and the online analysis software Kaplan‒Meier Plotter. Overall survival (Fig. 1F), progression-free survival (Fig. 1G), and postprogression survival (Fig. 1H) were significantly lower in GC patients with high UBE4B expression than in those with low UBE4B expression (*p* < 0.001). We screened and analysed the gene expression data and clinical information from the GEO database to understand the correlation between UBE4B expression and the clinicopathological features of GC patients. The results revealed that UBE4B expression was correlated with the age, N stage, M stage, and stage of GC patients (*p* < 0.05). The expression level of UBE4B in gastric cancer patients <60 years old was higher than that in patients ≥60 years old (Supplementary Fig. 1A). UBE4B expression was higher in N1/N2 stage GC tissues than in N0 stage GC tissues (Fig. 1I). UBE4B was expressed at higher levels in GC tissues that developed metastasis than in those that did not (Fig. 1J). UBE4B expression was significantly higher in stage II/III/IV GC tissues than in stage I GC tissues (Fig. 1K). However, no difference in UBE4B expression was observed in GC tissues with different T stages or between different types of GC (Supplementary Fig. 1B, C).

### UBE4B promotes the proliferation, migration, and invasion of GC cells

**Fig. 2:**
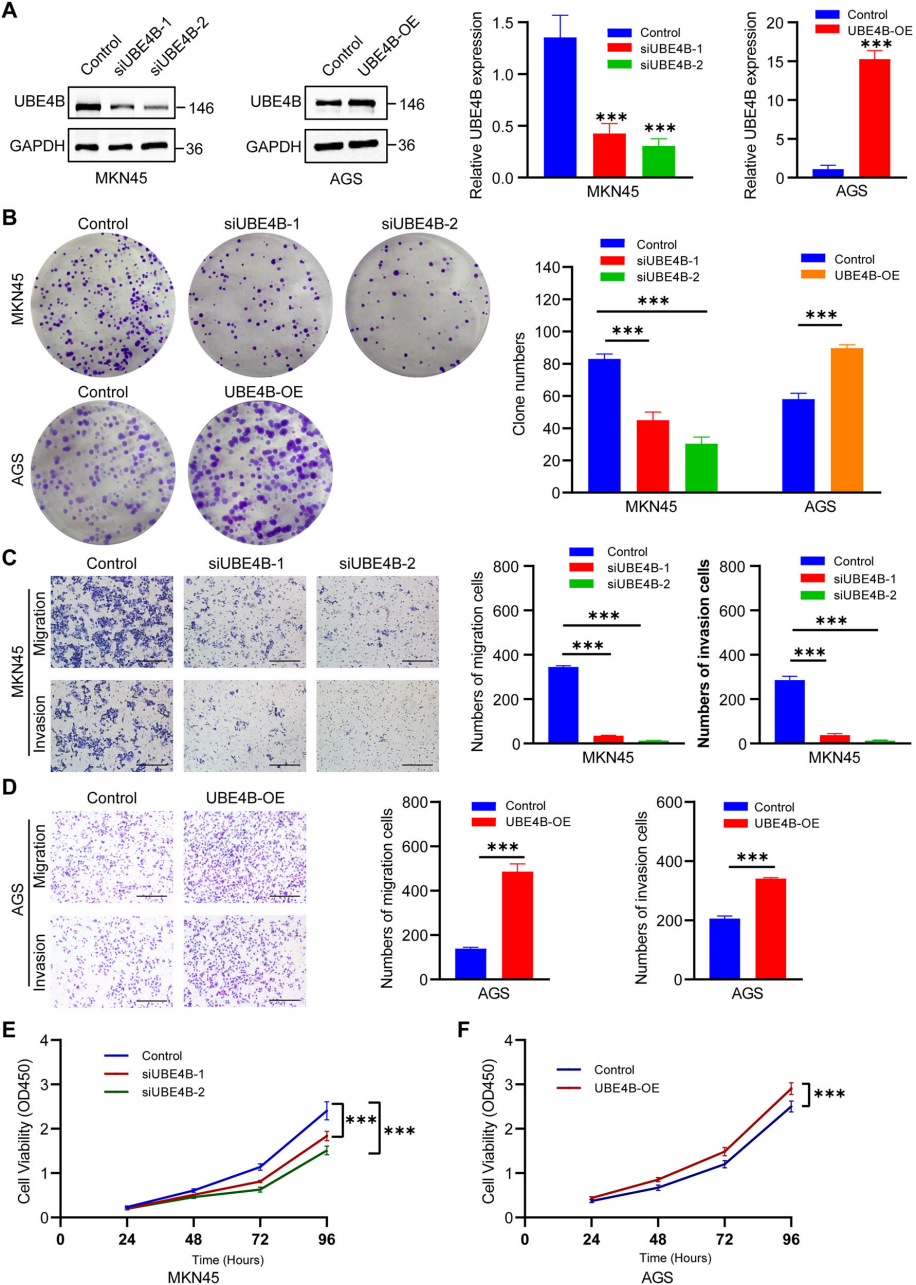
UBE4B promoted the proliferation, migration, and invasion of GC cell lines. **A** Western blot analysis of UBE4B protein levels and Real-time PCR analysis of UBE4B mRNA levels in knockdown cell line (MKN45) and overexpression cell line (AGS). **B** Colony formation assay was used to detect the proliferation of knockdown UBE4B and control GC cells, and overexpressing UBE4B and control GC cells. **C** Invasion and migration assay were performed to detect the proliferation of knockdown UBE4B and control MKN45 cells. Bar length: 100 μm. **D** Invasion and migration assays were performed to detect the proliferation of overexpression UBE4B and control AGS cells. Bar length: 100 μm. **E** The CCK-8 assay was performed to detect the proliferation of knockdown UBE4B and control MKN45 cells. **F** The CCK-8 assay was performed to detect the proliferation of overexpression UBE4B and control AGS cells.

We utilized siUBE4B and overexpression plasmids to knockdown and overexpress UBE4B in MKN45 and AGS cells to investigate the potential role of UBE4B in the development of GC. Real-time PCR analysis of UBE4B mRNA levels and Western blot analysis of UBE4B protein levels confirmed the successful construction of the siUBE4B and overexpression plasmids (Fig. 2A). The results of the colony formation assays indicated that a significantly reduced number of colonies formed from UBE4B-knockdown GC cells compared to control cells. The number of colonies formed by UBE4B-overexpressing GC cells was significantly increased (Fig. 2B). A transwell migration assay was performed to assess the effect of UBE4B on cell migration. Knockdown of UBE4B significantly decreased the migration of GC cells, whereas overexpression of UBE4B significantly promoted the migration of GC cells (Fig. 2C, D). Further Transwell assays to assess the invasive ability of GC cells revealed that when UBE4B was downregulated, GC cell invasion was inhibited, and its upregulation promoted invasion (Fig. 2C, D). We performed a Cell Counting Kit-8 (CCK-8) assay to assess the effect of UBE4B on cell proliferation. The results of the CCK-8 assay revealed that UBE4B knockdown decreased GC cell viability and its overexpression increased GC cell viability (Fig. 2E, F).

### UBE4B interacts with and ubiquitinates FAT4

**Fig. 3:**
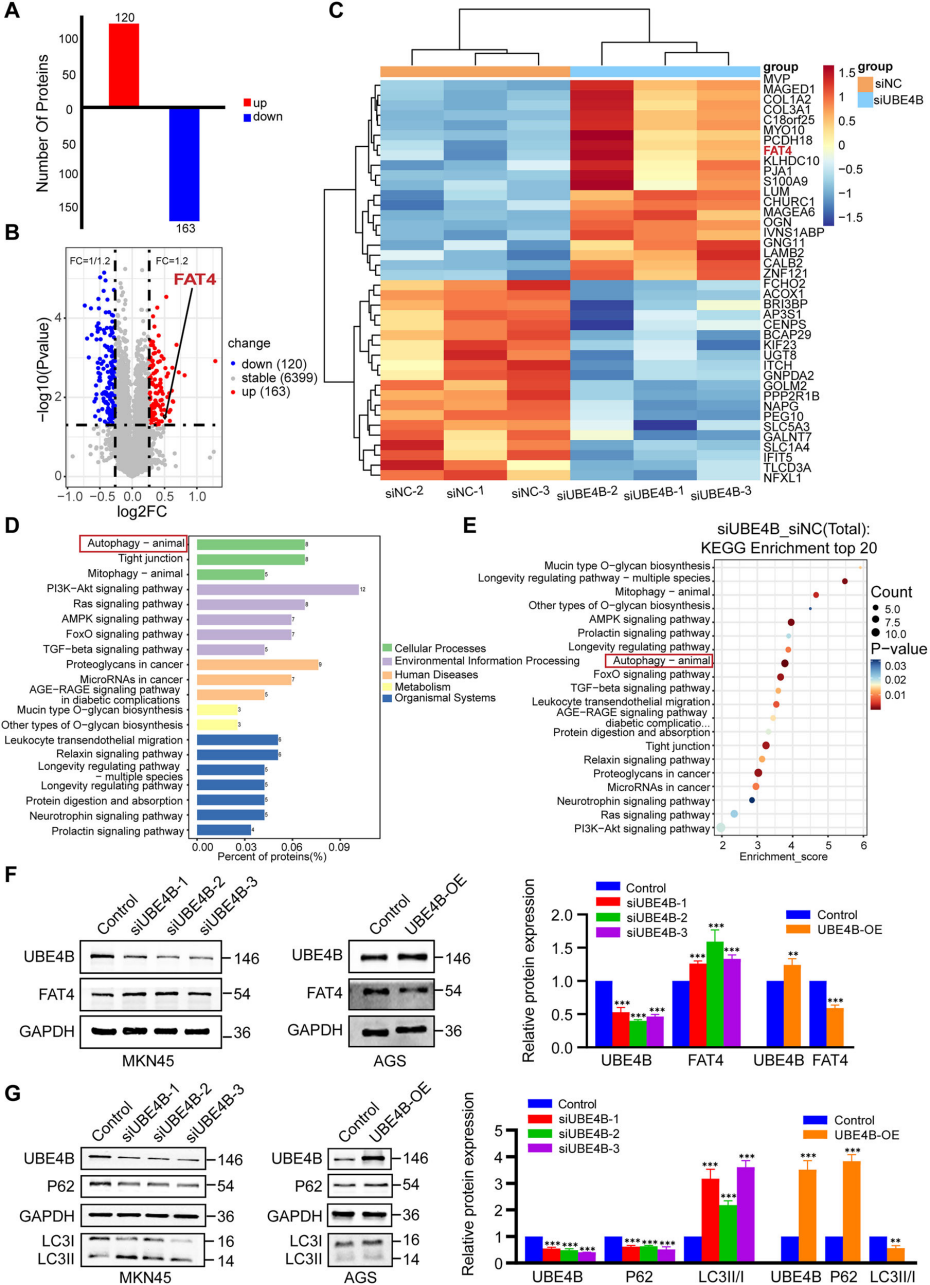
UBE4B mediates the degradation of FAT4. **A** Mass spectroscopic analysis of Up-regulated and down-regulated protein. **B** Volcano plot showing differentially expressed proteins (*P* < 0.05, FC < 1/1.2 & FC > 1.2). **C** Heatmap shows a differentially expressed gene. **D** KEGG enrichment analysis top 20. **E** KEGG Level 3 shows differential protein distribution. **F** Western blot detected FAT4 protein in MKN45 or AGS cells after transfecting cells with three independent siRNA or UBE4B overexpression plasmid. **G** Western blot detected P62 and LC3II/I protein in MKN45 or AGS cells after transfecting cells with three independent siRNA or UBE4B overexpression plasmid.

**Fig. 4:**
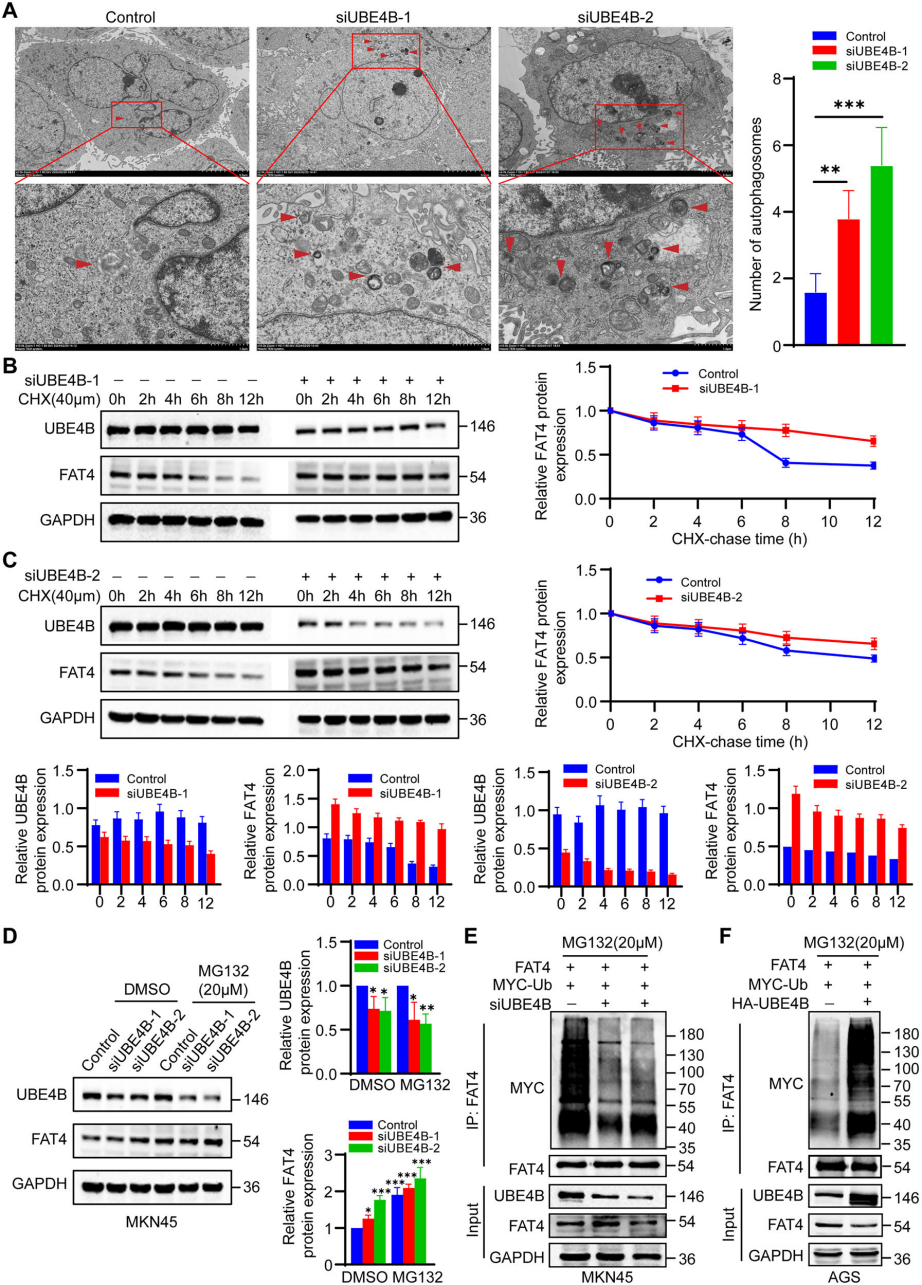
UBE4B mediates the degradation of FAT4 through ubiquitination. **A** Transmission electron microscopy observation of autophagosomes in UBE4B-knockdown MKN45 cells. **B, C** MKN45 cells transfected with two independent siUBE4B were treated with 20 μM cycloheximide (CHX) for 0, 2, 4, 6, 8, and 12 h, and the UBE4B and FAT4 protein levels were detected by Western blot. **D** Western blotting was conducted to measure FAT4 protein level after treatment with 20 μM MG132 in knockdown UBE4B and control GC cells. **E, F** Western blotting was conducted to measure the level of ubiquitin when knockdown or overexpression UBE4B.

We performed TMT (tandem mass tag)-labelled quantitative proteome analysis to detect changes in the protein expression profiles of UBE4B-knockdown GC cells compared with controls. The analysis revealed that a total of 120 upregulated proteins and 163 downregulated proteins were significantly differentially expressed (Fig. 3A). Volcano maps and Clustering heatmaps revealed that the differentially expressed gene FAT4 was significantly upregulated when UBE4B was knocked down in the TMT experiments (Fig. 3B, C). The UBE4B siRNA significantly reduced FAT4 protein levels in MKN45 cells. Consistent with these findings, overexpression of UBE4B in AGS cells significantly downregulated FAT4 protein levels (Fig. 3F). However, the real-time quantitative reverse transcription (qRT‒PCR) analysis showed that neither the knockdown nor overexpression of UBE4B affected FAT4 mRNA levels, suggesting that UBE4B downregulates FAT4 expression at the posttranscriptional level (Supplementary Fig. 1D, E). The bioconfidence analysis revealed that FAT4 was expressed at significantly lower levels in gastric cancer tissues from patients than in normal gastric mucosal tissues (Supplementary Fig. 1F). Moreover, FAT4 expression was correlated with the T stage, N stage and stage of GC patients (*p* < 0.05) (Supplementary Fig. 1G, H, I). Importantly, the KEGG enrichment analysis revealed that the Differentially Expressed Genes (DEGs) were enriched in autophagy-related pathways (Fig. 3D, E). As expected, knockdown of UBE4B induced autophagy in MKN45 cells, with a decrease in the level of the P62 protein and increases in the levels of the LCII/I proteins. The overexpression of UBE4B inhibited the occurrence of autophagy in AGS cells, upregulated P62 protein expression, and downregulated LCII/I protein expression (Fig. 3G). In addition, in MKN45 cells with UBE4B knockdown, autophagosome formation was significantly induced, as observed via transmission electron microscopy (Fig. 4A).

A cycloheximide (CHX) tracking assay was performed to verify whether UBE4B affects FAT4 expression, and the transfection of the UBE4B siRNA inhibited the degradation of the FAT4 protein in MKN45 cells (Fig. 4B, C). We further determined whether the reduction in FAT4 protein levels in siUBE4B-transfected cells occurred through proteasomal degradation. Treatment of siCtrl- or siUBE4B-transfected GC cells with the proteasome inhibitor MG132 revealed that the proteasome inhibitor promoted the upregulation of the FAT4 protein after UBE4B knockdown. These findings suggest that UBE4B interferes with the degradation of the FAT4 protein (Fig. 4D). In addition, UBE4B knockdown significantly downregulated FAT4 polyubiquitination (Fig. 4E). Moreover, the overexpression of UBE4B had the opposite effect on FAT4 (Fig. 4F). Taken together, these results suggest that UBE4B is a potent ubiquitinating enzyme that ubiquitinates and degrades FAT4.

### UBE4B exerts its oncogenic effects through FAT4

**Fig. 5:**
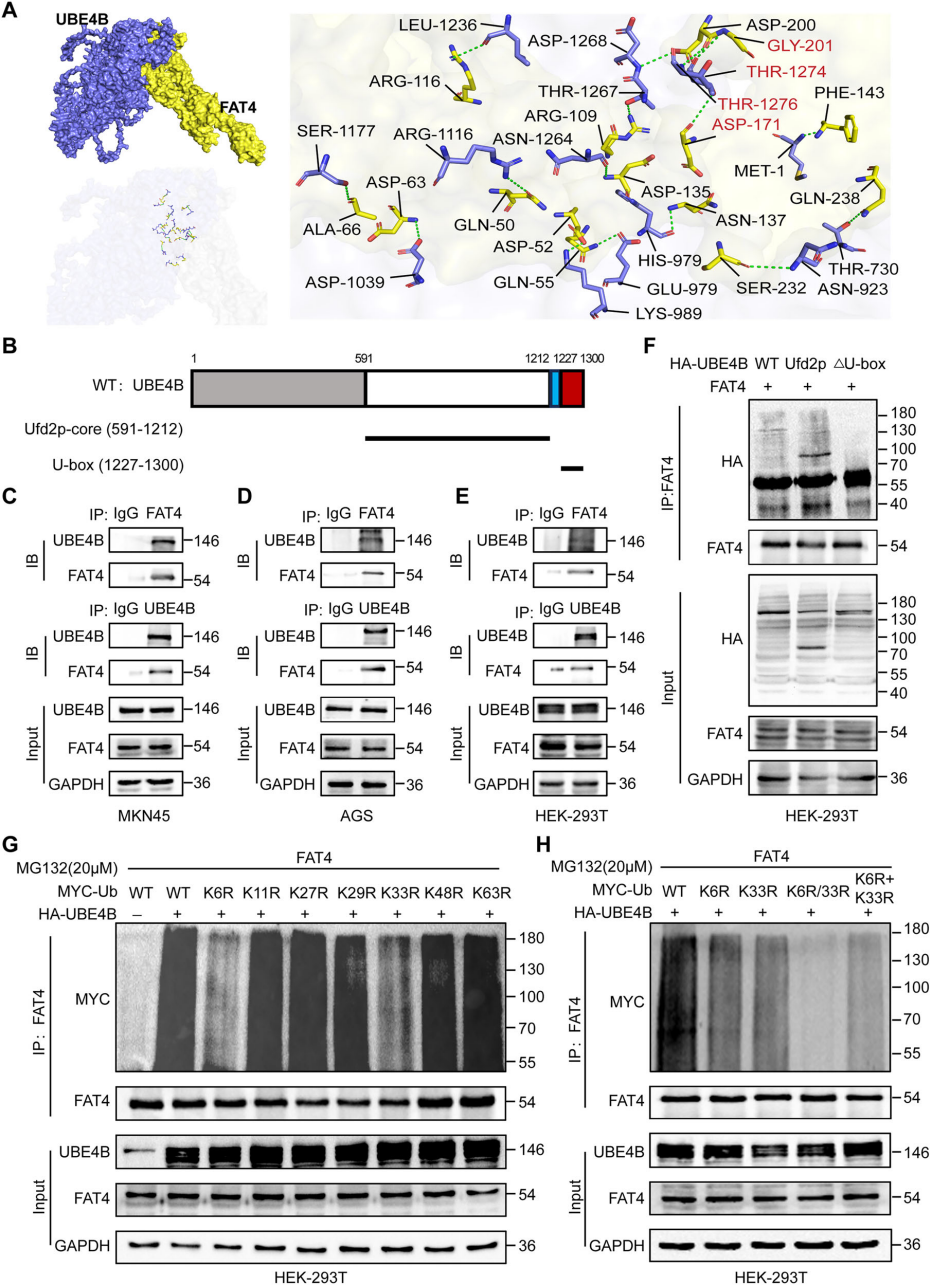
UBE4B interacts with FAT4 through ubiquitination. **A** Schematic representation of the docking structure of protein molecules. Blue color indicates UBE4B protein and yellow color indicates FAT4 protein. The key interacting residues are highlighted in red font. **B** Segment plasmid of UBE4B. **C**, **D** Endogenous UBE4B proteins were immunoprecipitated with an anti-UBE4B antibody and then analyzed by immunoblotting. Endogenous FAT4 proteins were immunoprecipitated with anti-FAT4 antibodies and then analyzed by immunoblotting. The IgG antibody was used as the control. **E** HEK-293T cells infected endogenous overexpression of FAT4 lentivirus and HA-UBE4B plasmid were subject to immunoprecipitation with anti-UBE4B and anti-FAT4 antibodies. **F** HEK-293T cells infected endogenous overexpression of FAT4 lentivirus were transfected with HA-UBE4B or several UBE4B mutants. A coimmunoprecipitation assay was performed to detect the interaction between UBE4B and FAT4 protein. **G** HEK-293T cells infected with endogenous overexpression of FAT4 lentivirus were transfected with HA-UBE4B or wild-type Ub, Ub-K6R, K11R, K27R, K29R, K33R, K48R, and K63R plasmid. Cell lysates were assayed for ubiquitination, and the ubiquitination level of FAT4 was detected. **H** HEK-293T cells infected with endogenous overexpression of FAT4 lentivirus were transfected with HA-UBE4B or wild-type Ub, Ub-K6R, K33R and K6R/K33R plasmid. Cell lysates were assayed for ubiquitination, and the ubiquitination level of FAT4 was detected.

We elucidated the mechanism of action by which UBE4B regulates FAT4 by performing molecular docking using PyMOL software and the GRAMM online webpage. UBE4B formed hydrogen bonds with FAT4 through amino acid residues such as THR 1274-GLY 201 and THR 1276-ASP 171, indicating that the UBE4B protein formed a stable interaction with the FAT4 protein (Fig. 5A). Endogenous interaction immunoprecipitation (Co-IP) assays revealed that UBE4B could interact with FAT4 (Fig. 5C, D). Furthermore, when exogenous HA-labelled UBE4B was transfected into HEK-293T cells endogenously overexpressing FAT4, HA-labelled UBE4B coimmunoprecipitated with FAT4 (Fig. 5E). These results strongly support the hypothesis that UBE4B interacts with FAT4 in GC cells. We constructed plasmids containing fragments of UBE4B to determine the exact region of UBE4B that interacts with FAT4 (Fig. 5B). Co-IP analysis showed that the U-box structural domain deletion mutant of UBE4B did not interact with FAT4, whereas the Ufd2p core structural domain interacted with FAT4. (Fig. 5F). We designed a series of plasmids with mutations in the ubiquitin site for ubiquitination studies. The results showed that UBE4B specifically mediates the ubiquitination of the FAT4 protein by facilitating the assembly of Lys-6- and Lys-33-linked polyubiquitin chains (Fig. 5G). Notably, the Co-IP results further suggest that the Lys6 and Lys33 sites are more likely to form hybrid ubiquitin chains rather than branched ubiquitin chains (Fig. 5H).

**Fig. 6:**
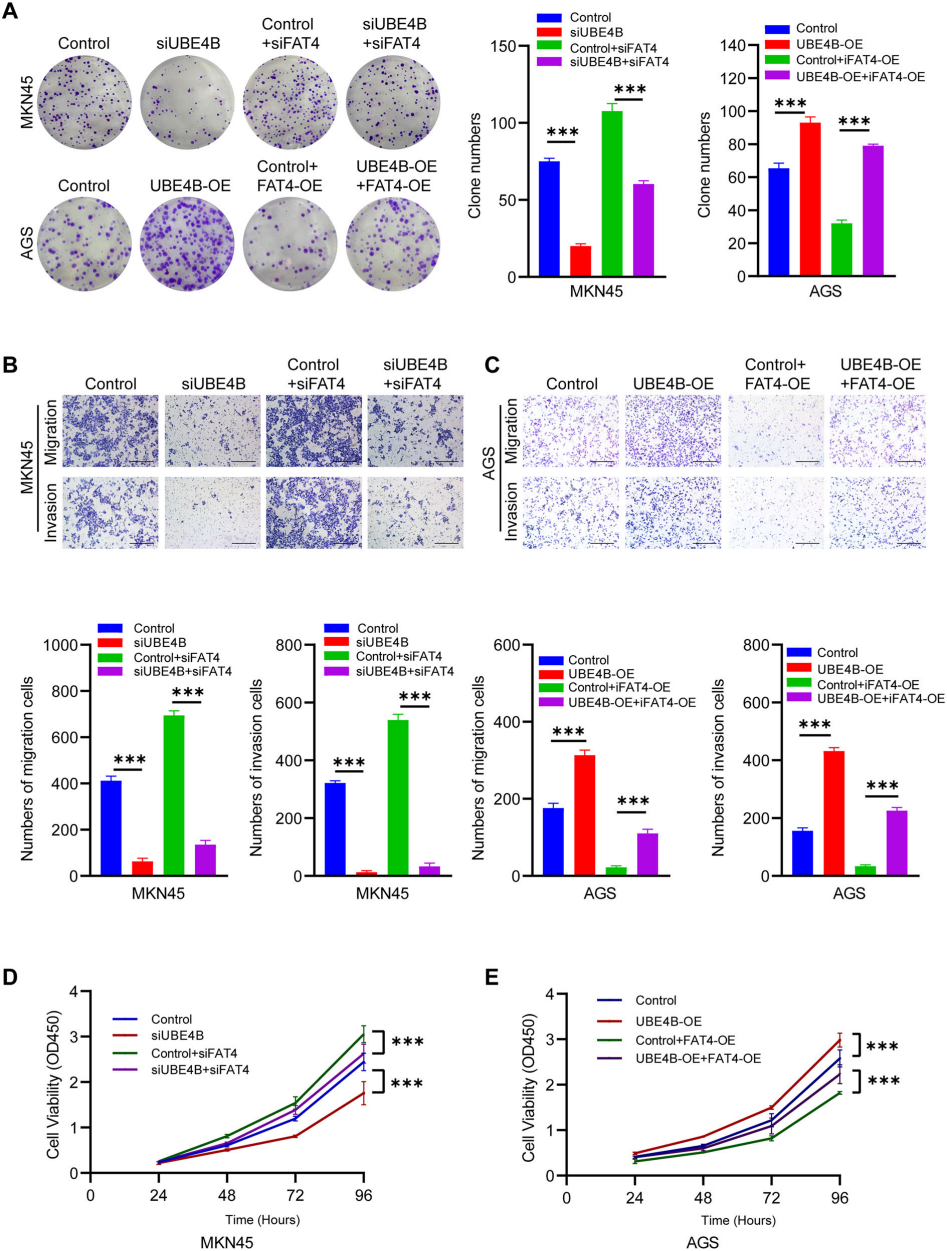
The carcinogenic effect of UBE4B is dependent on the degradation of FAT4. **A** Colony formation assay was performed to detect the proliferation of GC cells. **B** Invasion and migration assays were performed to detect the proliferation of MKN45 cells. Bar length: 100 μm. **C** Invasion and migration assays were performed to detect the proliferation of AGS cells. Bar length: 100 μm. **D** CCK-8 assay was used to detect the proliferation of MKN45 cells. **E** CCK-8 assay was used to detect the proliferation of AGS cells.

Moreover, we assessed the effects of UBE4B-mediated ubiquitination-induced downregulation and upregulation of FAT4 on UBE4B-induced GC cell proliferation. We designed four subgroups each for knockdown and overexpression, namely, the control group, UBE4B knockdown group, control group + FAT4 knockdown group, and UBE4B knockdown group + FAT4 knockdown group, as well as the control group, UBE4B overexpression group, control group + FAT4 overexpression group, and UBE4B overexpression group + FAT4 overexpression group, for subsequent cellular experiments. CCK-8 and colony formation assays revealed that downregulation of UBE4B reduced the proliferative capacity of MKN45 cells, whereas the knockdown of FAT4 restored the proliferative capacity of UBE4B-knockdown cells (Fig. 6A, D). Similarly, UBE4B overexpression increased the proliferative capacity of MKN45 cells, whereas the overexpression of FAT4 restored the proliferative capacity of UBE4B-overexpressing cells (Fig. 6A, E). Subsequently, transwell assays were performed to detect cell migration and invasion. The results revealed that downregulation of UBE4B reduced the invasion and migration of MKN45 cells, whereas the knockdown of FAT4 restored the invasion and migration of UBE4B-knockdown cells (Fig. 6B). Similarly, the upregulation of UBE4B increased the invasive and migratory abilities of MKN45 cells, whereas the overexpression of FAT4 restored the invasive and migratory abilities of UBE4B-overexpressing cells (Fig. 6C).

### UBE4B promotes tumour growth in vivo by inhibiting FAT4

**Fig. 7:**
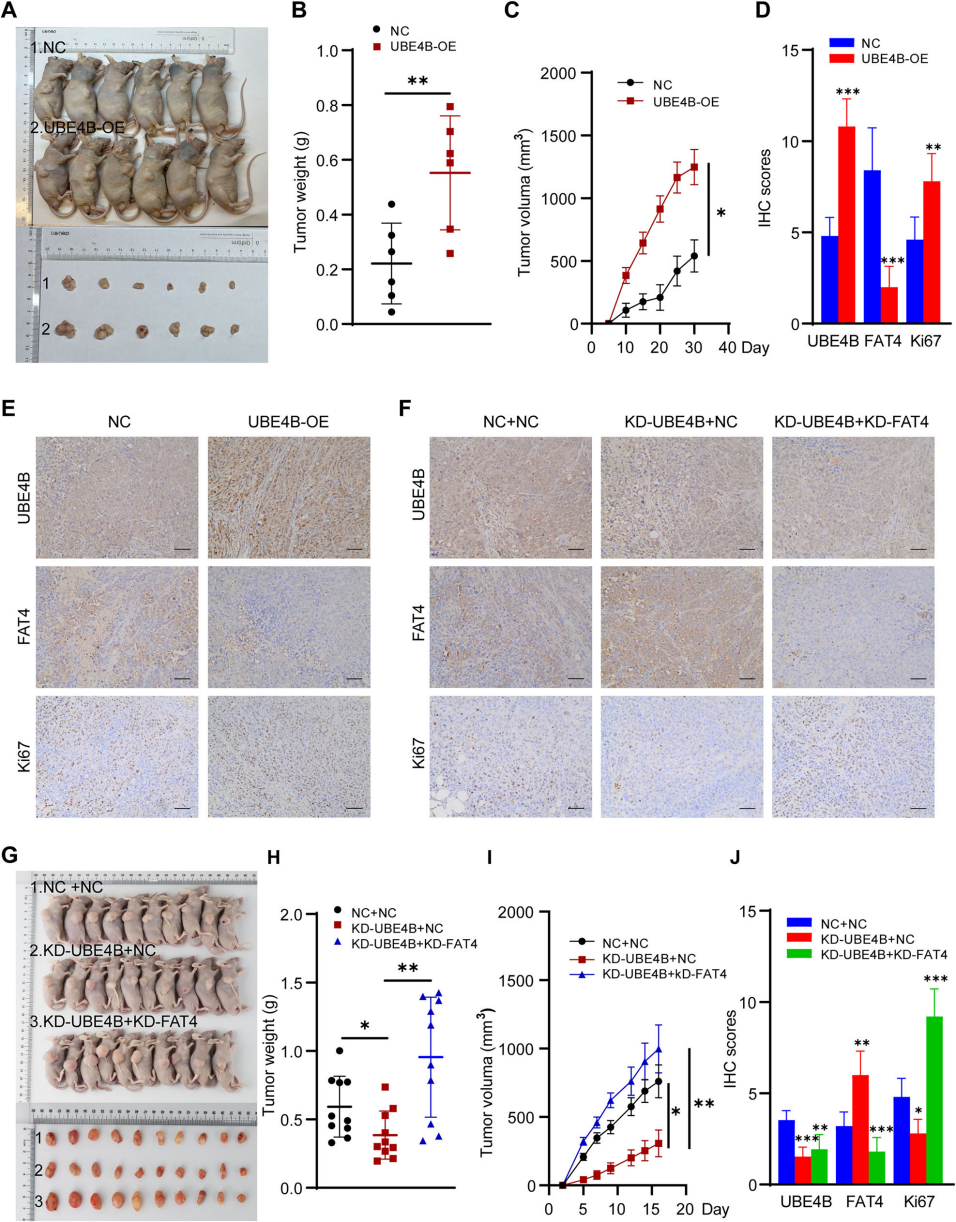
UBE4B downregulates FAT4 to promote GC cell proliferation in vivo. **A** MKN45 cells with/without shUBE4B were injected in nude mice. Representative images of xenograft tumors from mice bearing MKN45-shNC and MKN45-shUBE4B cells. **B** Tumor weights were calculated for each mouse in both groups (*n* = 6, Student’s t test). **C** Tumor volume was measured every 5 days (*n* = 6, Student’s t test). **D** IHC scores were analyzed for the expression levels of UBE4B, FAT4, and Ki67 in both groups (NC vs UBE4B-OE, *n* = 15, Student’s t test). **E**, **F** UBE4B, FAT4, and Ki67 expression in the tumor of nude mice were detected by IHC. Bar length: 50 μm. **G** MKN45 cells with/without shUBE4B or with/without FAT4 were injected in nude mice. Representative images of xenograft tumors from mice bearing MKN45-shNC+shNC, MKN45-shUBE4B+shNC, and MKN45-shUBE4B+shFAT4 cells. **H** Tumor weights were calculated for each mouse in the three groups (*n* = 10, One-way ANOVA). **I** Tumor volume was measured every 3 days (*n* = 10, One-way ANOVA). **J** IHC scores were analyzed for the expression levels of UBE4B, FAT4, and Ki67 in the three groups (NC + NC vs KD-UBE4B + NC vs KD-UBE4B + KD-FAT4, *n* = 15, One-way ANOVA).

MKN45 cells overexpressing UBE4B were injected subcutaneously into nude mice, and tumour volumes were measured every 3 days to verify the effect of UBE4B on cell growth in vivo (Fig. 7A). The tumour volume of the UBE4B-overexpressing group was significantly larger than that of the corresponding control group. In addition, the tumour weight in the control group was lower than that in the UBE4B-overexpressing group (Fig. 7B, C). IHC results revealed that the overexpression of UBE4B induced Ki67 expression and suppressed FAT4 expression (Fig. 7D, E). Thus, UBE4B promotes the growth of tumour cells in the body.

Three groups of nude mice were designed to further verify whether UBE4B affects cell growth in vivo by regulating FAT4 levels: a control group, a UBE4B knockdown group, and a UBE4B and FAT4 co-knockdown group (Fig. 7G). The tumour volume of the UBE4B knockdown group was significantly smaller than that of the corresponding control group, and the tumour volume of the UBE4B and FAT4 co-knockdown group was significantly larger than that of the group with UBE4B knockdown alone. In addition, the tumour weight of the UBE4B knockdown group was lower than that of the control group, and the tumour volume of the UBE4B and FAT4 co-knockdown group was significantly larger than that of the UBE4B knockdown group (Fig. 7H, I). Immunohistochemical results revealed that the knockdown of UBE4B inhibited Ki67 expression and induced FAT4 expression. Knockdown of FAT4 restored the expression of KI67 and UBE4B (Fig. 7F, J). In conclusion, we demonstrated that UBE4B not only promotes the growth of GC tumours in vivo but also plays a corresponding role by regulating FAT4 levels.

### High expression of UBE4B and low expression of FAT4 in clinical GC samples predict poor survival outcomes

**Fig. 8:**
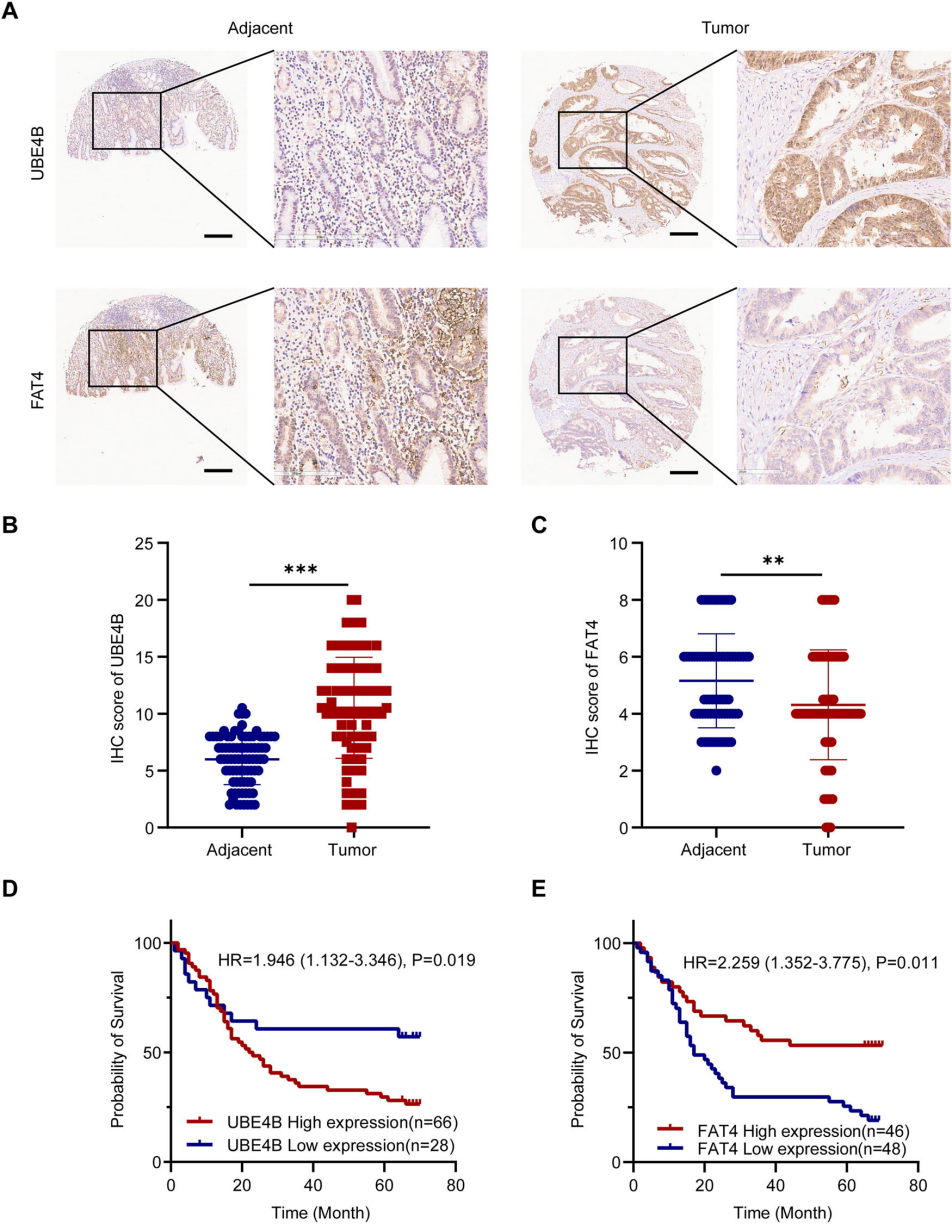
UBE4B was highly expressed in clinical GC samples, and patients had a poor prognosis, in contrast to FAT4. **A** Representative image of IHC staining of tumor microarrays from GC patients. Bar length: 50 μm. **B** IHC scores of UBE4B level between GC tissue and adjacent normal tissue. **C** IHC scores of FAT4 level between GC tissue and adjacent normal tissue. **D** The Kaplan-Meier plot of overall survival by the expression of UBE4B in tumor microarrays related information (*n* = 66 (high-expression) vs 28 (low-expression)). **E** The Kaplan-Meier plot of overall survival by the expression of FAT4 in tumor microarrays related information (*n* = 46 (high-expression) vs 48 (low-expression)).

Tumour microarrays (TMAs) were used to analyse the expression of UBE4B and FAT4 in the tumour and adjacent normal tissues of 94 patients with gastric cancer, as well as the corresponding clinicopathological information. UBE4B was highly expressed in most tumour samples, whereas FAT4 was expressed at low levels (Fig. 8A). IHC scores revealed that the expression level of UBE4B in GC tissues was significantly higher than that in adjacent normal tissues, whereas the expression level of the FAT4 protein was significantly lower than that in adjacent normal tissues (Fig. 8B, C). An analysis of the clinicopathologic findings revealed that high UBE4B expression was associated with nerve invasion (*p* = 0.031) and lymph node metastasis (*p* = 0.029; Table 1). We further explored the prognostic impact of the expression of these proteins on the survival outcomes of GC patients. The results revealed that patients with high UBE4B expression had a poorer prognosis than those with low UBE4B expression. The opposite result was observed for the FAT4 protein (Fig. 8D, E).

**Table 1:** Correlation between UBE4B protein expression and clinicopathologic features of GC.

Variables	Total (*n* = 94)	UBE4B expression level		*P*-value
		High (*n* = 64)	Low (*n* = 30)	
Age				0.766
≤60	27	19	8	
>60	67	45	22	
Sex				0.199
Female	35	21	14	
Male	59	43	16	
Tumor type				0.760
Protuberant	7	4	3	
Infiltrative	24	17	7	
Ulcerative	63	43	20	
Tumor grade				0.220
I–II	40	30	10	
III	54	34	20	
TNM staging				0.594
I/II	37	24	13	
III/IV	57	40	17	
Tumor size				0.382
≤5 cm	47	34	13	
>5 cm	47	30	17	
Lymphovascular invasion				0.059
Yes	29	23	6	
No	65	41	24	
Nerve invasion				0.031
Yes	14	13	1	
No	80	51	29	
Lymph node metastasis				0.009
Yes	22	10	12	
No	72	54	18	
Distant metastasis				0.333
Yes	2	2	0	
No	92	62	30	

## Discussion

In this study, we showed that UBE4B was highly expressed in GC and was associated with a poorer prognosis. In addition, the upregulation of UBE4B expression was correlated with the age and TNM stage of GC patients. Experiments assessing cellular functions revealed that the knockdown of UBE4B significantly inhibited cell proliferation, colony formation, migration, and invasion. Therefore, UBE4B has a tumour-promoting role in GC and may be a valuable prognostic biomarker for GC patients. We further explored the detailed mechanism by which UBE4B acts as an E3 ligase to ubiquitinate substrates and promote GC malignant behaviour through its U-box structural domain. UBE4B can degrade FAT4 via ubiquitination, thereby downregulating the expression of this protein. This ubiquitination-mediated downregulation of FAT4 expression promotes gastric cancer cell proliferation, migration and invasion in gastric cancer cells. Low expression of FAT4 is an important mechanism by which UBE4B promotes a poor prognosis for GC patients. Therefore, UBE4B may serve as a novel GC target that promotes GC cell progression in vitro and in vivo, and the ubiquitination and degradation of FAT4 exert oncogenic effects.

The FAT cadherin family members exhibit a similar extracellular structural domain structure with a single-pass transmembrane receptor composed of 32–34 cadherin repeats. These four distinct cytoplasmic structural domains may reflect the specific function of each FAT cadherin member [31]. Based on the results of the pancancer analysis, FAT4 has been recognized as a novel prognostic biomarker for various cancers. Notably, in lung and colorectal cancers, the by which FAT4 inhibits tumour growth is closely related to the regulation of tumour autophagy. Yang et al. reported that autophagy could be promoted and that the epithelial‒mesenchymal transition (EMT) process could be inhibited in lung cancer cells by activating FAT4 [30]. In addition, Wei et al. showed that FAT4 regulates the EMT and autophagy in colorectal cancer cells partly through the PI3K-AKT signalling pathway [29]. However, to the best of our knowledge, studies of FAT4-related posttranslational modifications that promote its degradation have not been reported. Here, we describe the role of FAT4 as a substrate for ubiquitination and the related mechanisms.

Autophagy is a mechanism for maintaining homeostasis and a response to stress. It engulfs, digests, and recycles cellular proteins, organelles, and cytoplasmic components through the lysosomal degradation pathway to maintain cellular metabolic processes. Recent studies have shown that autophagy plays dual roles in tumorigenesis and development [32]. On the one hand, the homeostatic function of autophagy restricts genomic damage events that favour tumour initiation, thus inhibiting tumorigenesis and progression; on the other hand, it can help cells reduce stress, which promotes the progression of advanced tumours [33, 34]. In this study, knockdown of UBE4B upregulated FAT4 expression and promoted autophagy in GC cells. Thus, we determined that UBE4B inhibits autophagy in GC cells and that it regulates GC progression by mediating FAT4 ubiquitination.

UBE4B, the human homologue of *Saccharomyces cerevisiae* UFD2, contains a conserved U-box catalytic structural domain, which is the key structure for its ligase activity [10]. The initial findings described UBE4B as an E4 factor that promotes polyubiquitination of hypo-ubiquitinated proteins via the synergistic action of E1-activating enzymes, E2-conjugating enzymes, and E3 ligases and further degradation by the 26S proteasome system. Researchers subsequently reported that UBE4B is structurally similar to the E3 ligase with a RING structure and showed that it can act independently of the E3 ligase for polyubiquitination; these studies suggest that UBE4B possesses E3 ligase activity [35]. Previous studies have shown that UBE4B is expressed predominantly in mouse neuronal tissues and is one of the major factors regulating the development of the nervous system. In recent years, an increasing number of scholars have focused on the role of UBE4B in cancer, and studies have shown that the UBE4B gene is overexpressed or repressed in many types of cancer. In hepatocellular carcinoma, the upregulation of UBE4B expression is associated with a poor prognosis and tumour immune infiltration [36]. In addition, UBE4B promotes lung adenocarcinoma development by enhancing cell proliferation, migration, and glycolysis through the PP2A/AKT signalling pathway [37]. Instead, abnormal downregulation of UBE4B gene expression has been observed in neuroblastoma and oral squamous cell carcinoma. However, no study has investigated the expression of UBE4B in gastric cancer. Here, our findings reveal a novel mechanism by which UBE4B mediates the ubiquitination and degradation of FAT4 to promote gastric cancer progression.

Multiple ubiquitination and degradation targets have been identified for UBE4B, including ataxin-3 [16], FEZ1 [38], p73 [30], EGFR [29], p53 [32], and MAPK1 [17]. These findings reveal the role of UBE4B in the ubiquitination and degradation of several proteins and provide clues to improve our understanding of the importance of UBE4B in the regulation of cell function and tumour development. Several previous studies have reported the role and specific mechanism of UBE4B-mediated degradation of ubiquitinated P53 [39–42]. Notably, a recent study identified a SWIB/Hdm2 motif in UBE4B. This UBE4B motif blocks the p53-UBE4B interaction and activates p53 functions, including p53-dependent transactivation and growth inhibition [39]. FAT4 has been repeatedly reported as a potential new target for GC therapy, but whether FAT4 expression is posttranslationally regulated through the ubiquitin‒proteasome pathway is not yet known. In this study, we identified the interaction of the U-box structural domain in UBE4B with FAT4 as an important molecular mechanism that regulates gastric cancer progression. To our knowledge, this study is the first to examine UBE4B expression and function in GC.

The UPS system includes ubiquitin, the ubiquitin-activating enzyme E1, the ubiquitin-conjugating enzyme E2, the ubiquitin ligase E3, and the 26S proteasome [5]. Ubiquitin polypeptides contain seven lysine residues (Lys6, Lys11, Lys27, Lys29, Lys33, Lys48, and Lys63), which can serve as sites for interlocking with target proteins to form ubiquitin chains of different lengths and conformations [43]. Generally, polyubiquitination reactions occur on Lys48 residues, and proteins tagged with Lys48-linked ubiquitin chains are degraded by the proteasome [44]. However, modifications involving the Lys63-linked chain are involved in intracellular transport and the localization and activity of multiple kinases and signalling pathways [45]. Although the existence and importance of most of the possible chain types have been determined, the signalling consequences and regulatory factors of half of them (K6, K27, K29, and K33) are still unclear and are therefore referred to as “atypical” chains [46]. In the present study, our results indicate that UBE4B specifically mediates the ubiquitination of FAT4 protein by promoting the assembly of Lys-6- and lys-33-linked polyubiquitin chains.

Branched Ub was first identified as early as 2003, accounting for 5-20% of the cellular polyubiquitin chain [47]. Existing studies have found that the K48/K63 branched chain can enhance NF-kB signaling [48] and TRIP12 promotes small molecule-induced degradation via K29/ k48 branched ubiquitin chains [49]. Mass spectrometry is the gold standard for the characterization of ubiquitin chain topology. Notably, recent studies have found that extended inhibitor treatment detected UBE4B-mediated polyubiquitin chain extension by CO-IP, specifically mediating polyubiquitination modification of the Lys48/Lys63 branch [50]. Similarly, this study was conducted by constructing Ub double mutants for CO-IP experiments to demonstrate the type of ubiquitin chain that connects the Lys6 and Lys33 sites. We believe that the Lys6 and Lys33 sites are more likely to form hybrid ubiquitin chains than branched ubiquitin chains. Taken together, these findings help to elucidate the relationship between the molecular conformation and functional polymorphism of UBE4B. In addition, the mechanism of UBE4B-mediated ubiquitination was further elucidated. In vivo animal experiments revealed that the knockdown of UBE4B inhibited tumour growth, whereas the opposite effect was observed after the overexpression of UBE4B. This growth inhibition could be reversed by FAT4 knockdown, suggesting that the regulation of gastric cancer by UBE4B depends on FAT4. In addition, FAT4 was expressed at lower levels in GC tissues than in neighbouring normal tissues. A subsequent survival analysis revealed that low expression of FAT4 was associated with a poor prognosis for GC patients. In this study, the role of UBE4B in gastric carcinogenesis and the molecular mechanism by which it ubiquitinates and degrades FAT4 are described for the first time.

## Conclusions

**Fig. 9:**
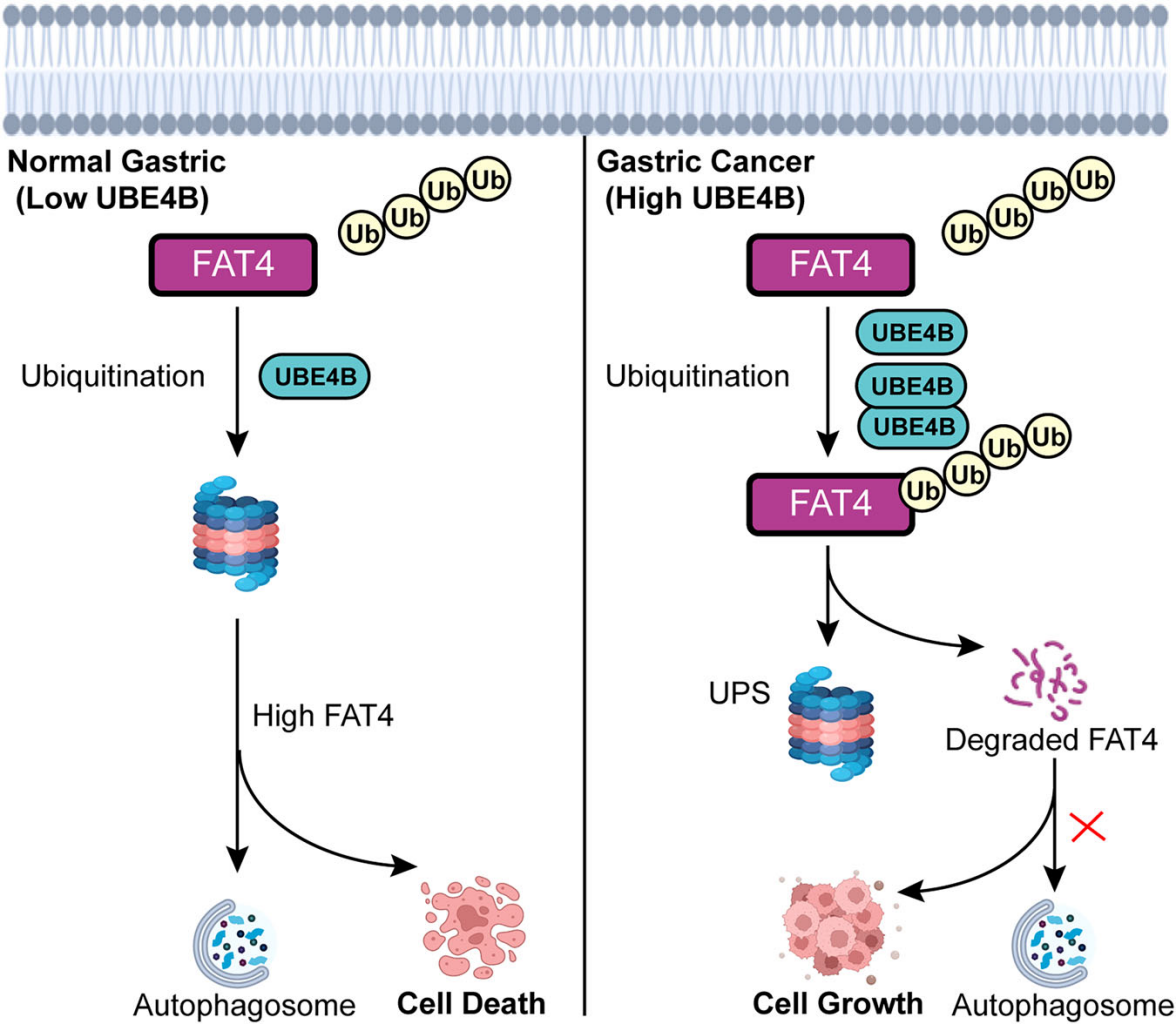
Scheme for the regulatory mechanism of UBE4B on FAT4. Created with BioRender.com.

In summary, the upregulation of UBE4B resulted in the degradation of the FAT4 protein and promoted GC growth (Fig. 9). High expression of UBE4B in GCs was correlated with a poor survival prognosis and clinicopathological features. UBE4B upregulation promoted proliferation, colony formation, migration and invasion. Further studies revealed that UBE4B directly interacted with FAT4, which in turn promoted the K6- and K33-linked ubiquitination of FAT4, thereby degrading and downregulating FAT4. The upregulation of FAT4 reversed the effect of UBE4B on promoting the growth of GC cells. UBE4B interacted with the FAT4 protein and promoted its polyubiquitination and protease-dependent degradation in GC cells. These findings suggest that UBE4B may serve as a new prognostic biomarker and therapeutic target for GC.

## Materials and methods

### Cell culture, antibodies, and reagents

AGS cells were purchased from the Chinese Academy of Sciences (Beijing, China). HGC-27, MKN45 and HEK-293T cells were purchased from Pricella (Wuhan, China). MKN-74 cells, SGC-7901 cells and human gastric mucosal epithelial cells (GES-1) were purchased from the Beijing Institute of Cancer Research (Beijing, China). The GC cells (MKN-45, MKN-74, SGC-7901 and HGC-27) and GES-1 cells were maintained in RPMI-1640 medium (Gibco Company, Massachusetts, USA) containing 10% foetal bovine serum (FBS) 100 units/mL penicillin (P) and 100 g/mL streptomycin (S) in a humidified atmosphere at 37 °C with 5% CO_2_. AGS cells were cultured in Ham’s F-12 medium (Gibco Company, USA) supplemented with 10% FBS and 1% P/S. HEK-293T cells were cultured in Dulbecco’s modified Eagle’s medium (DMEM) (Gibco Company, USA) supplemented with 10% FBS and 1% P/S. The antibodies used in this study were as follows: UBE4B (18148-1-AP, Proteintech), FAT4 (A17212, ABclonal), FAT4 (NBP1-78381, Novus), P62 (#7695, Cell Signaling), LC3 (#12741, Cell Signaling), ubiquitin (10201-1-AP, Proteintech), Ki67 (#9449, Cell Signaling), HA (20543-1-AP, Proteintech), MYC (390003, Zenbio), and GAPDH (HC301-01, TransGen Biotech).

### Patient samples, tissue microarray, and immunohistochemistry

A total of 40 samples of gastric cancer (*n* = 20) and paracancerous (*n* = 20) tissues were obtained from the Department of Pathology of the First Affiliated Hospital of Nanchang University. A TMA was constructed using GC tissues and corresponding normal gastric tissues (Outdo Biochip, Shanghai, China). IHC staining was performed to detect UBE4B and FAT4 expression. The percentage of positively stained cells and the intensity of IHC staining were assessed by two senior pathologists. The scoring result was the positive cell staining score × the staining intensity score, and the average of the two physicians’ scores was recorded as the final score. The percentage of positively stained cells was scored into 5 grades: 0 (1–5%), 1 (6–25%), 2 (26–50%), 3 (51–75%) and 4 (76–100%). The staining intensity scores were divided into 4 levels: 0 for no colour, 1 for a light yellow colour, 2 for a dark yellow colour, and 3 for a tan colour. The final scoring results were blindly assessed by 2 pathologists and categorized into 3 groups: high expression (7–9), moderate expression (4–7), and low expression (0–3). This study was approved by the Institutional Human Ethics Committee of the First Affiliated Hospital of Nanchang University.

### Plasmid construction, small interfering RNAs, and lentivirus transfection

The empty vector, HA-UBE4B, and UBE4B domain deletion mutant plasmids and the Myc-tagged Ub-K6R, Ub-K11R, Ub-K27R, Ub-K29R, Ub-K33R, Ub-K48R, Ub-K63R, and Ub-K6R/33R plasmids were purchased from MIAOLING BIOLOGY (Wuhan, China). The UBE4B and FAT4 siRNAs were purchased from GenePharma (Suzhou, China). The sequences of the siRNAs used for UBE4B were as follows: siRNA1, sense (5′-3′) GCCCUCUAAUAGCCUUGAATT, antisense (5′-3′) UUCAAGGCUAUUAGAGGGCTT; siRNA2, sense (5′-3′) GCAGUUUGU UCGCUA UAUATT, antisense (5′-3′) UAUAUAGCGAACAAACUGCTT; and siRNA3, sense (5′-3′) CUGCAAUGCUGAACUUUAATT, antisense (5′-3′) UUAAAGUUCAGCAUUGCAGTT. The siRNAs for the FAT4 sequence were as follows: sense (5′-3′) GCAGGUGUCUAUUAGUCAATT and antisense (5’-3’) UUGACUAAUAGACACCUGCTT. Lentiviruses for UBE4B knockdown (shRNA sequence: GCAGGGATCAAATCCACAATA), lentiviruses for UBE4B overexpression, and lentiviruses for FAT4 knockdown (shRNA sequence: CCTGACAATATCCCTCCCTAT) were generated by Genechem Co., Ltd. (Shanghai, China). The deficient Cas9-synergistic activation mediator (Cas9-SAM) system for the endogenous overexpression of the FAT4 mRNA was purchased from Genechem (Shanghai, China). dCas9-VP64-Puro was used to express wild-type dCas9, and sgRNA-MS2-P65-HSF1-Neo was used to target the FAT4 promoter region. The sequences of the sgRNAs of Cas9-SAM were as follows: sgRNA1, CTGCTTCAGCTCCGGCCCAA; sgRNA2, GGCTGTAGGCGGTCTGGTGT; and sgRNA3, TAGCATCCCGAGAAGCCAGT. The day before lentivirus transduction, the specified cell lines were grown in 6-well plates at a density of 5 × 10^4^ cells/well (20–30% density). dCAS9-VP64 lentiviral particles were added on the day of infection to infect the cells. Puromycin screening was performed 3 days after the infection, and after the screened cells were in a stable state, they were reinfected with the sgRNA-MS2-P65-HSF1 lentivirus and screened with G418, after which the cells were collected for the detection of overexpressed proteins via western blotting.

### Real-time qRT‒PCR

Total RNA was prepared using the MolPure Cell RNA Kit (Yeasen Biotechnology, Shanghai, China), and 1 µg of RNA was reverse transcribed into cDNA using the FastKing cDNA First Chain Synthesis Kit (TIANGEN, Beijing, China). A Hifair AdvanceFast One-step RT-gDNA Digestion SuperMix Kit (Yeasen Biotechnology, Shanghai, China) was subsequently used to perform real-time PCR with a real-time PCR system (Quant Studio 5). All reagents and conditions for qPCR followed the instructions of the kit. The forward and reverse PCR primers for UBE4B were 5′-CTACCTCCCCAATAGGTGCAT-3′ and 5′-GGCGAGCTGCTGAGAGAAC-3′, and those for FAT4 were 5′-TCACATCCCATCTCCTACTACTTT-3′ and 5′-CTTCTGAAGCTCCGTTTAGTG-3′′.

### Coimmunoprecipitation and western blot analysis

The interaction of UBE4B with FAT4 was determined via co-IP. Gastric cancer cells were lysed in RIPA buffer (Solarbio Life Science, Beijing, China) supplemented with protease and phosphatase inhibitors (TransGen Biotech, Beijing, China). A total of 500 μl of centrifuged lysate was incubated with 2 μl of the primary antibody and 800 µl of phosphate-buffered saline (PBS) for 2 h. Then, 80 μl of Protein A/G agarose (Santa Cruz Biotechnology, Texas, USA) mixed with PBS after centrifugation was added to the cellular protein–antibody complex and incubated at 4 °C overnight. The next day, the cell‒antibody complex was centrifuged at 5000 rpm at 4 °C and washed twice with RIPA lysis buffer containing protease and phosphatase inhibitors. The immunoprecipitated proteins were eluted by boiling in SDS‒PAGE loading buffer for 5 min. Proteins from the lysate were separated by SDS–PAGE, transferred to a nitrocellulose membrane, blocked with 5% BSA (Solarbio Life Science, Beijing, China) for 1 h, and then incubated overnight at 4 °C with the corresponding primary antibody. The following day, the samples were incubated with a horseradish peroxidase-conjugated secondary antibody (TransGen Biotech, Beijing, China) for 1 h at room temperature.

### Mass spectrometry

The total proteins were extracted from siUBE4B and siNC gastric cancer cells, a portion was removed to determine the protein concentration and for SDS‒PAGE, and another portion was removed for trypsin digestion and labelling. An equal amount of each labelled sample was then mixed for chromatographic separation, and finally, LC‒MS/MS analysis and data analysis were performed. TMT was performed and analysed by Luming Biotechnology (Shanghai, China).

### Biological transmission electron microscopy

Precipitates of 10^7^ gastric cancer cells were collected, immediately added to glutaraldehyde and fixed at 4 °C. After 12–24 h, the samples were rinsed three times with 0.1 M phosphate-buffered saline (PB) (pH 7.2) and fixed with a 1% osmium solution for 2 h. The osmium acid waste solution was removed, the samples were rinsed with 0.1 M phosphate-buffered saline (PB) for 15 min, and the samples were washed with water three times. The tissue was sequentially dehydrated in 30%, 50%, 70%, 80%, 95%, and 100% ethanol every 15 min, and dehydrated in 100% acetone 3 times for 20 min each. Then, the tissue was embedded in acetone and embedding agent. The embedded tissues were polymerized in an oven at 60 °C for 48 h. The resin blocks were cut into 60–70 nm ultrathin sections using an ultrathin slicer. The samples were stained with uranyl acetate for 25 min, washed with water, stained with lead citrate for 7 min, and then washed with water. Transmission electron microscopy (Hitachi HT-7800) was performed at 80 kV, and images were captured for analysis.

### Cell proliferation, colony formation, and transwell assays

CCK-8, colony formation, and transwell assays were performed to monitor cell proliferation, migration, and invasion. Each experiment was repeated at least three times. For the CCK-8 analysis, approximately 2000 GC cells were cultured in each well of a 96-well plate. The next day, gastric cancer cells were transfected with the UBE4B siRNA or the UBE4B overexpression plasmid. Then, cell viability was determined by performing a CCK-8 (Yeasen Biotechnology, Shanghai, China) assay and measuring the absorbance at 450 nm. For the colony formation assay, 800 GC cells were cultured in each well of 6-well plates. After 2 weeks, the cell colonies on the plates were fixed with 4% paraformaldehyde and stained with 1% crystal violet. ImageJ was used to count the number of cell colonies. For the transwell assay, the migration and invasion abilities of gastric cancer cells were evaluated in Transwell coated with or without Matrigel (Corning, USA). A total of 4 × 10^4^ AGS cells and 6 × 10^4^ MKN45 cells were inoculated in 200 μl of serum-free medium and seeded on transwells precoated or uncoated with Matrigel, respectively. At the same time, medium containing 20% FBS was added to the lower chamber. After 48 h, the migrated or invaded cells were fixed with 4% paraformaldehyde for 20 min and stained with 1% crystal violet for 30 min. The number of invading cells in ten random fields of view in each chamber was counted under a microscope (Olympus, Tokyo, Japan).

### Ubiquitination assay

HEK293T cells endogenously and stably overexpressing FAT4 were constructed, and the stably transfected line was transfected with MYC-Ub or HA-UBE4B for 48 h. After treatment with 20 μM MG132 (MedChemExpress, New Jersey, USA) for 6 h, cell lysates were prepared with cell lysis buffer and immunoprecipitated overnight with the indicated antibodies on Protein A/G beads (Santa Cruz Biotechnology, Texas, USA). The beads were then added to SDS sample buffer and boiled at 100 °C for 5 min. Immunoprecipitated protein complexes were detected via immunoblotting with an anti-MYC antibody.

### Animal study

Six-week-old female BALB/c nude mice were purchased from GemPharmatech LLC (Nanjing, China). The animal experiments were divided into two batches. The first batch of 20 female thymus-free BALB/c nude mice was divided into control and UBE4B overexpression groups. In the second batch, 30 nude mice were divided into a control group, a UBE4B knockdown group, and a UBE4B and FAT4 co-knockdown group. MKN45 cells (5 × 10^7^ cells) were transfected with lentivirus and injected subcutaneously into the respective groups. After the injection, the behaviour and health of the nude mice were observed every 2–3 days, and body weights and tumour sizes were recorded. Vernier callipers were used to measure the long and short diameters of the tumour and to calculate the volume of the tumour (V = 1/2 × length × width²). After the mice were sacrificed, the subcutaneous tumours were excised, fixed with 4% FPA, and subjected to immunohistochemical staining. All animal experiments were approved by the Laboratory Animal Welfare Ethics Committee of the First Affiliated Hospital of Nanchang University (CDYFY-IACUC-202404QR003).

## Supplementary information

supplementary data

original gel data

## Data Availability

The datasets generated during and/or analysed during the current study are available from the corresponding author on reasonable request.
