# Supplementary material for: UBE4B promotes gastric cancer proliferation and metastasis by mediating FAT4 ubiquitination and degradation
Source: Cell Death Dis. 2025 Jul 23;16(1):551. doi: 10.1038/s41419-025-07794-8 (PMC12287395; doi:10.1038/s41419-025-07794-8)
Supplement: Supplementary file 1 — supplementary data [file 41419_2025_7794_MOESM1_ESM.docx]

**Supplementary materials**

**
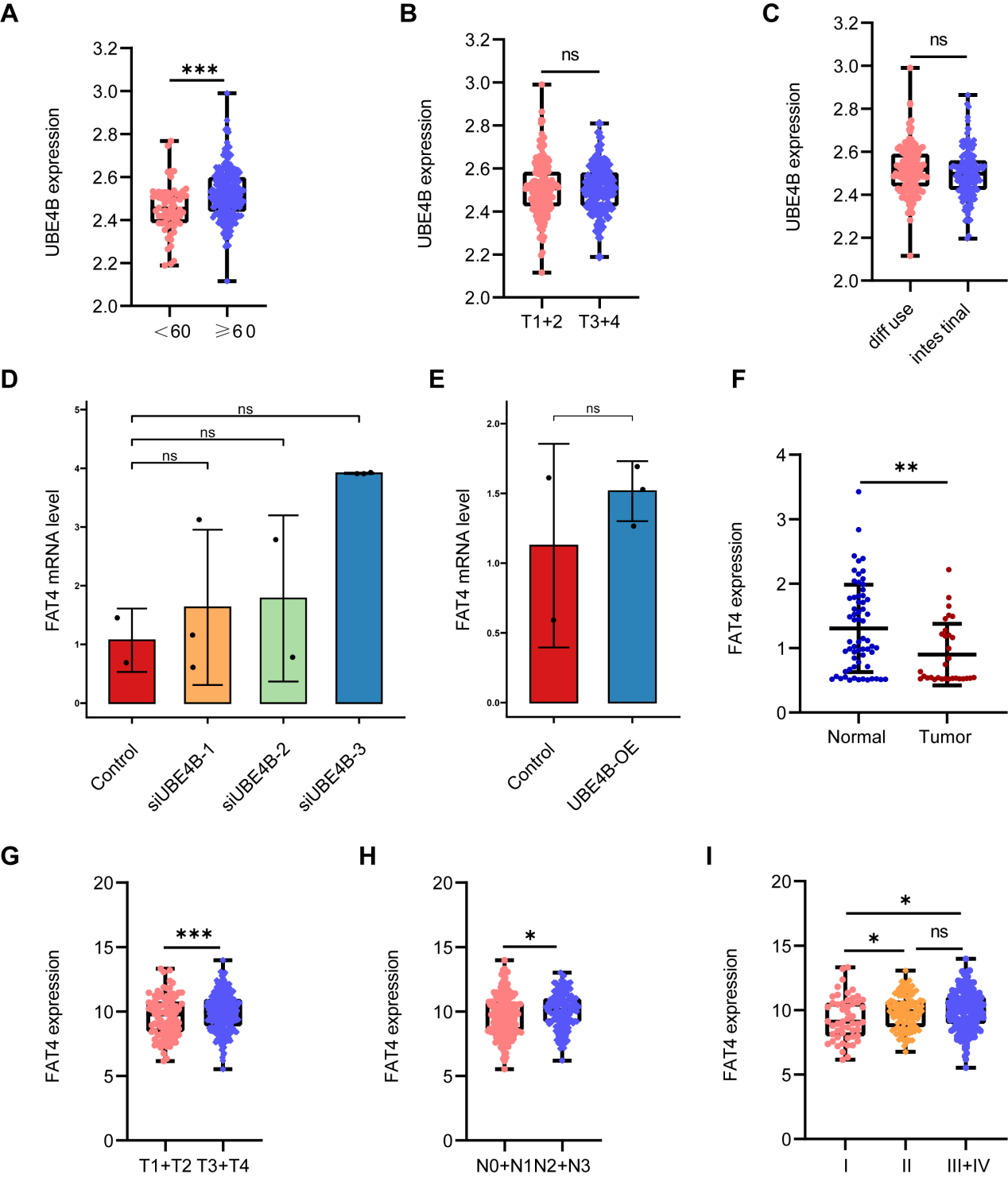
**

**Supplementary Fig 1: A B C** Clinicopathologic features of UBE4B expression and age, T-stage and different types in GC patients, the data was carried out from the GEO database. **D E** The Real-time quantitative reverse transcription (qRT-PCR) was performed to analyze the mRNA levels of FAT4 after UBE4B knockdown and overexpression. **F** The real-time PCR assay of the mRNA expression of FAT4 in GC tissues and normal tissues from the GEO database. **G H I** Clinicopathologic features of FAT4 expression and T-stage, N-stage and Stage in GC patients, the data was carried out from the GEO database.
