# Supplementary material for: UBE4B promotes gastric cancer proliferation and metastasis by mediating FAT4 ubiquitination and degradation
Source: Cell Death Dis. 2025 Jul 23;16(1):551. doi: 10.1038/s41419-025-07794-8 (PMC12287395; doi:10.1038/s41419-025-07794-8)

**Fig1**

1C GAPDH


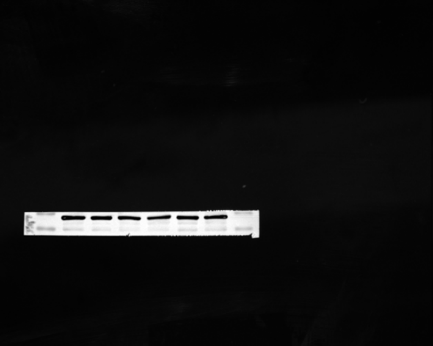


1C UBE4B


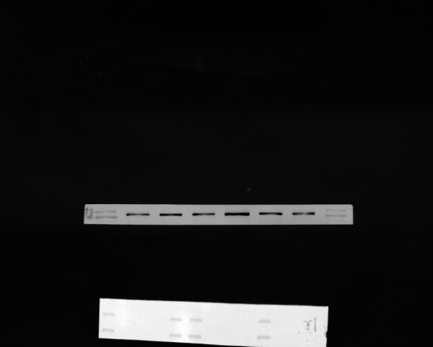


**Fig 3**

3F MKN45 GAPDH


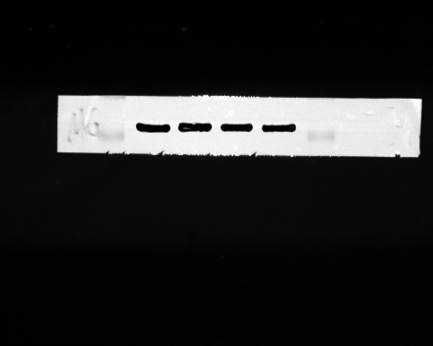


3F MKN45 FAT4


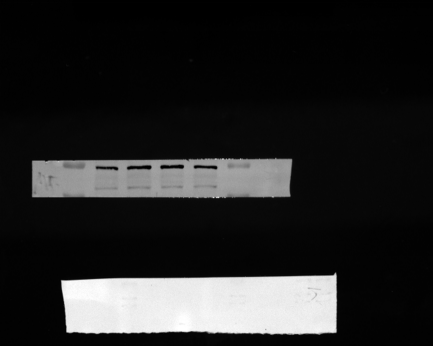


3F MKN45 UBE4B


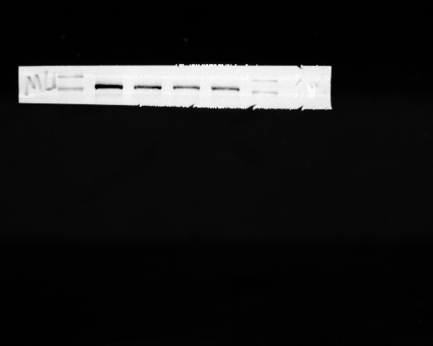


3F AGS GAPDH


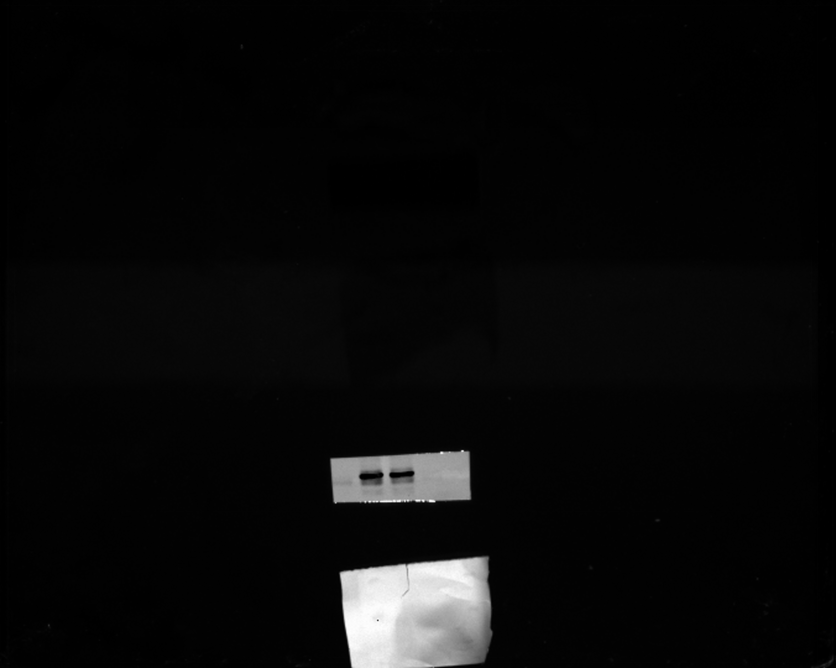


3F AGS FAT4


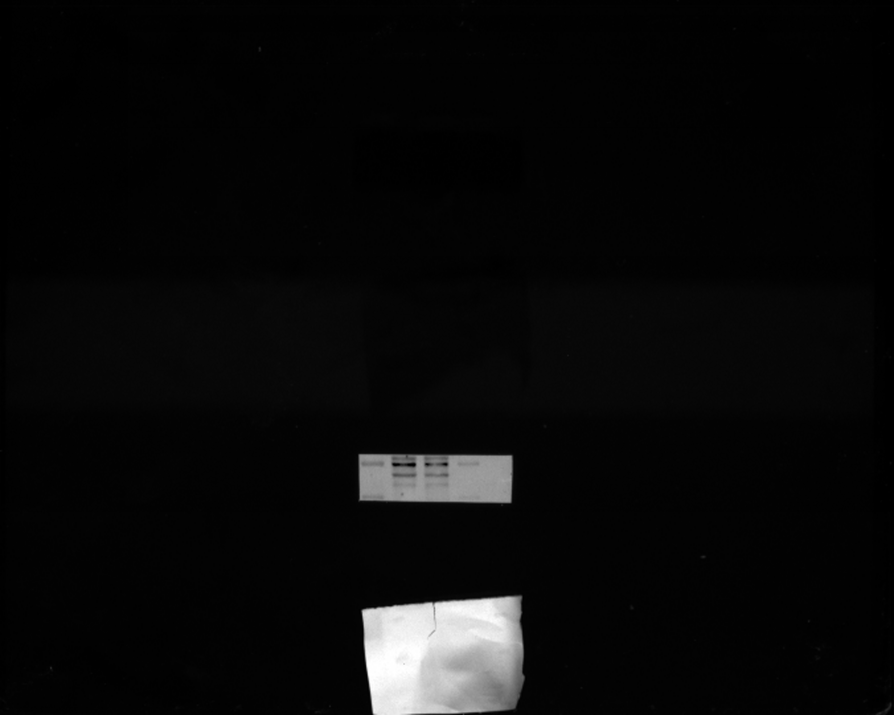


3F AGS UBE4B


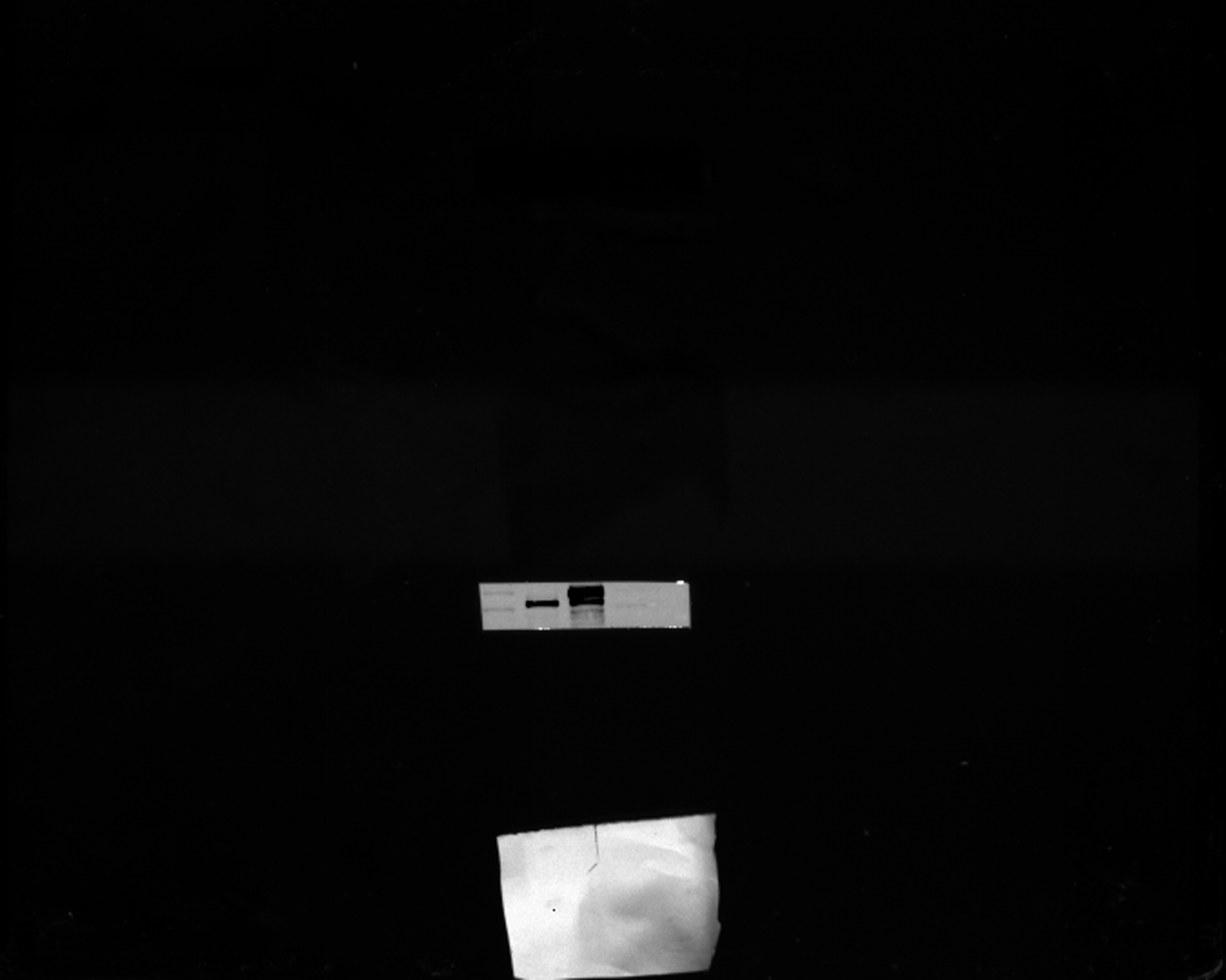


3G MKN45 GAPDH


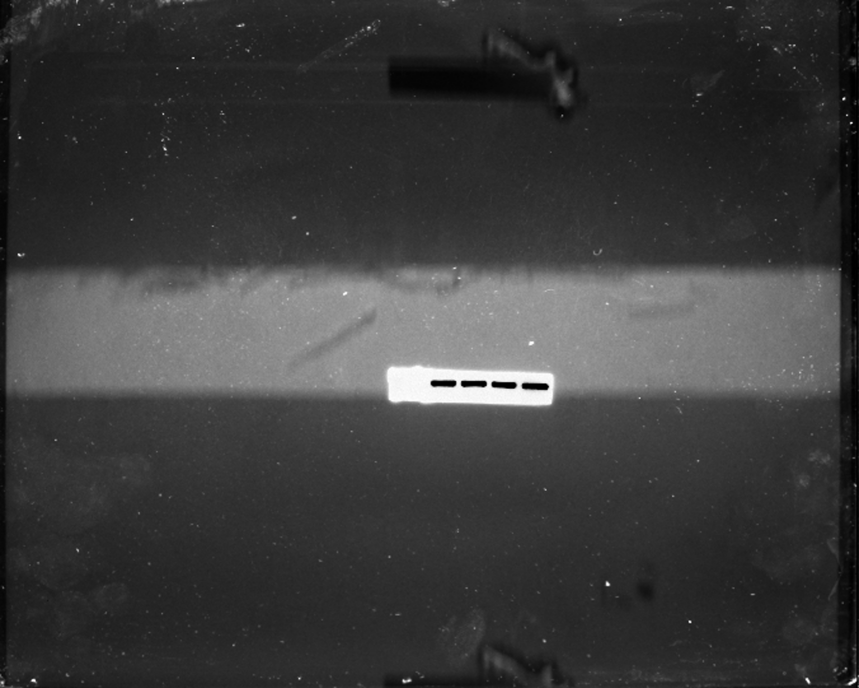

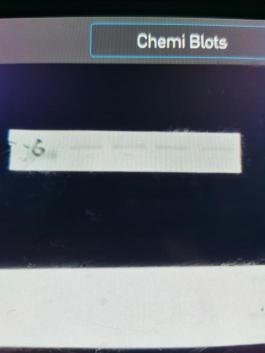


3G MKN45 P62


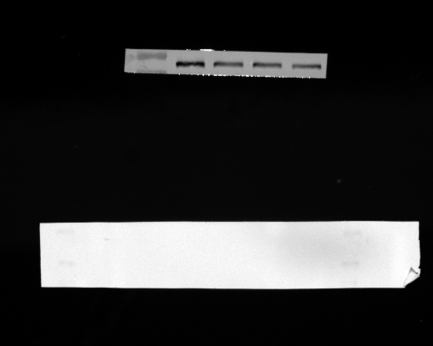


3G MKN45 UBE4B


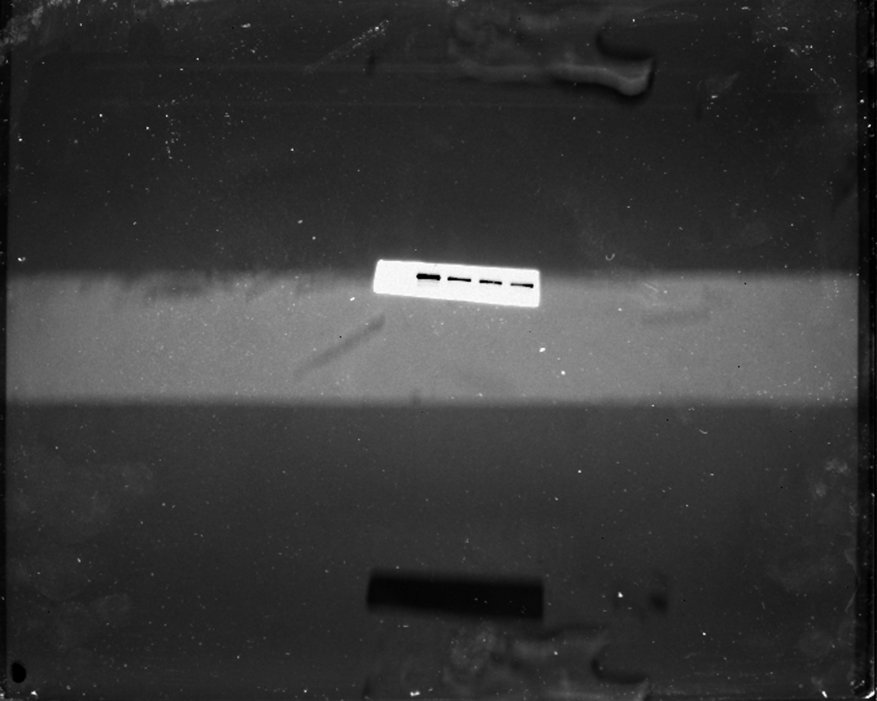

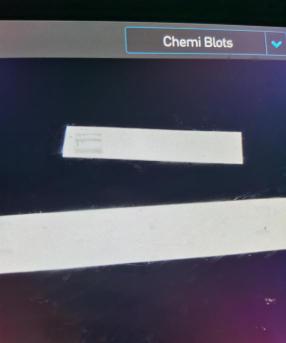


3G MKN45 LC3


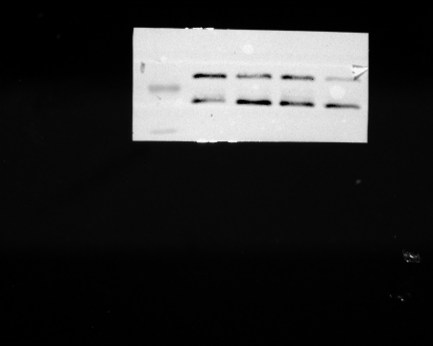


3G AGS GAPDH


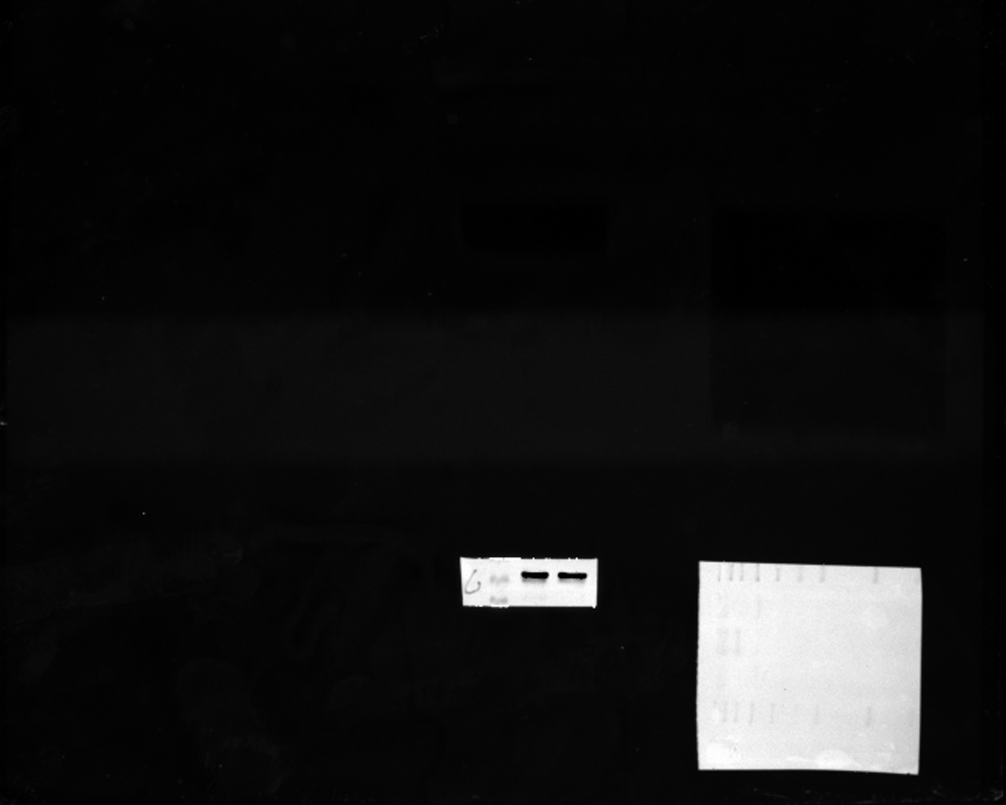


3G AGS P62


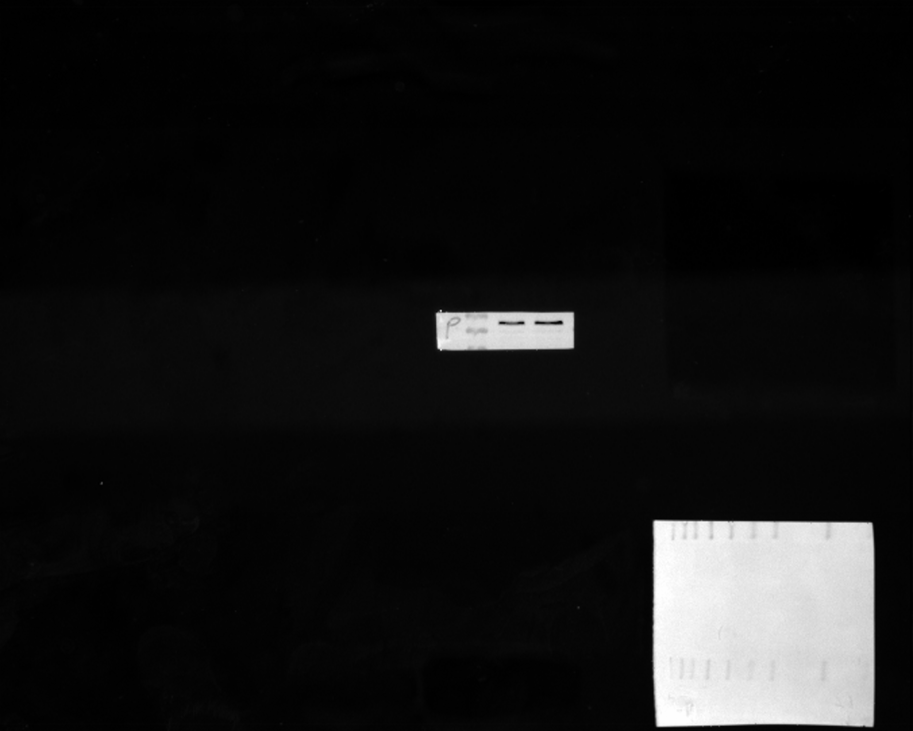


3G AGS UBE4B


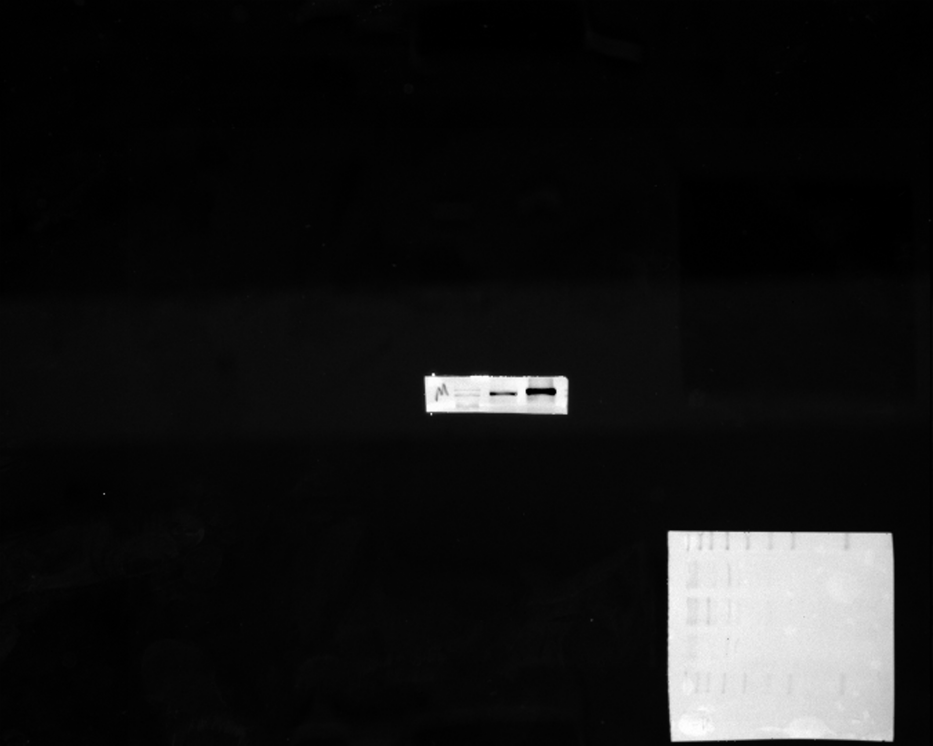


3G AGS LC3


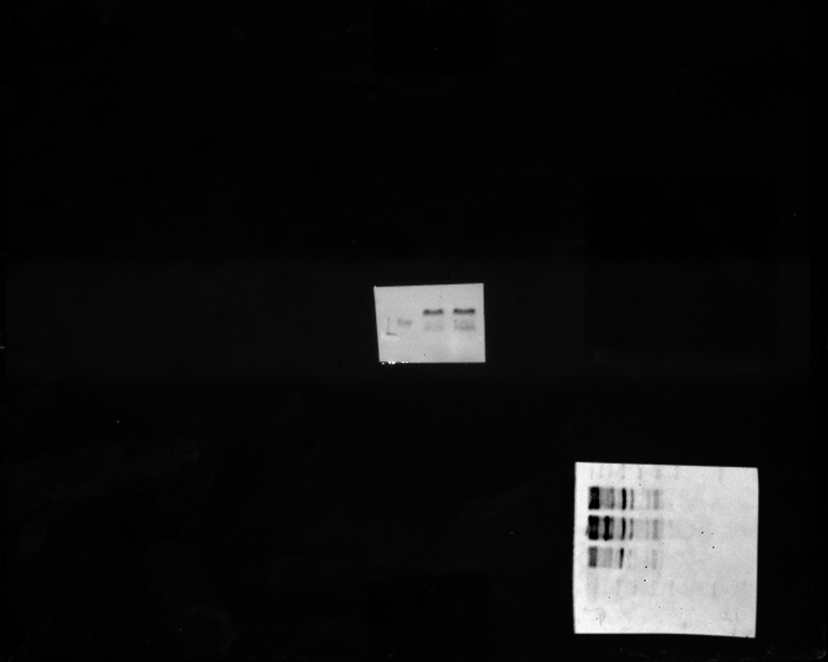


Fig 4

4B GAPDH


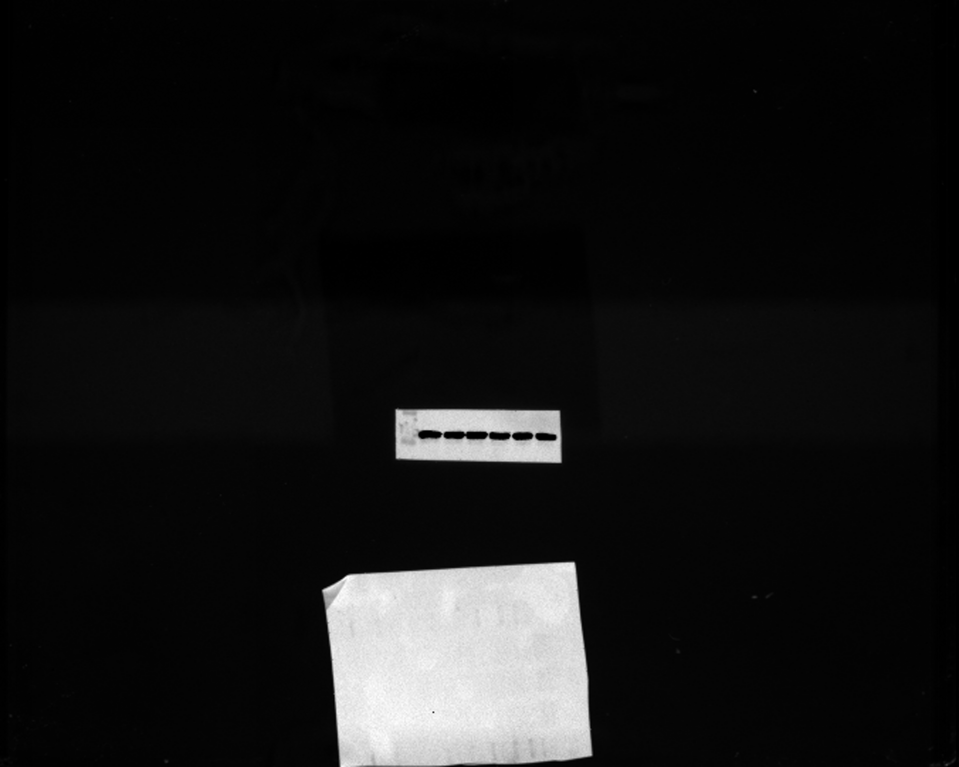


4B UBE4B


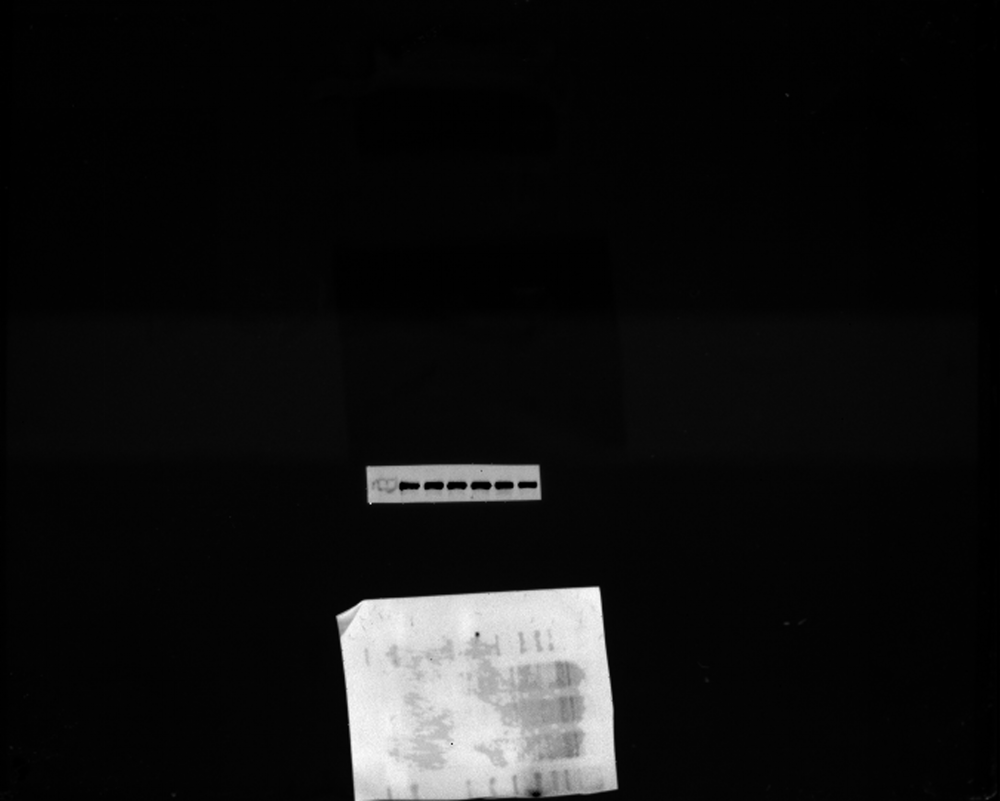


4B FAT4


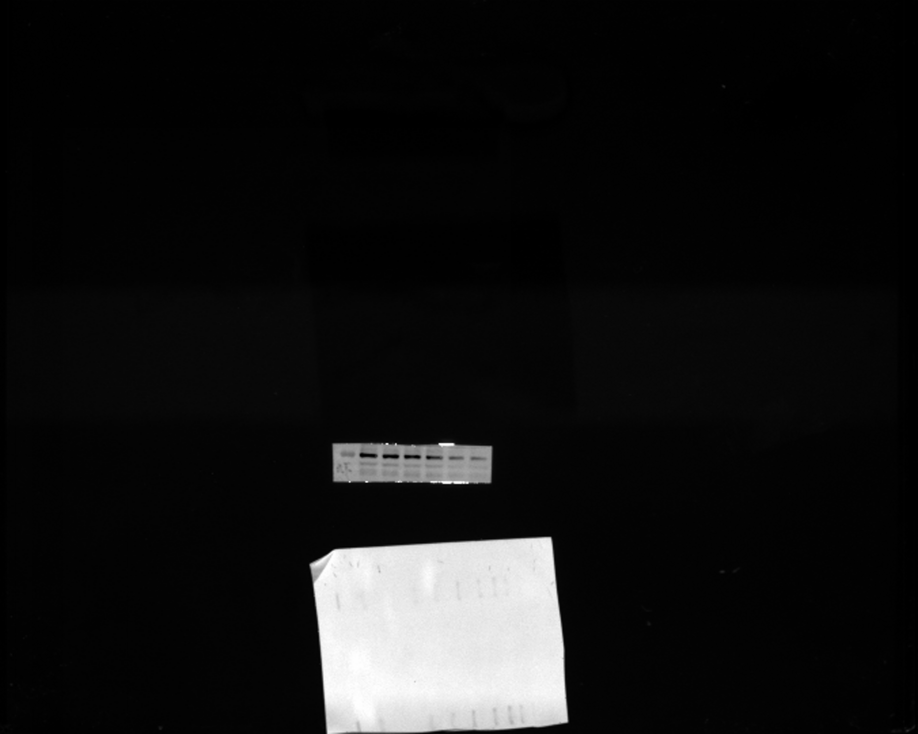


4B siUBE4B-1 GAPDH


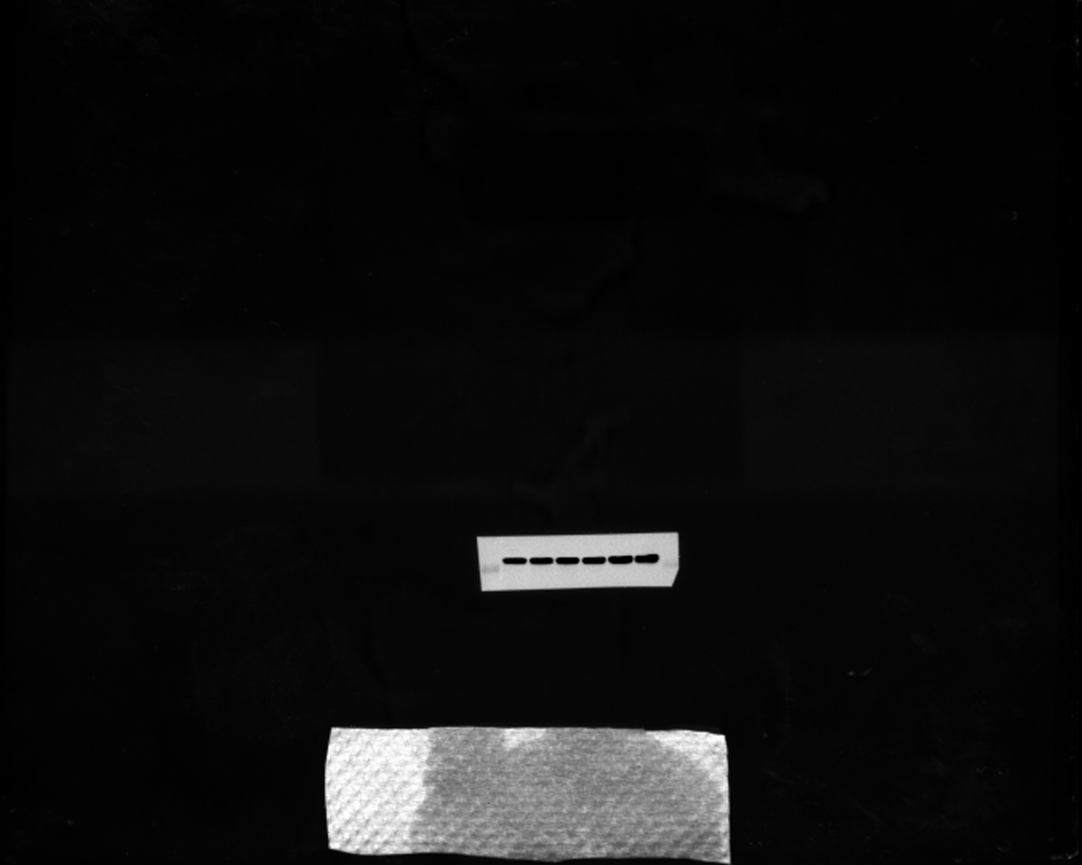


4B siUBE4B-1 UBE4B


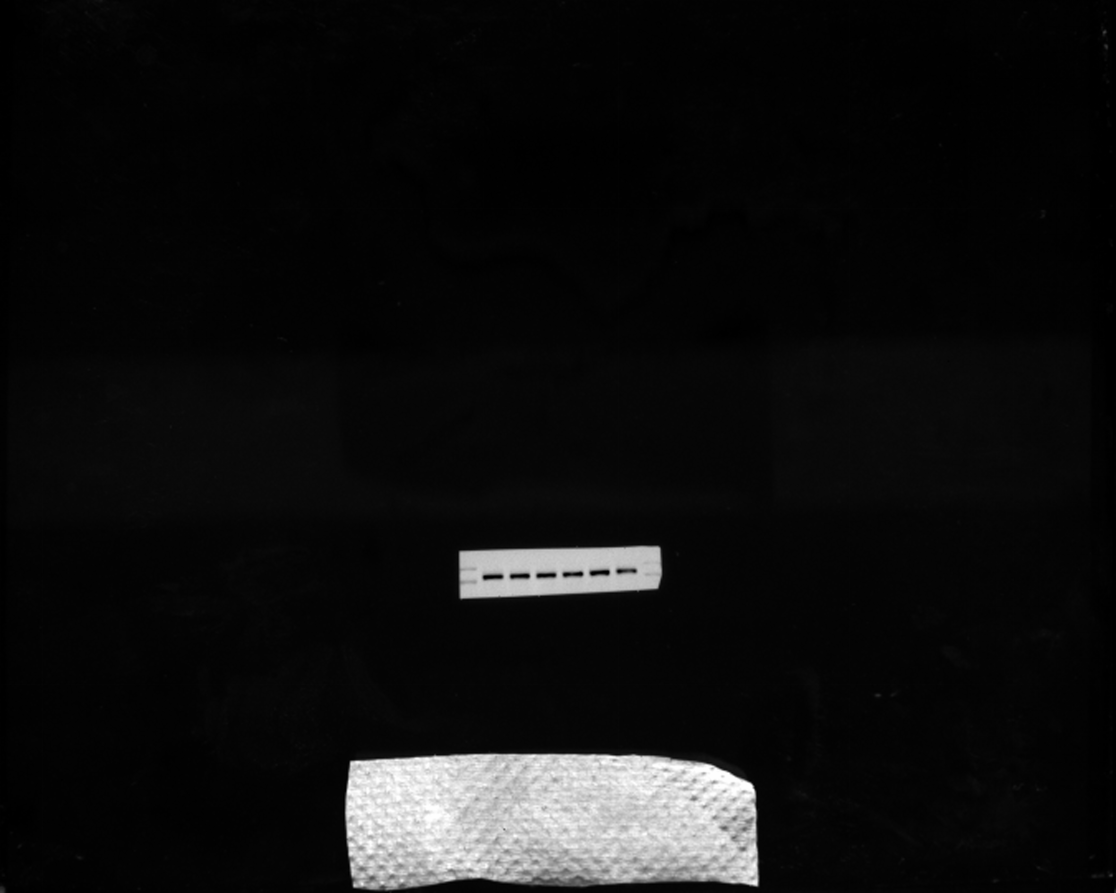


4B siUBE4B-1 FAT4


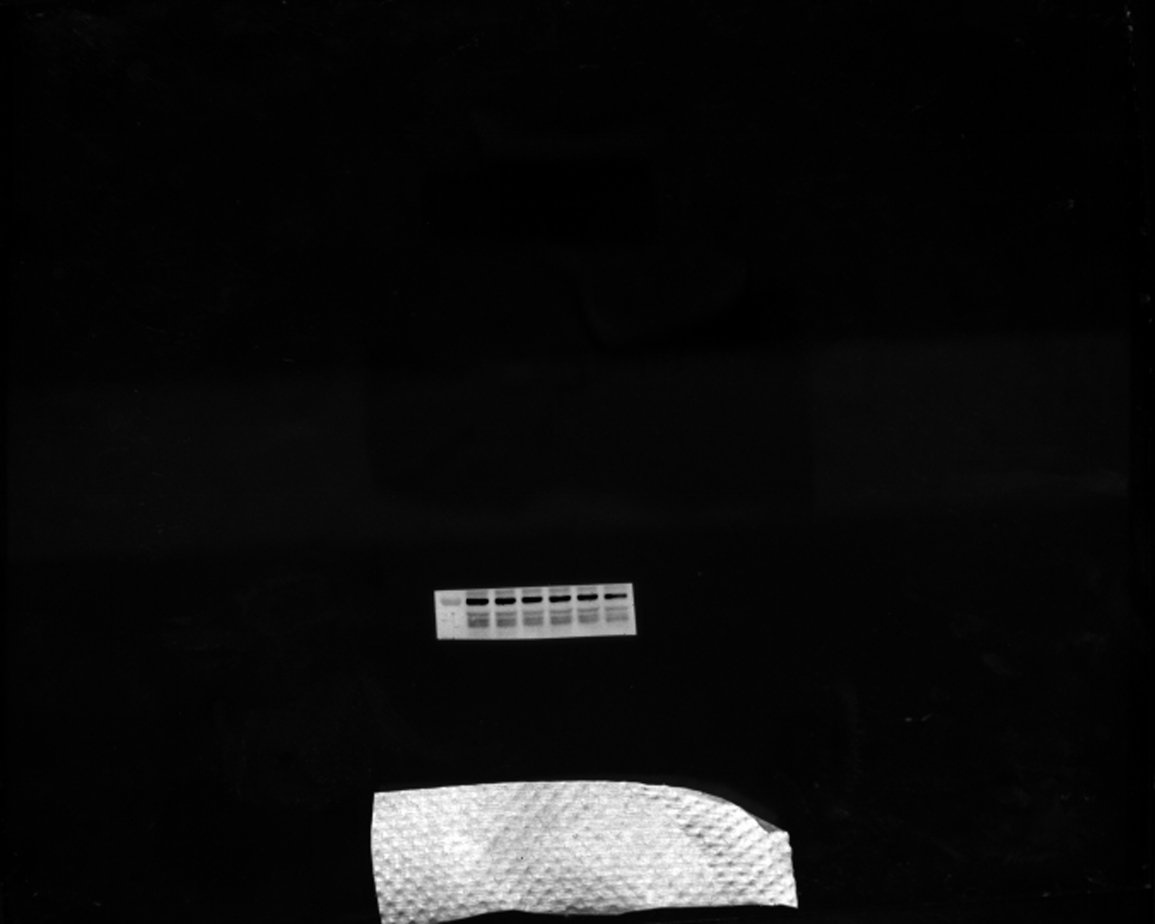


4C GAPDH


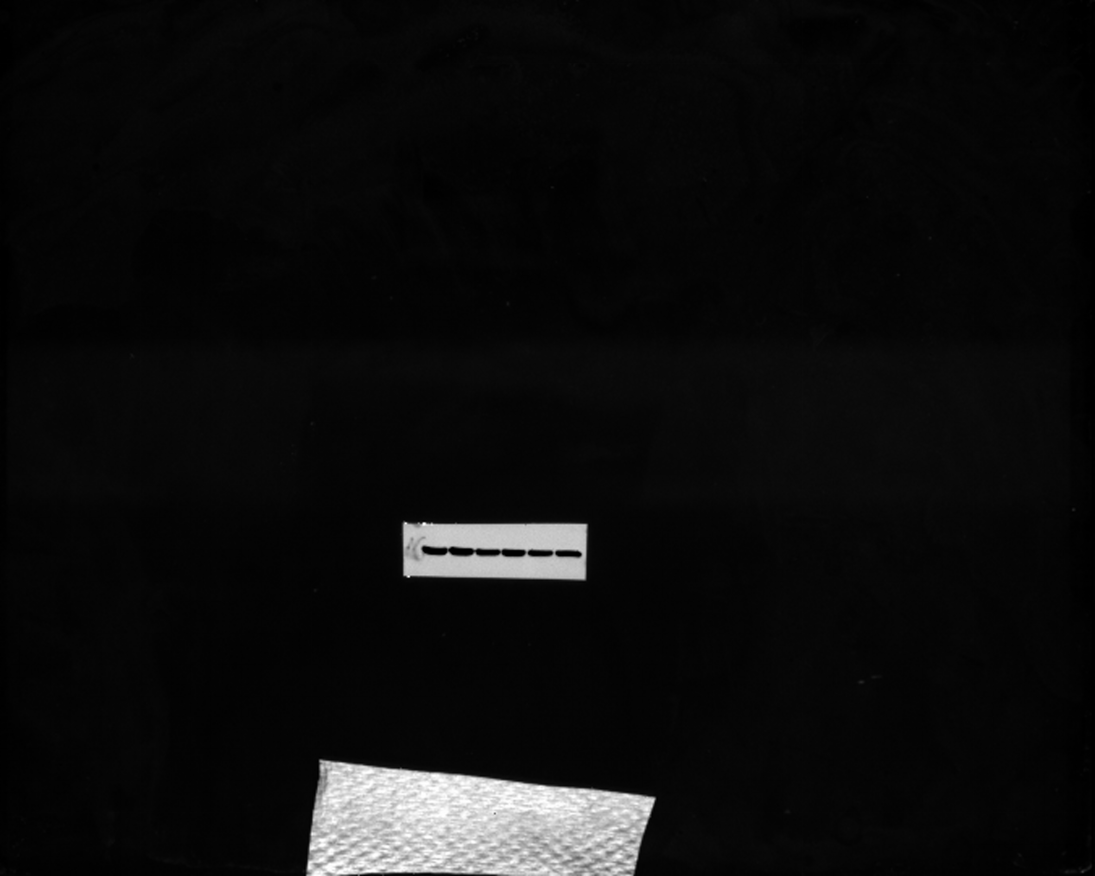


4C UBE4B


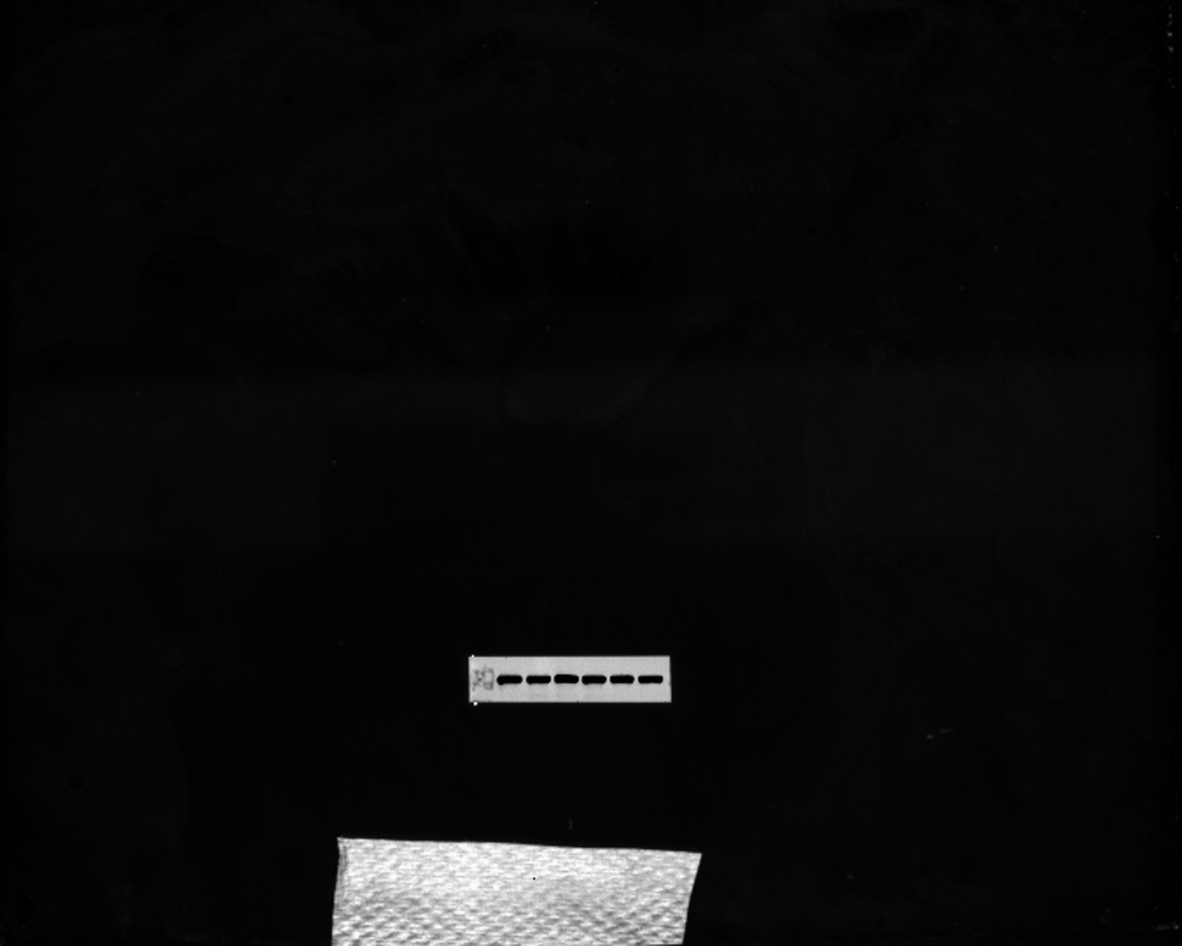


4C FAT4


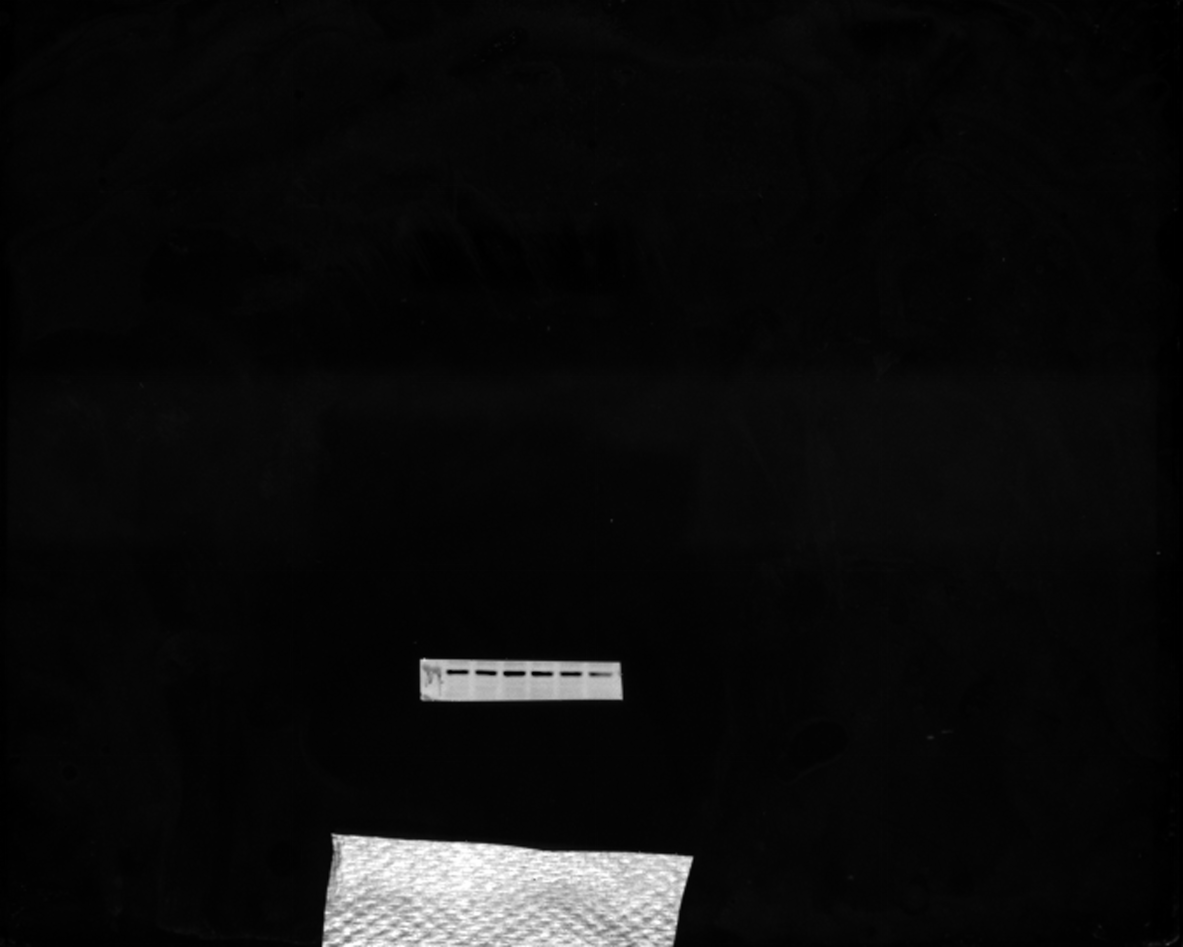


4C siUBE4B-2 GAPDH


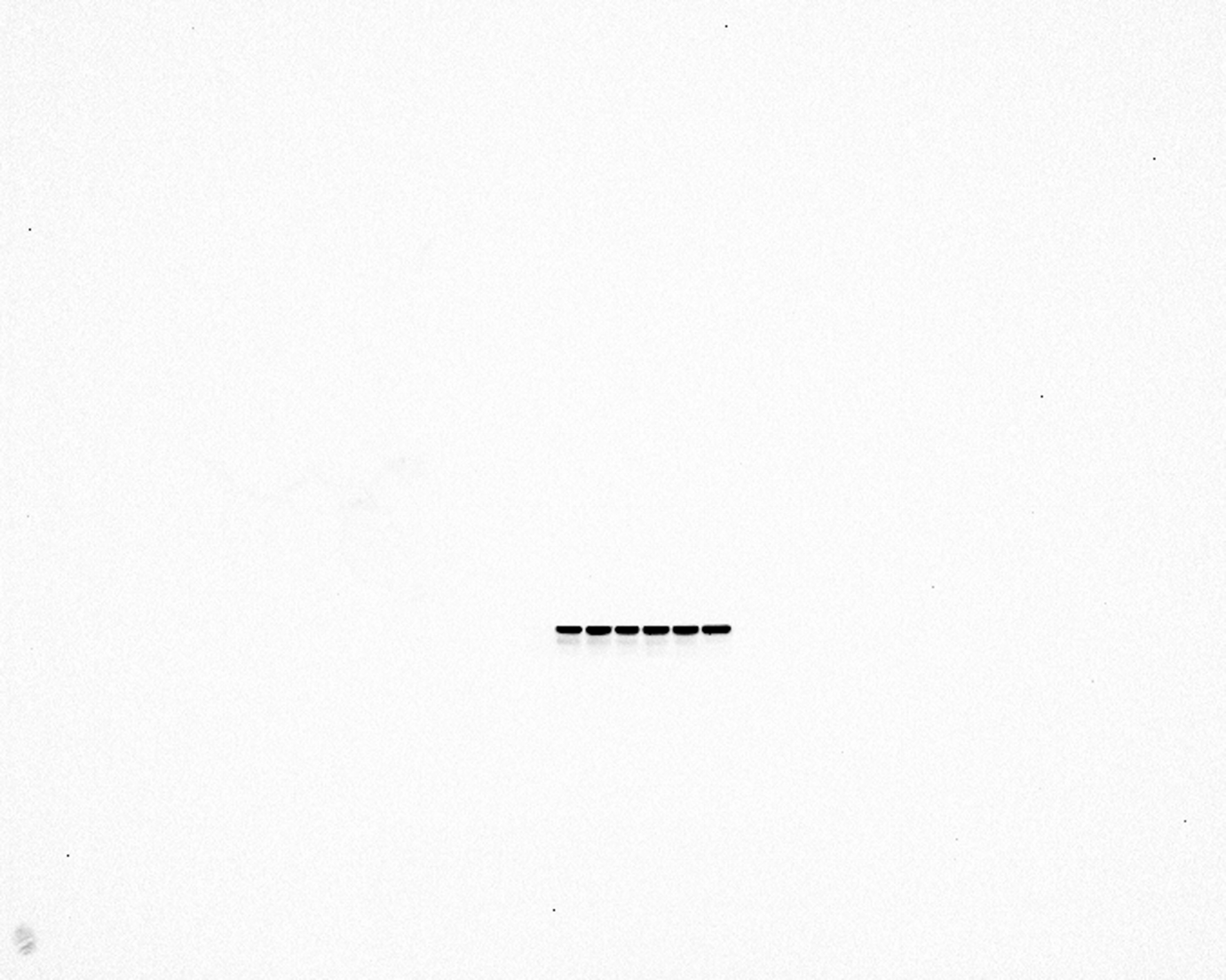

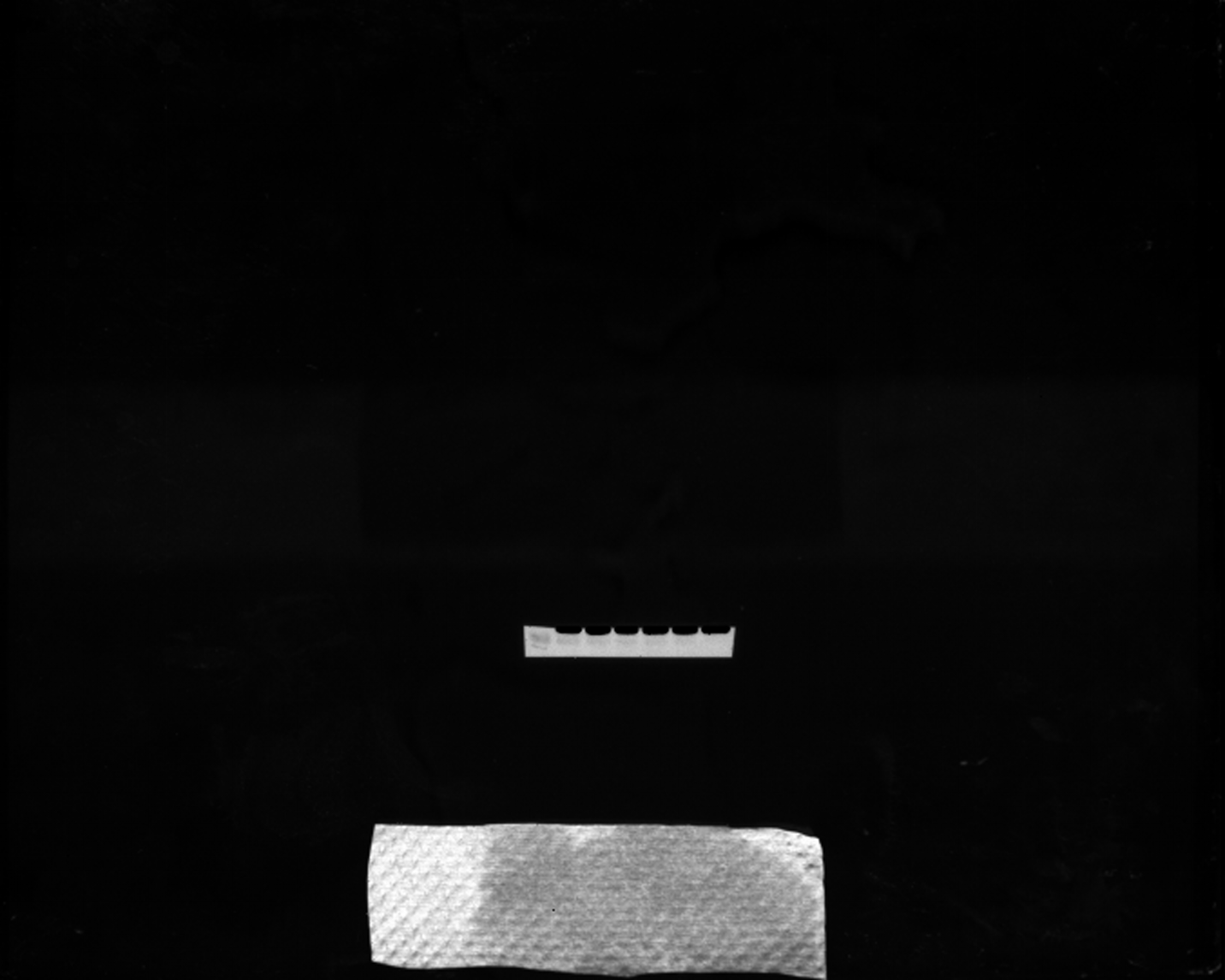


4C siUBE4B-2 UBE4B


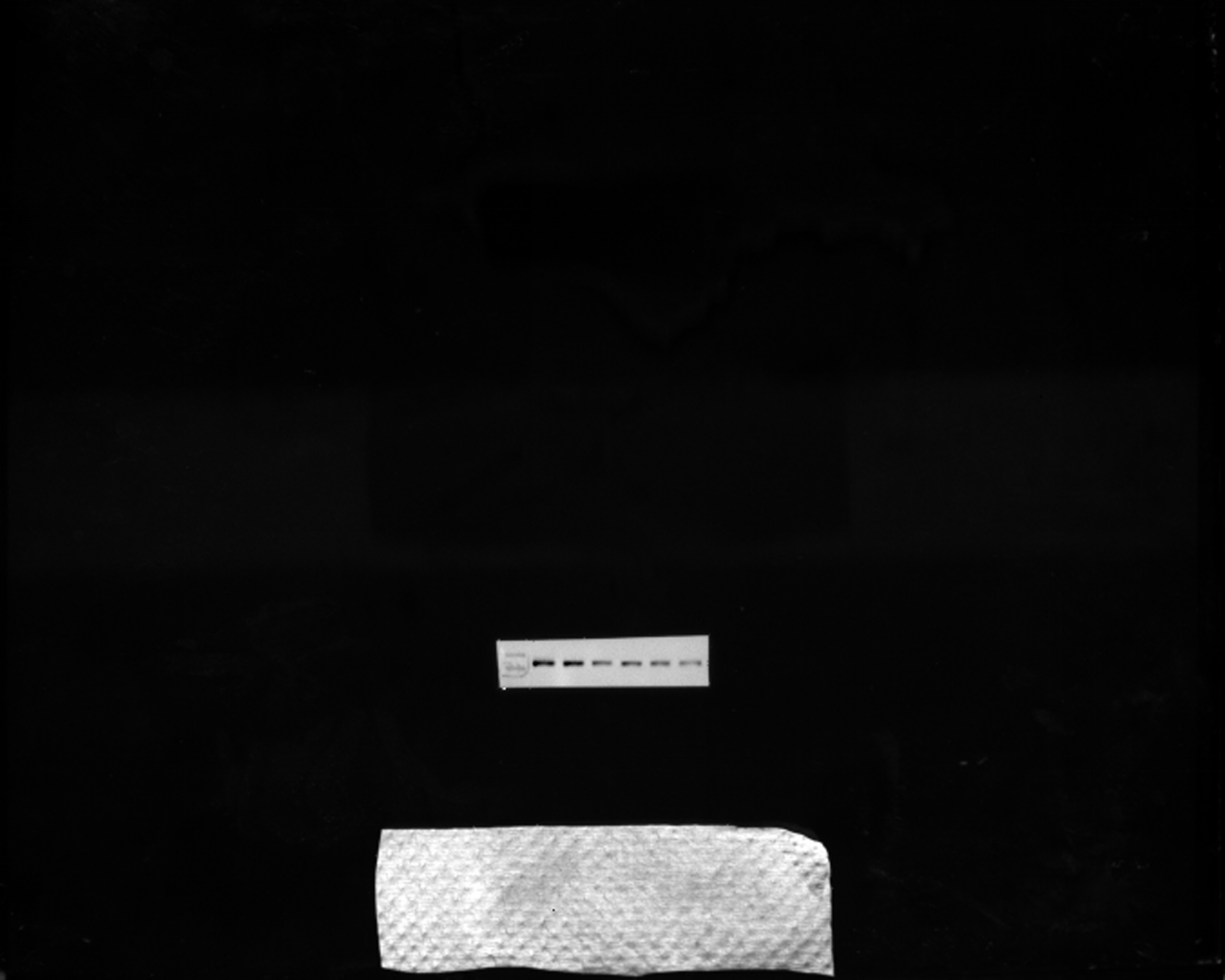


4C siUBE4B-2 FAT4


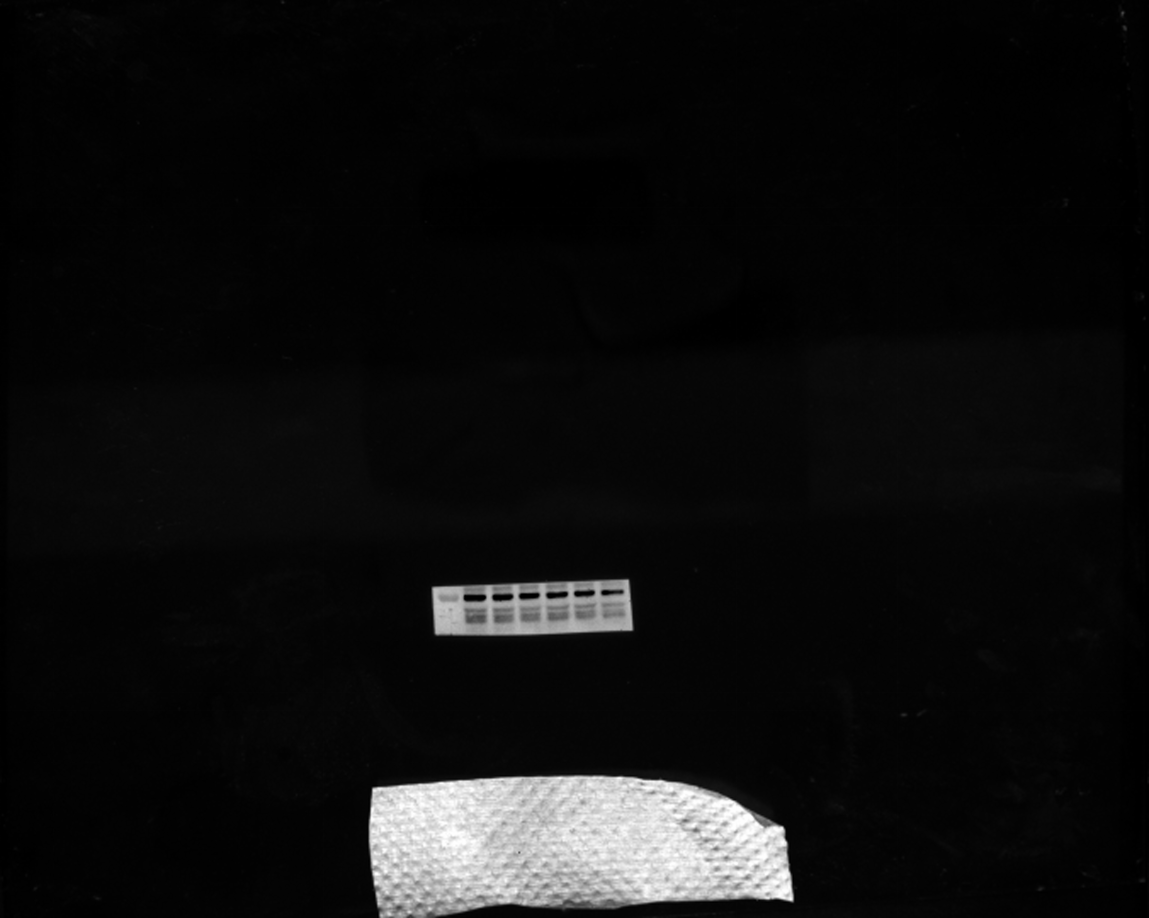


4D GAPDH


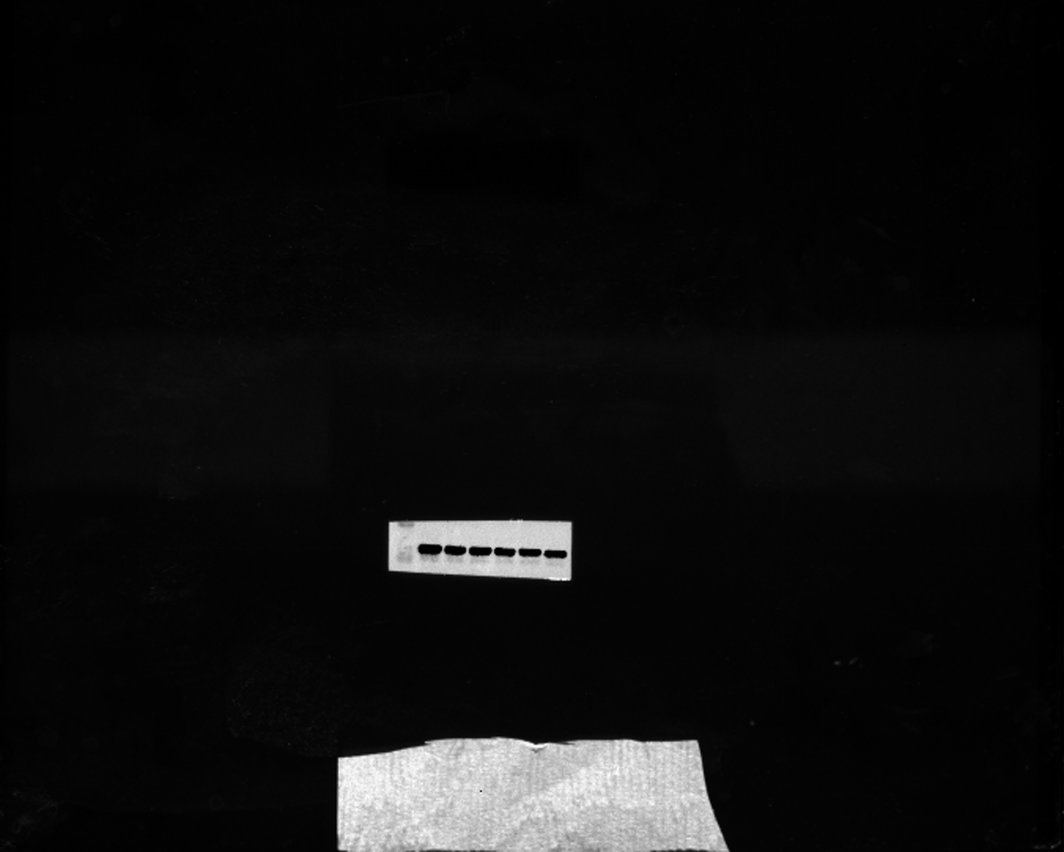


4D UBE4B


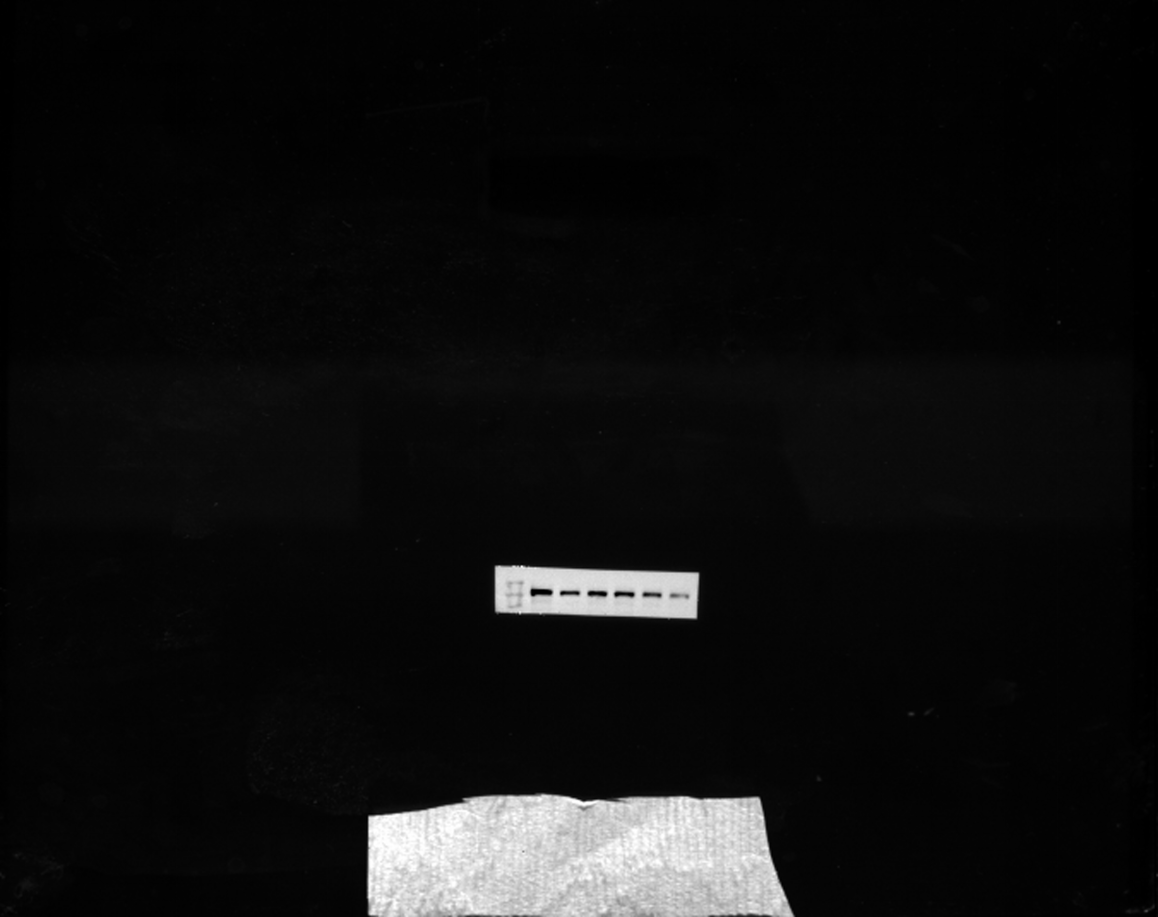


4D FAT4


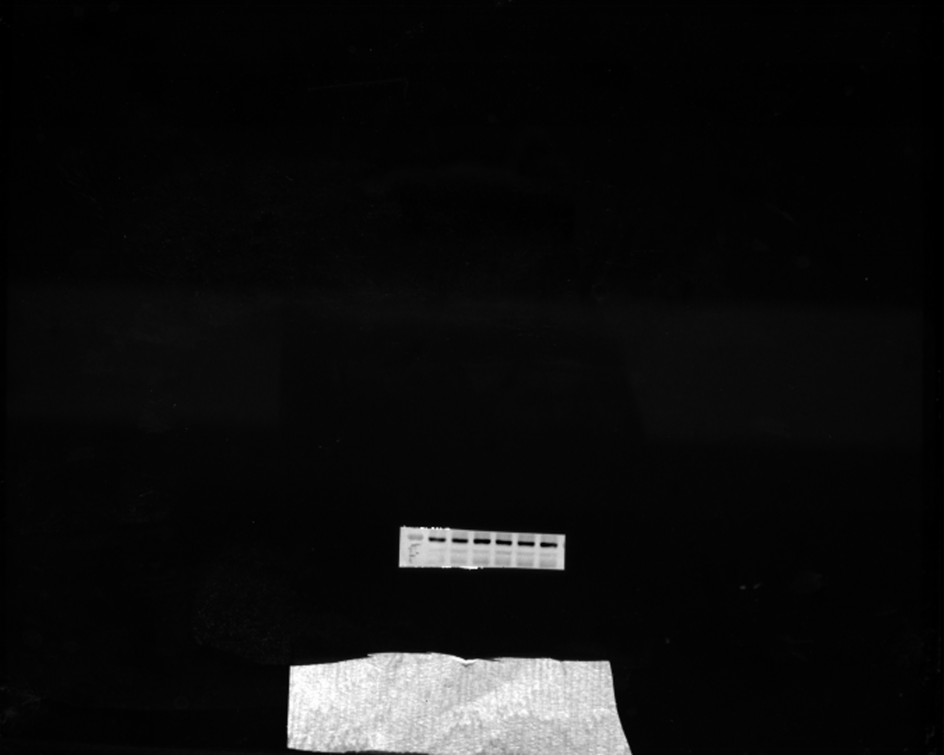


4E MYC


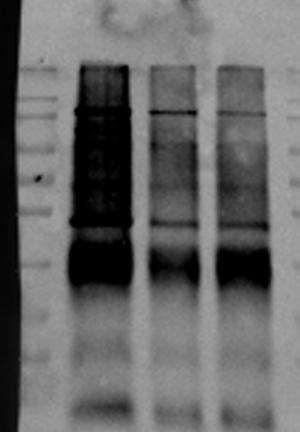


4E IP：FAT4


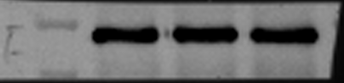


4E input：FAT4


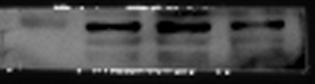


4E UBE4B


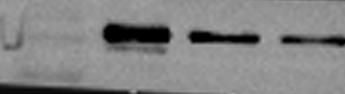


4E GAPDH


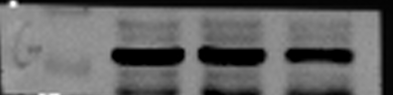


4F MYC


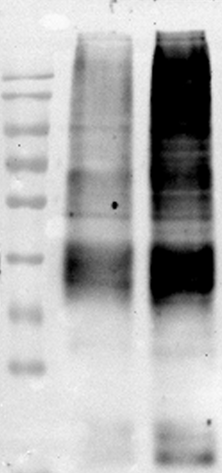


4F IP：FAT4


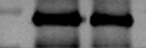


4F input：FAT4


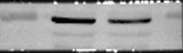


4F UBE4B


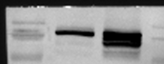


4F GAPDH


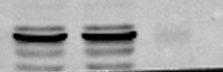


Fig 5

5C Input GAPDH


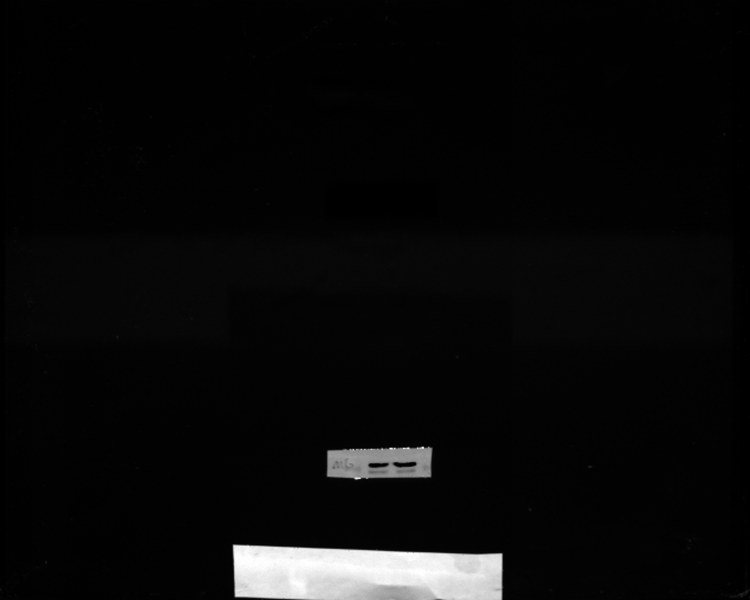


5C Input UBE4B


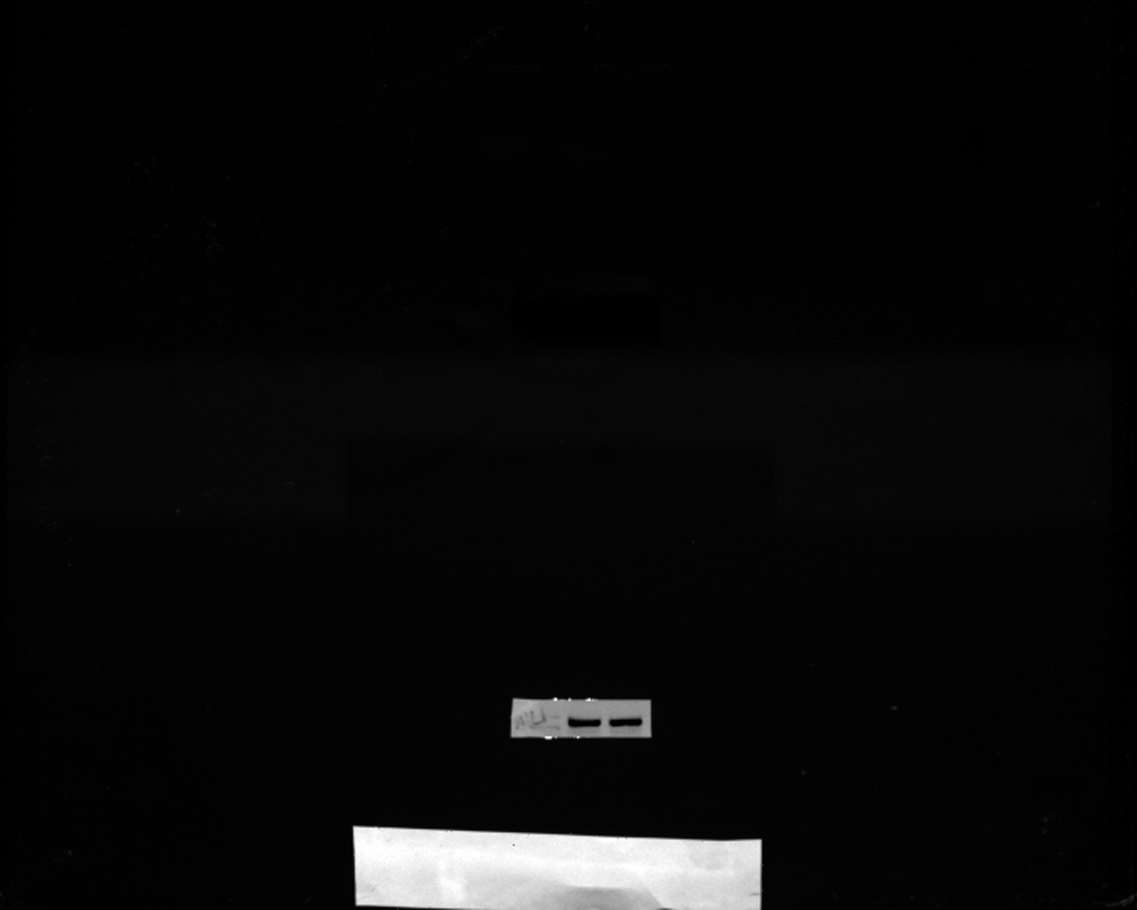


5C Input FAT4


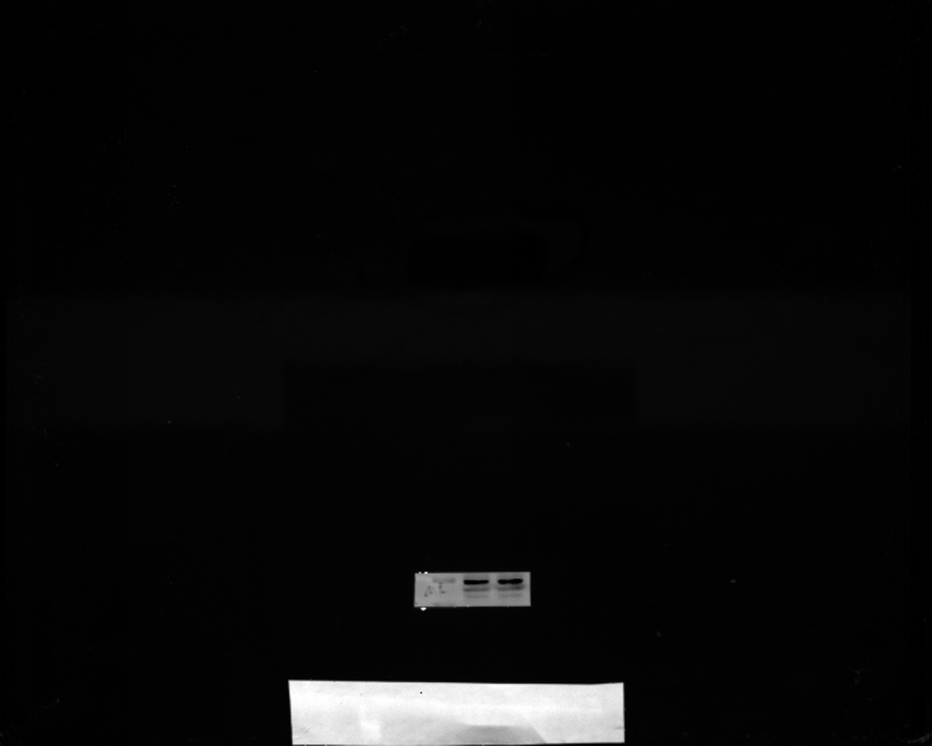


5C IP-UBE4B UBE4B


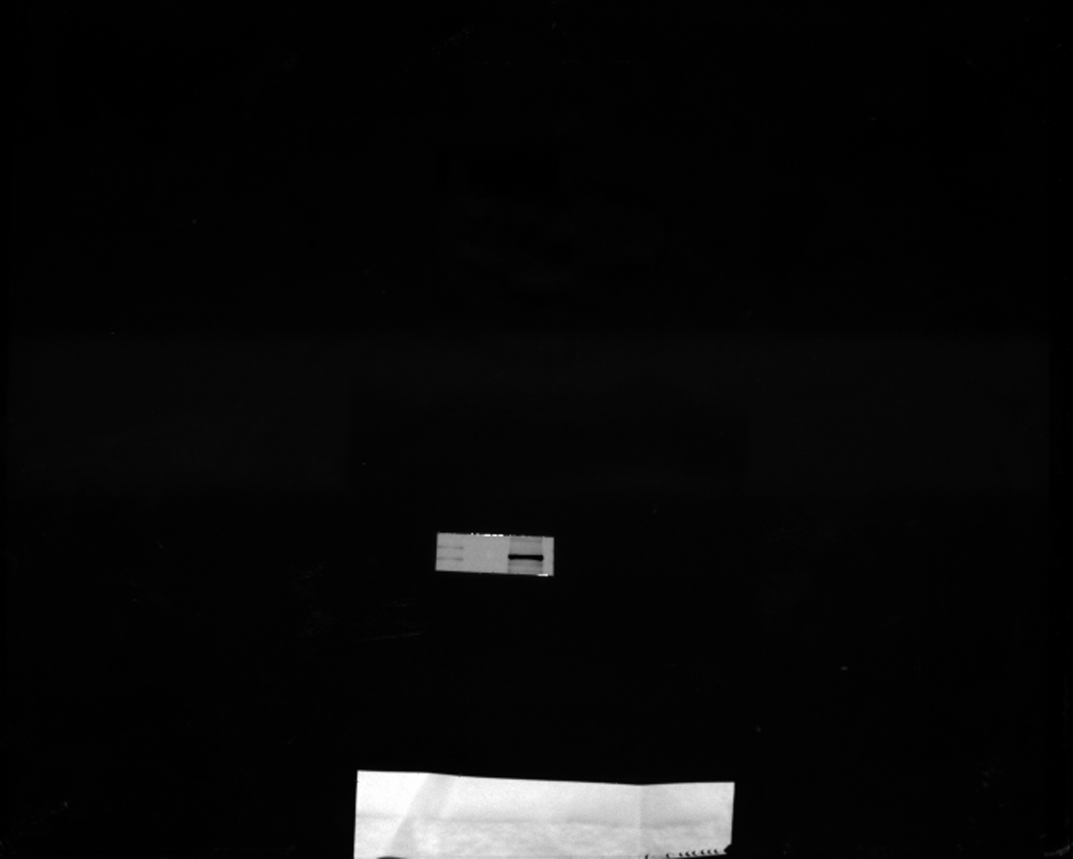


5C IP-UBE4B FAT4


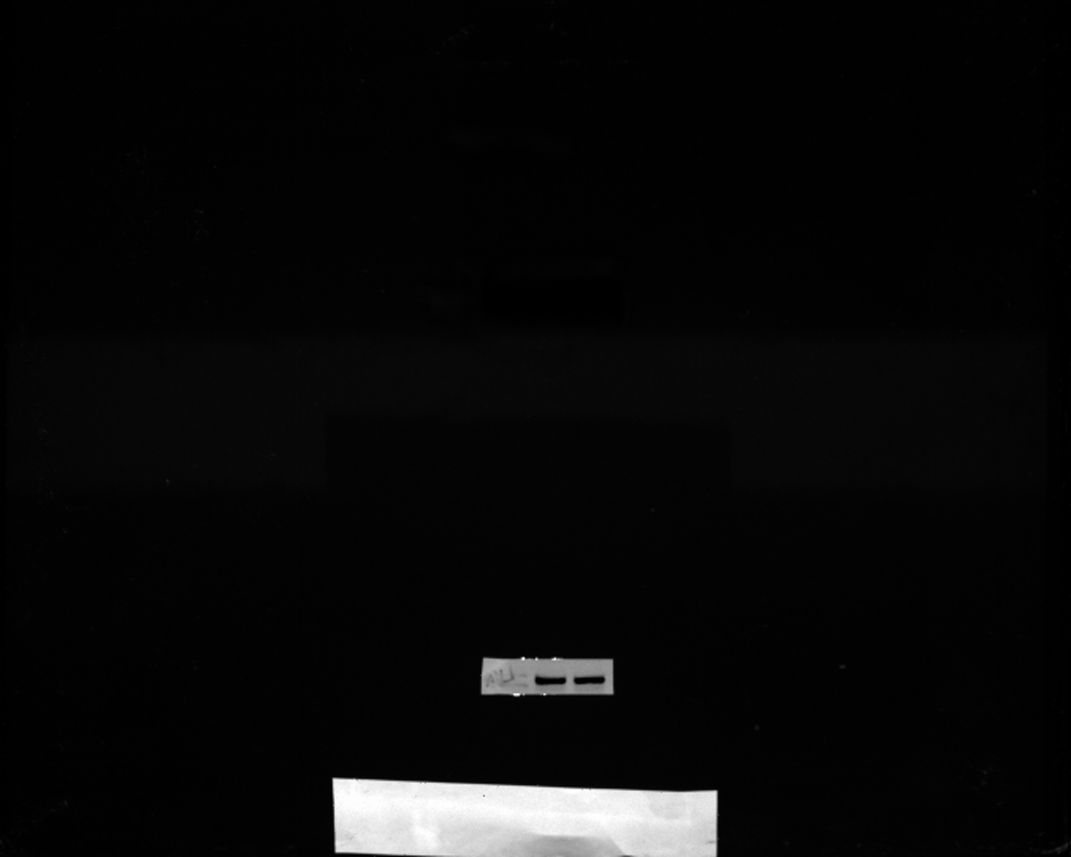


5C IP-FAT4 UBE4B


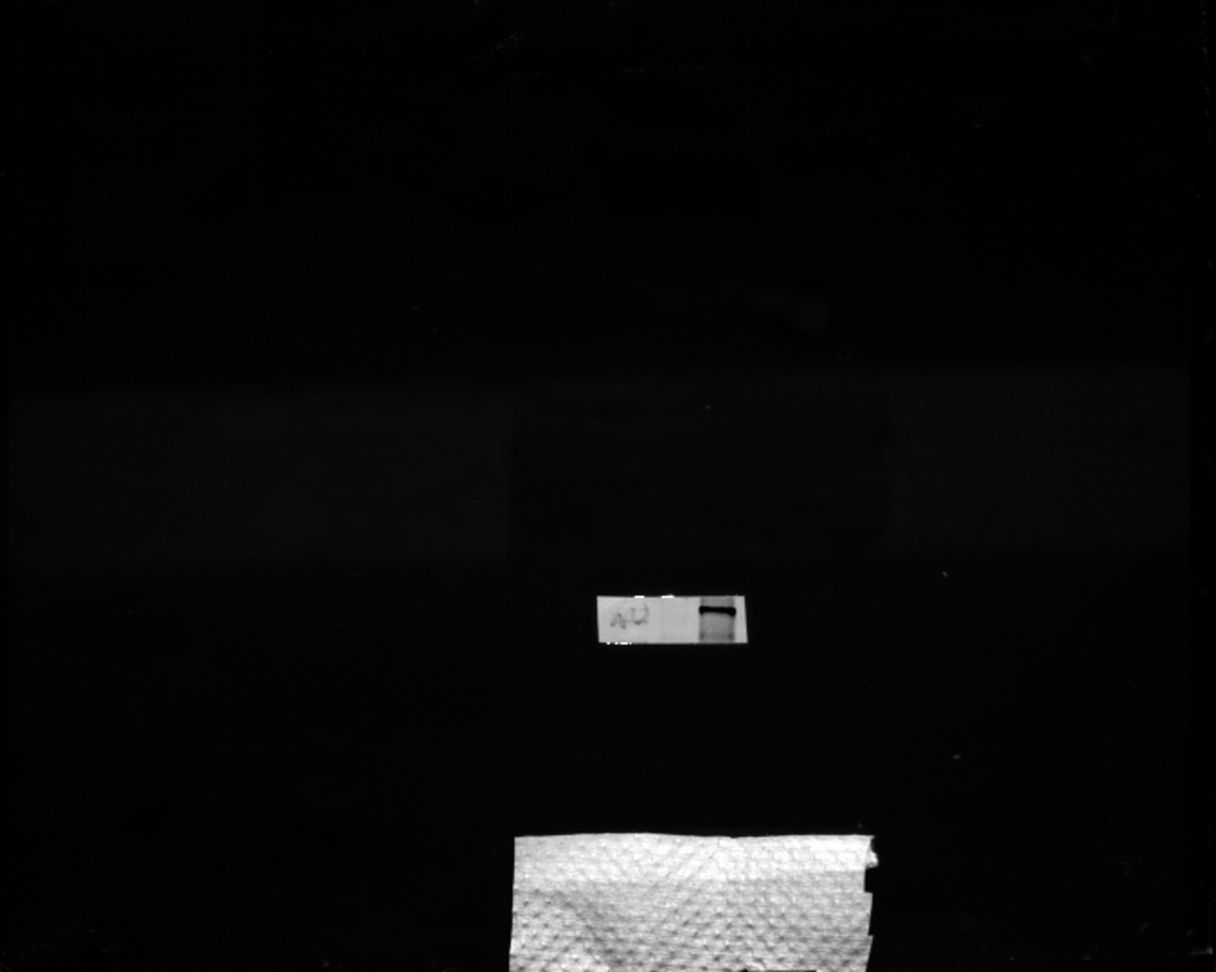


5C IP-FAT4 FAT4


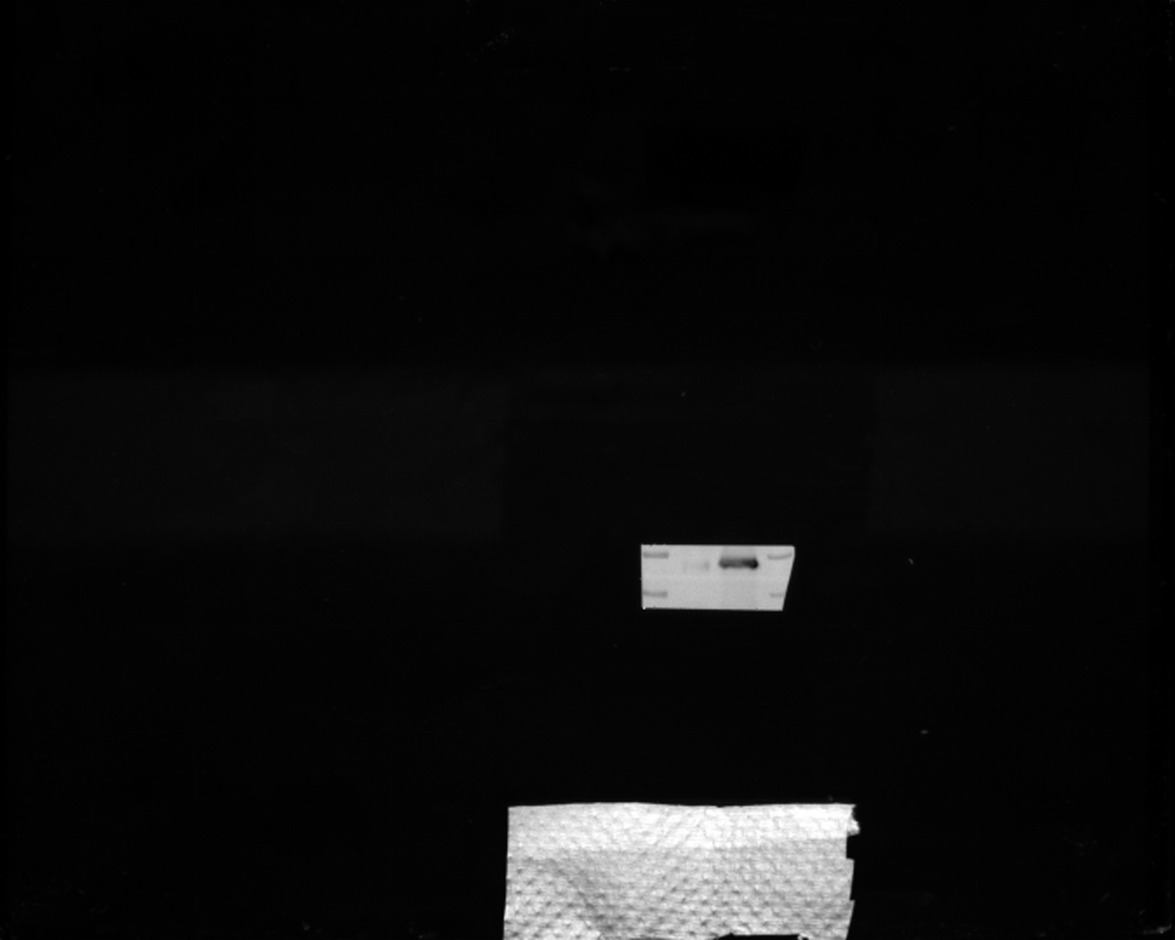


5D Input GAPDH


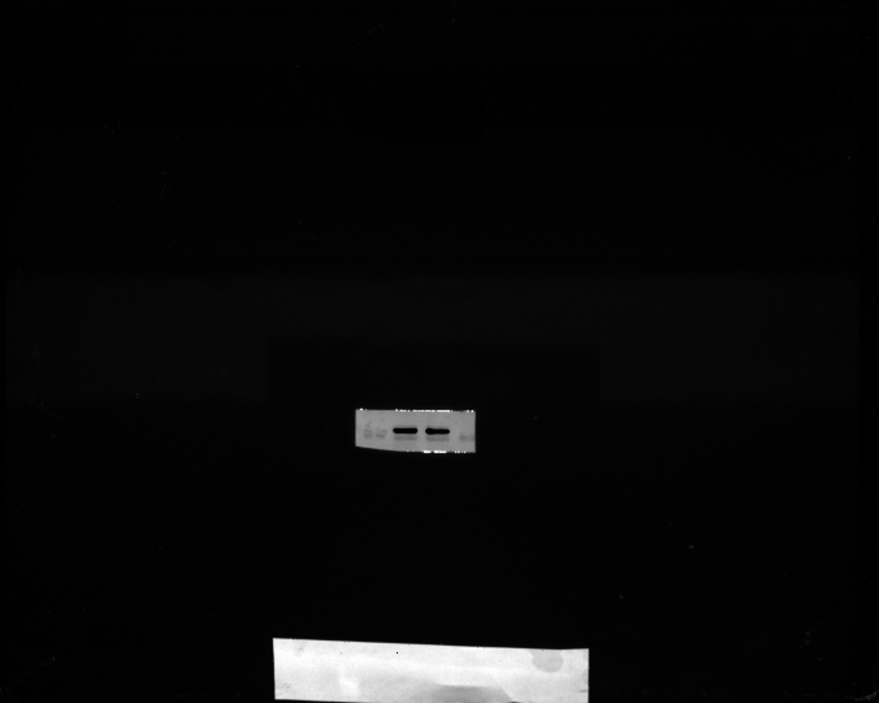


5D Input UBE4B


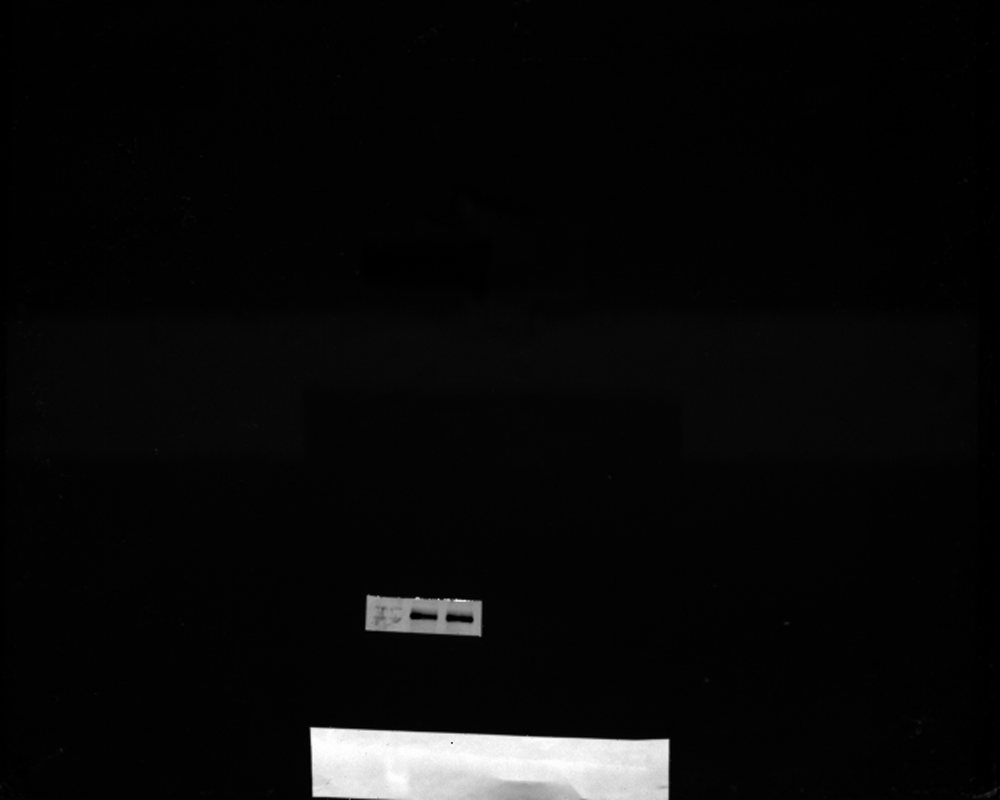


5D Input FAT4


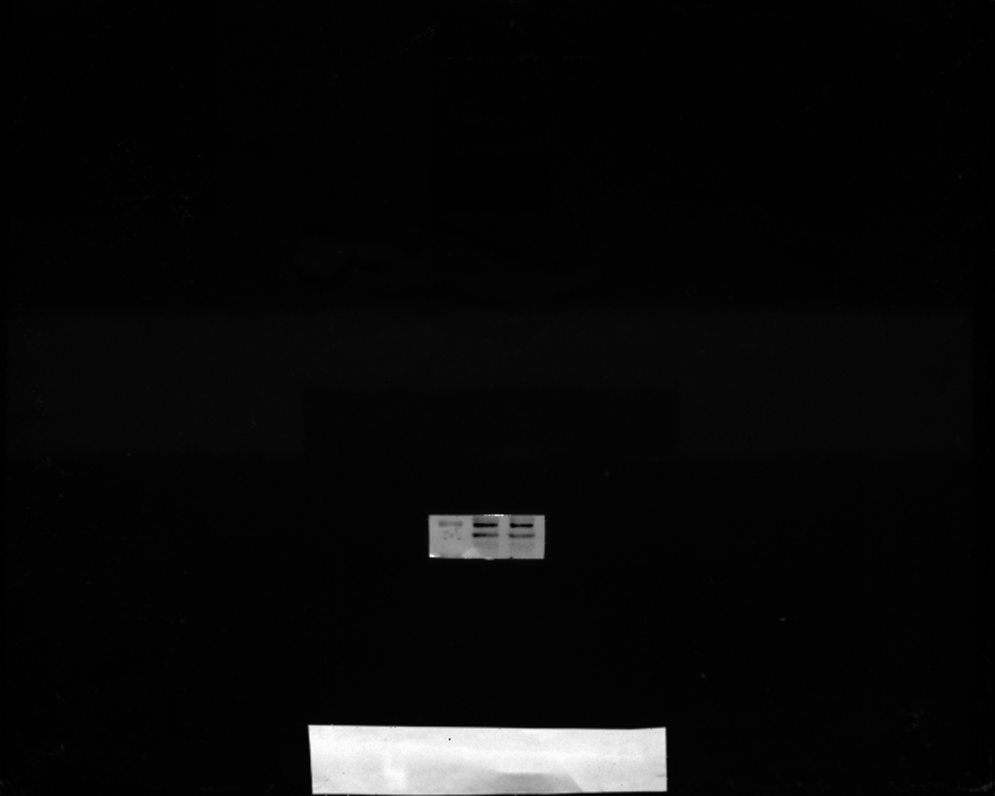


5D IP-UBE4B UBE4B


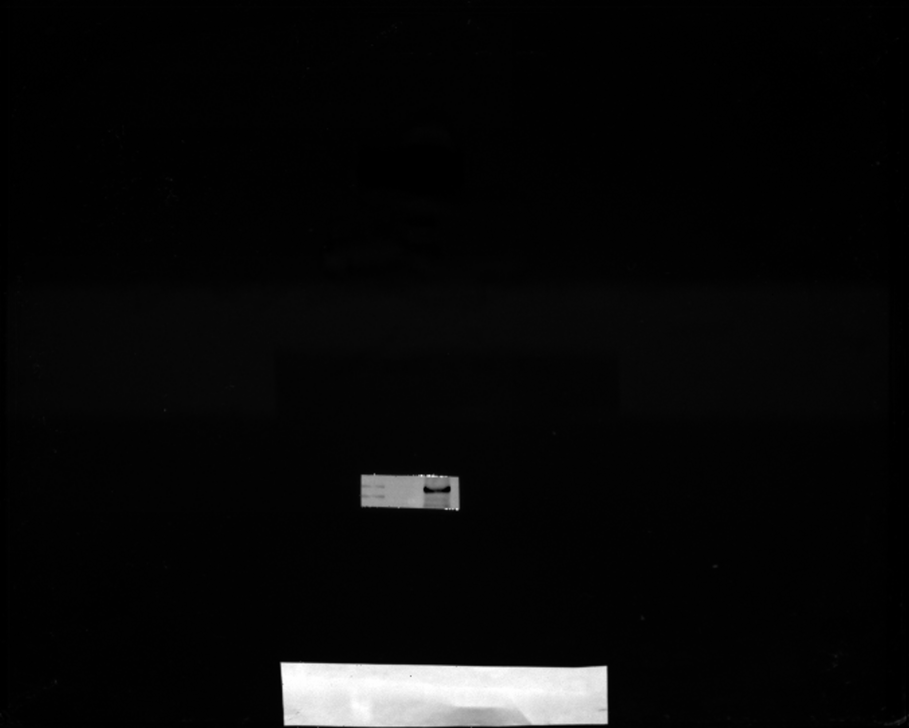


5D IP-UBE4B FAT4


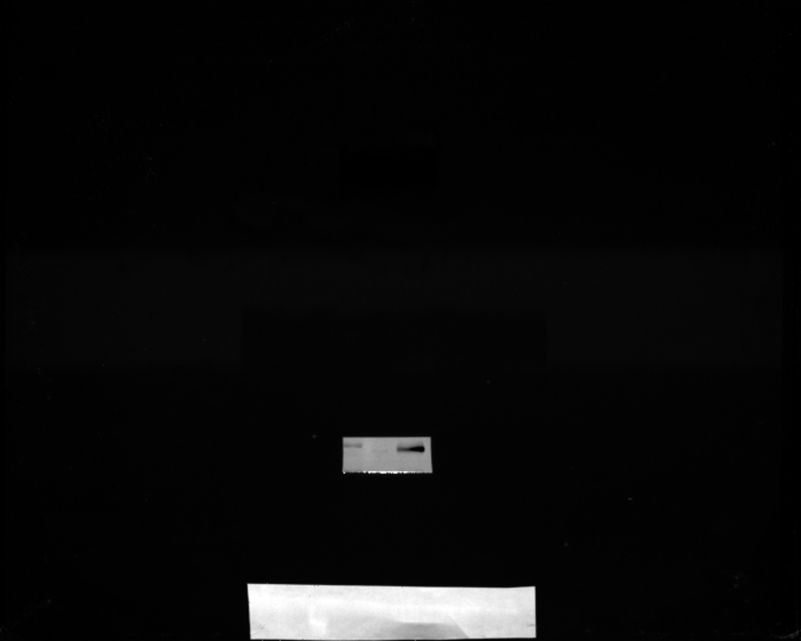


5D IP-FAT4 UBE4B


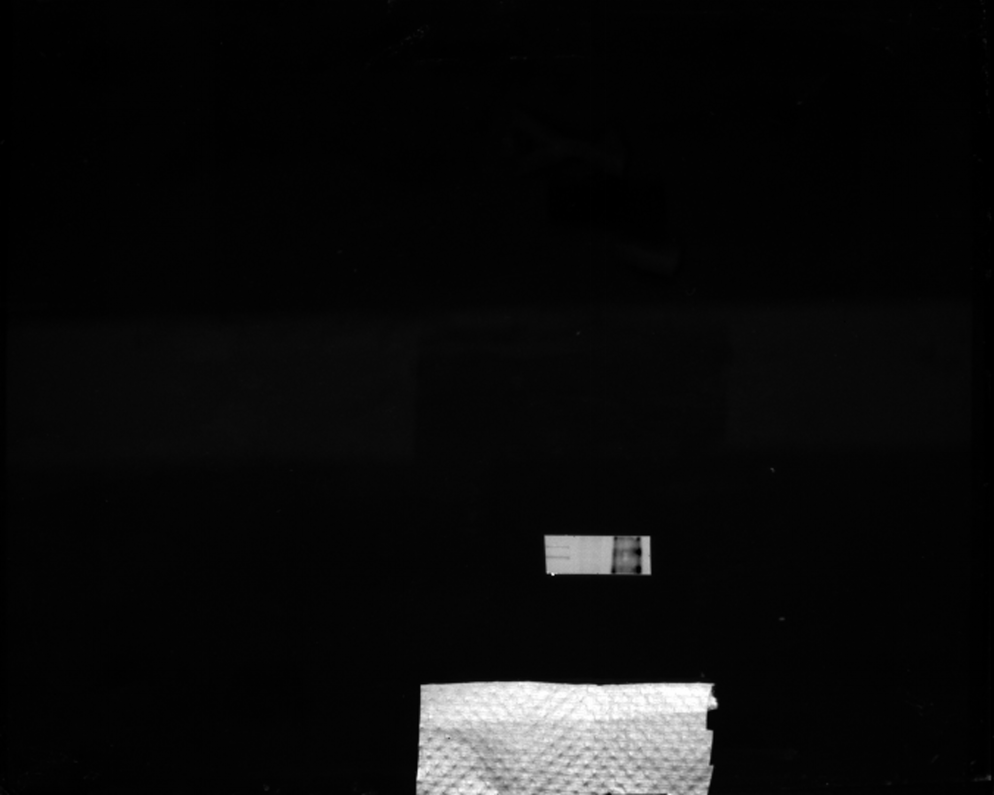


5D IP-FAT4 FAT4


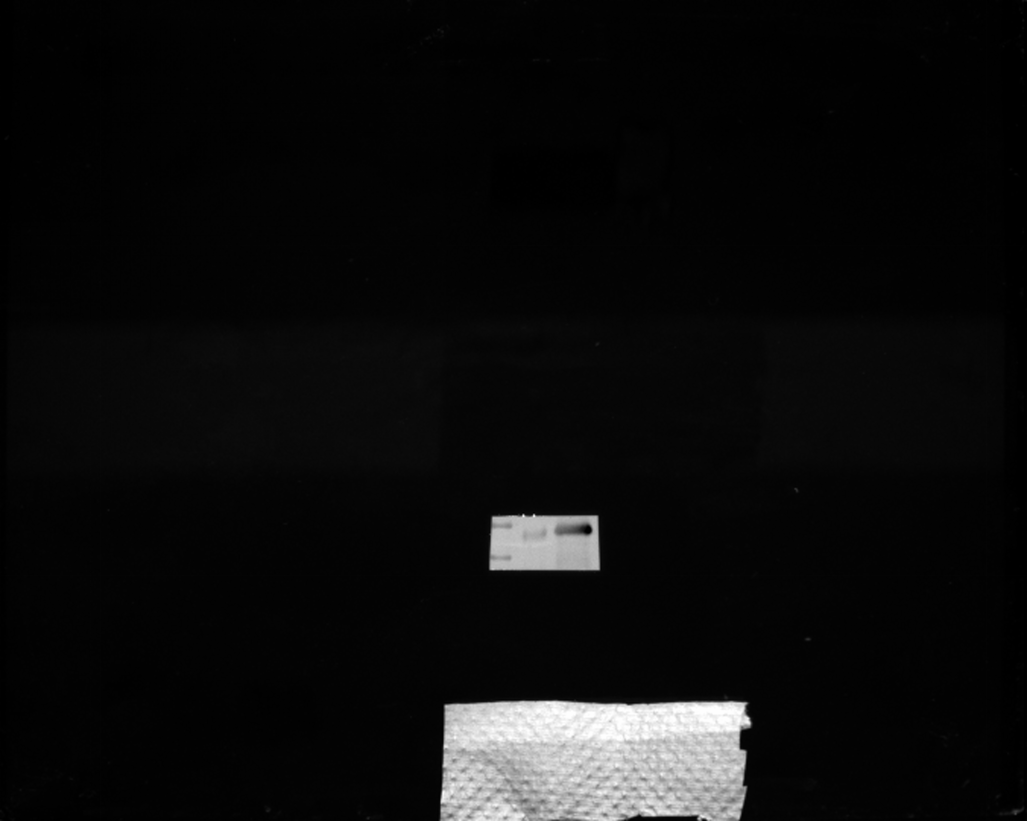


5E Input GAPDH


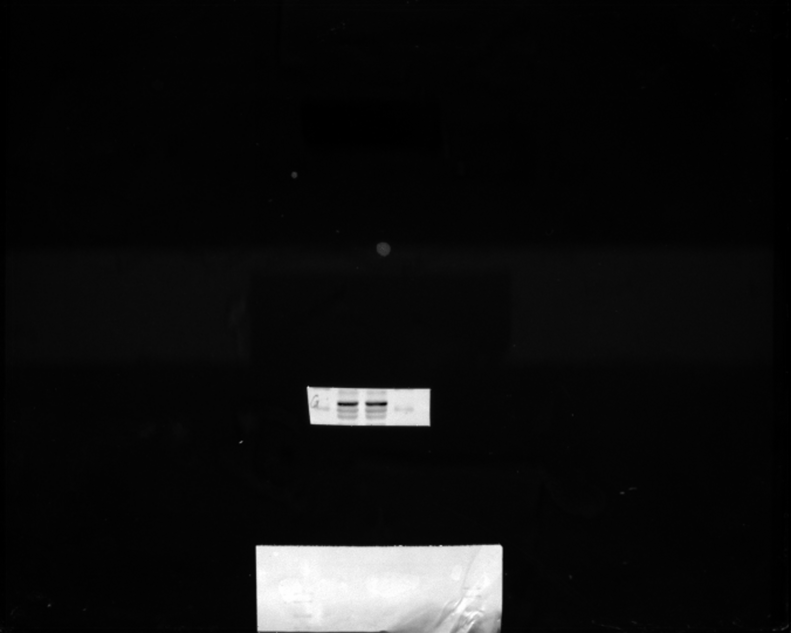


5E Input UBE4B


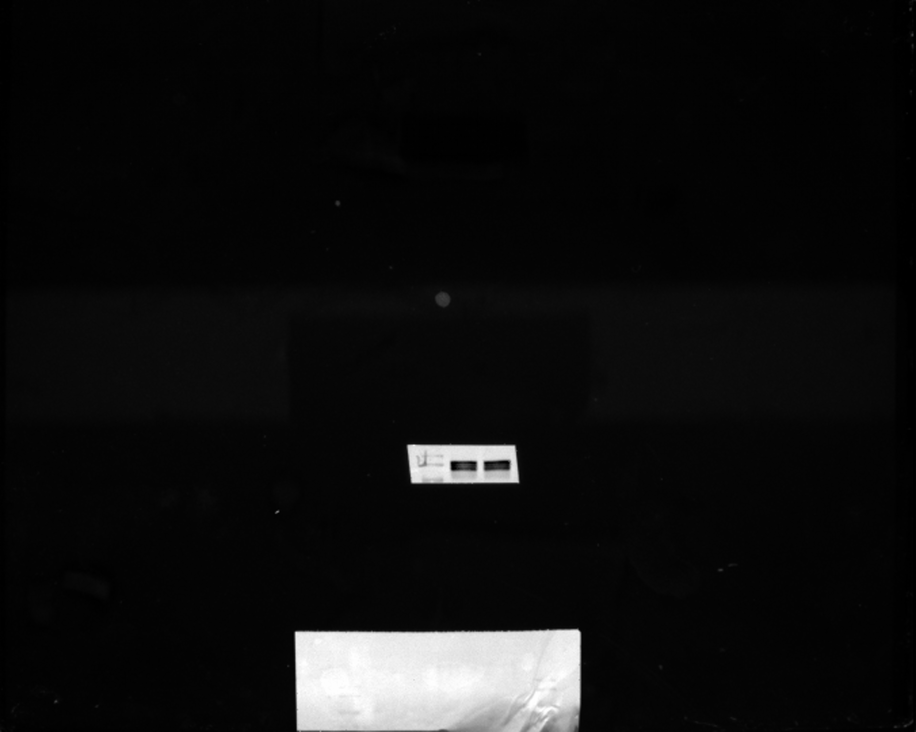


5E Input FAT4


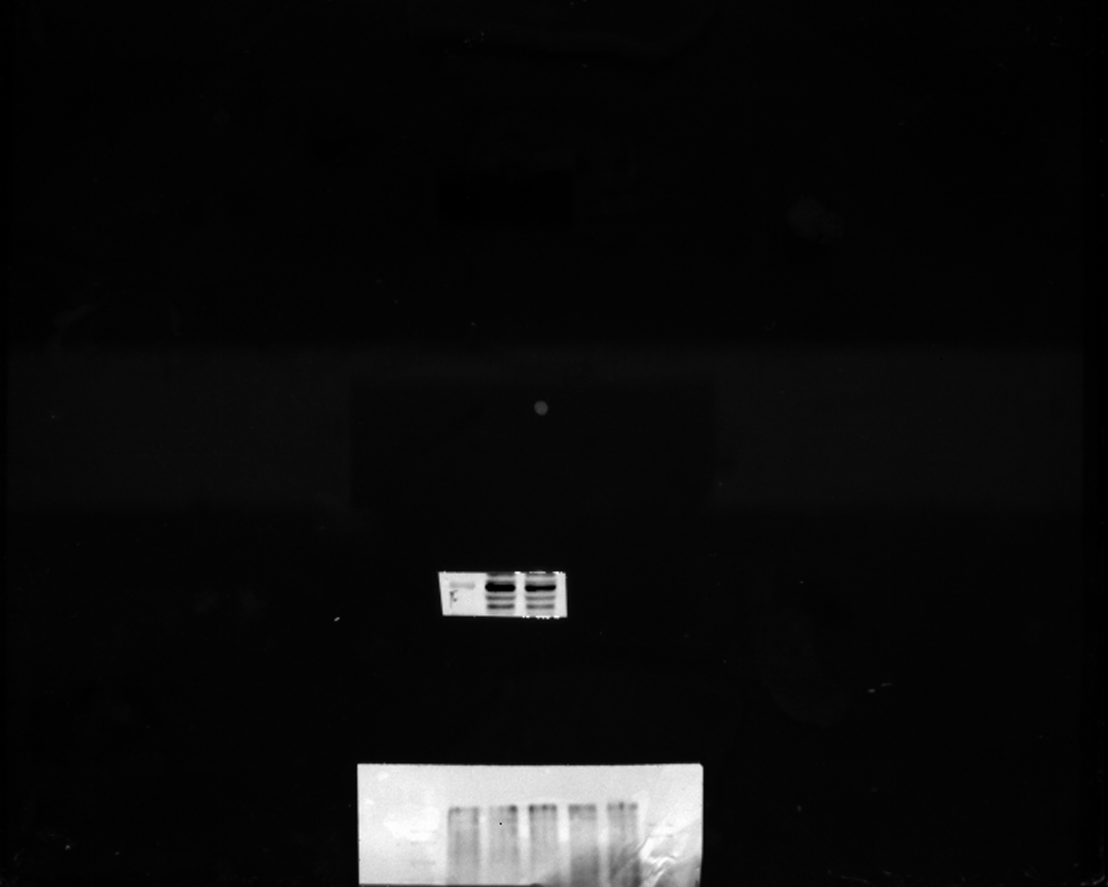


5E IP-UBE4B UBE4B


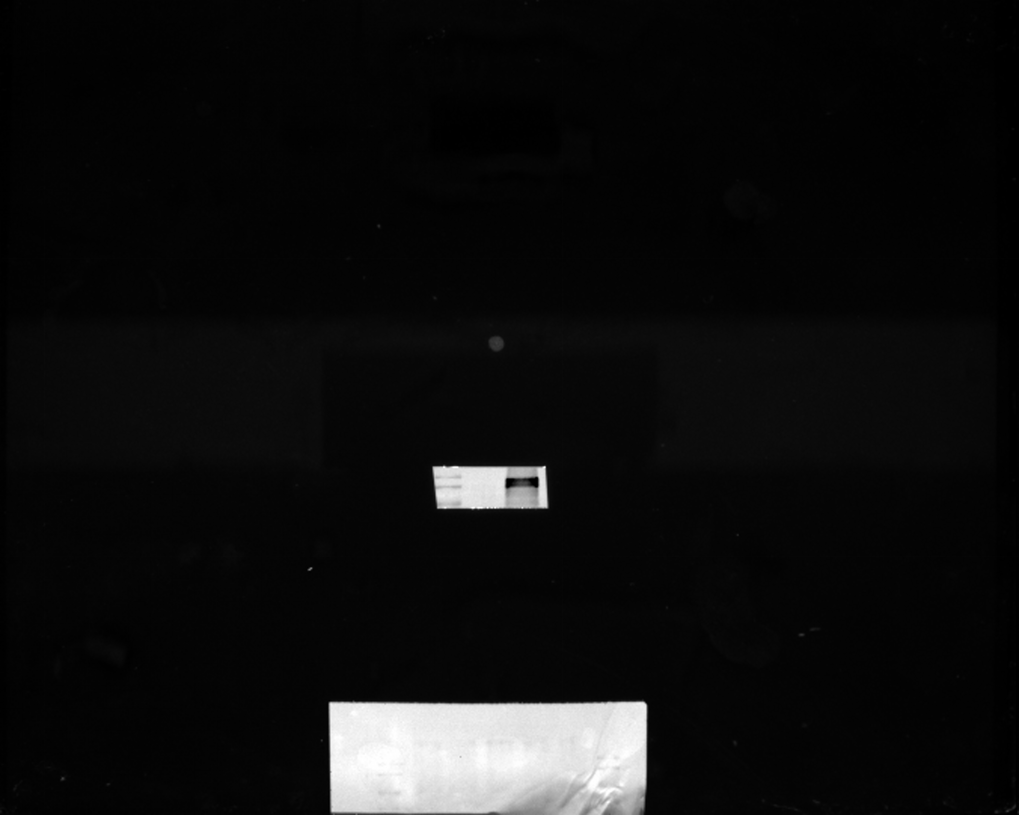


5E IP-UBE4B FAT4


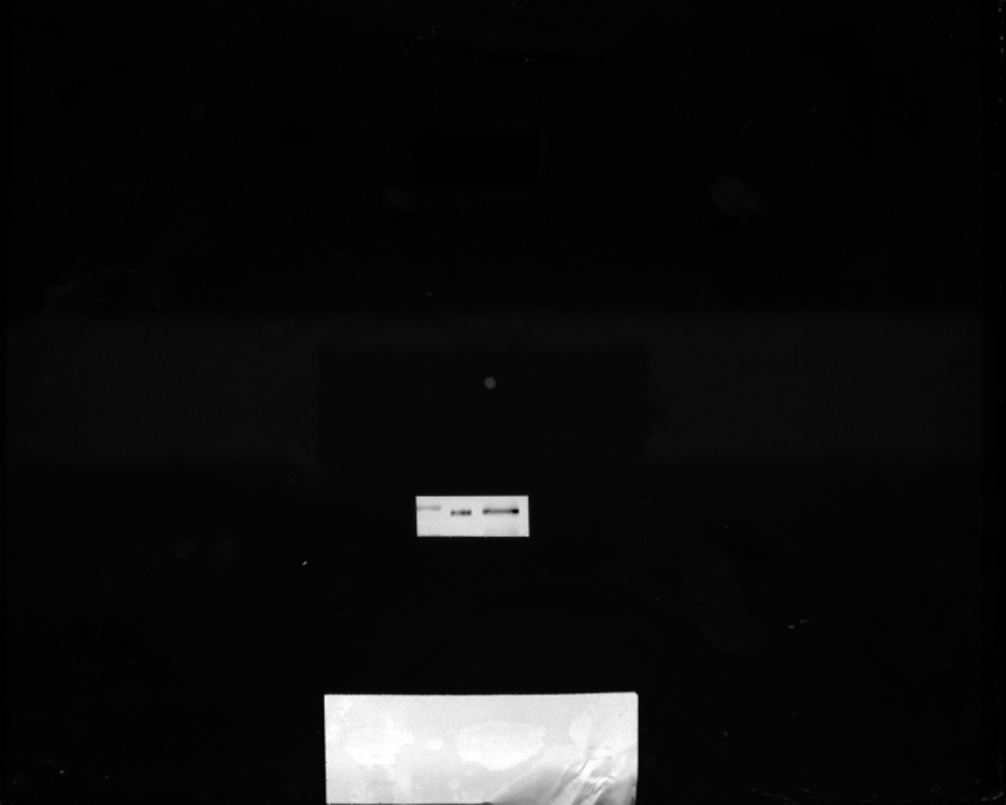


5E IP-FAT4 UBE4B


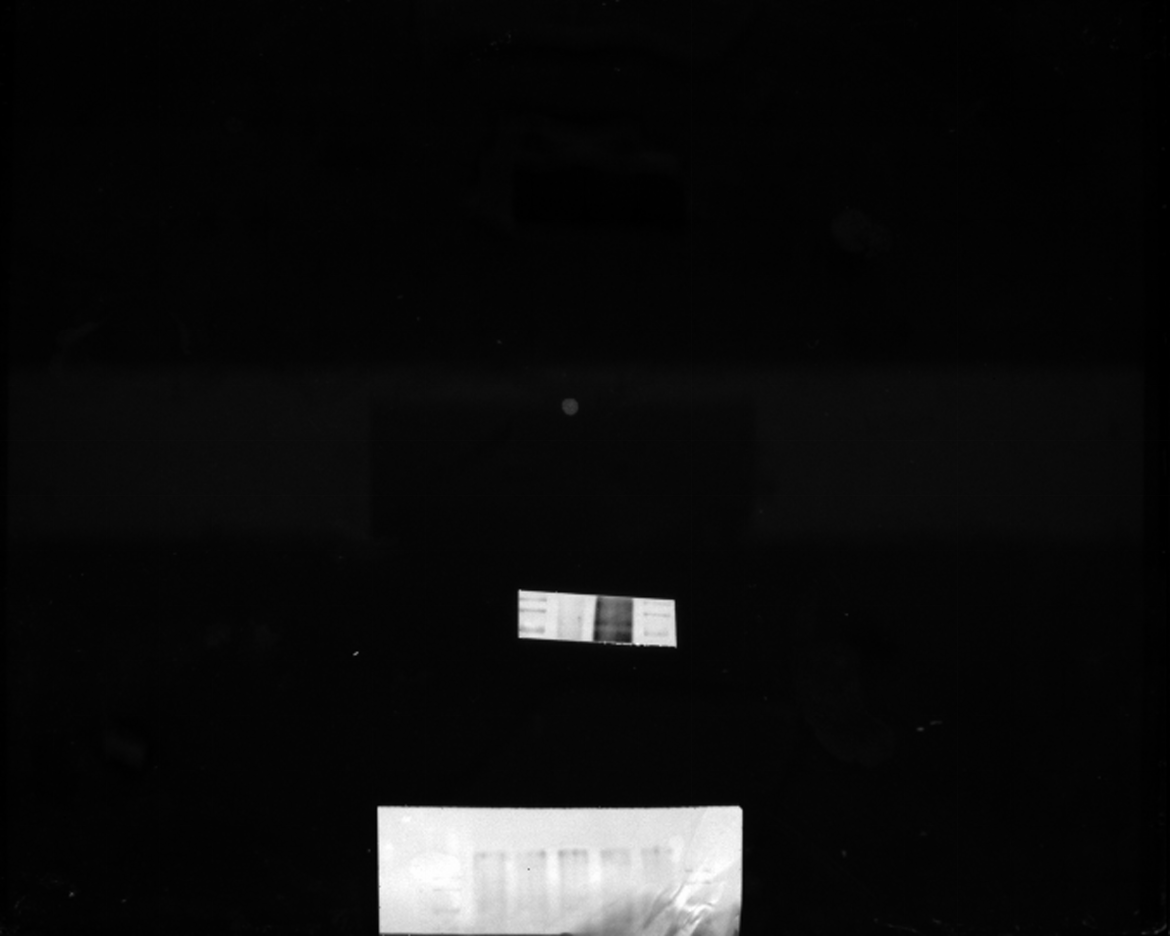


5E IP-FAT4 FAT4


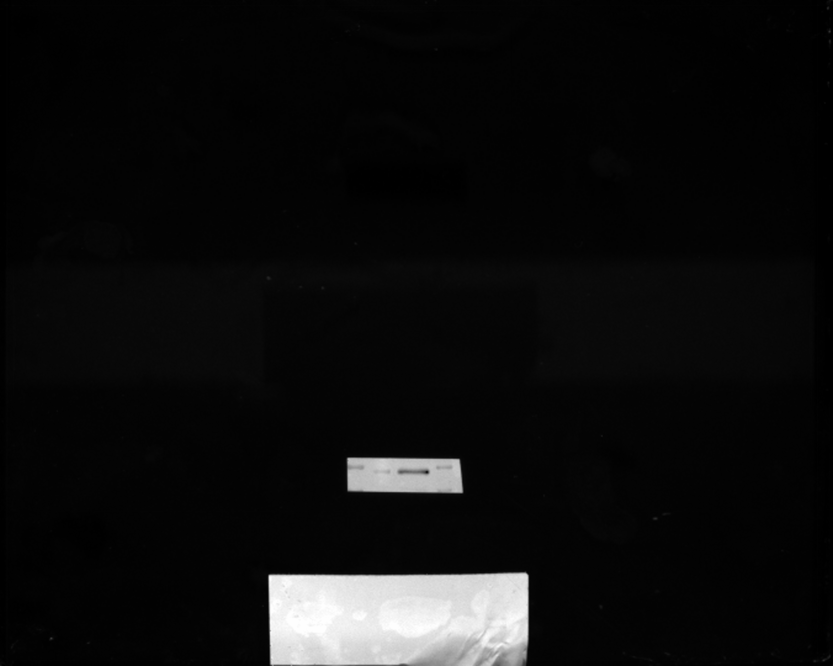


5F Input GAPDH


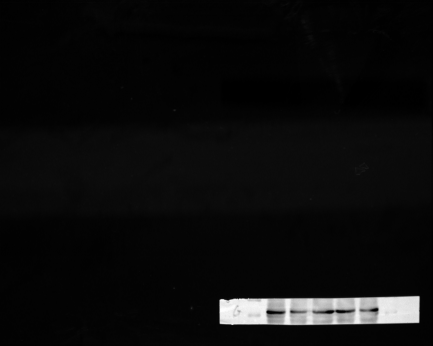


5F Input FAT4


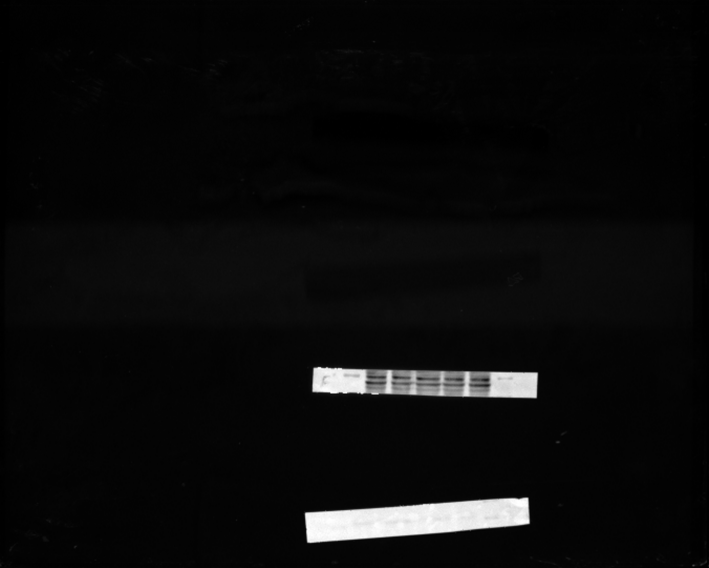


5F Input HA


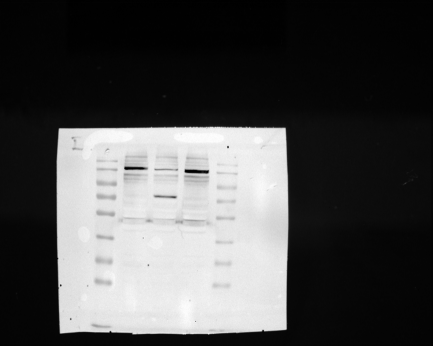


5F IP HA


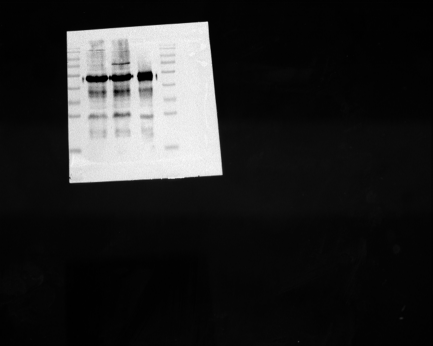


5F IP FAT4


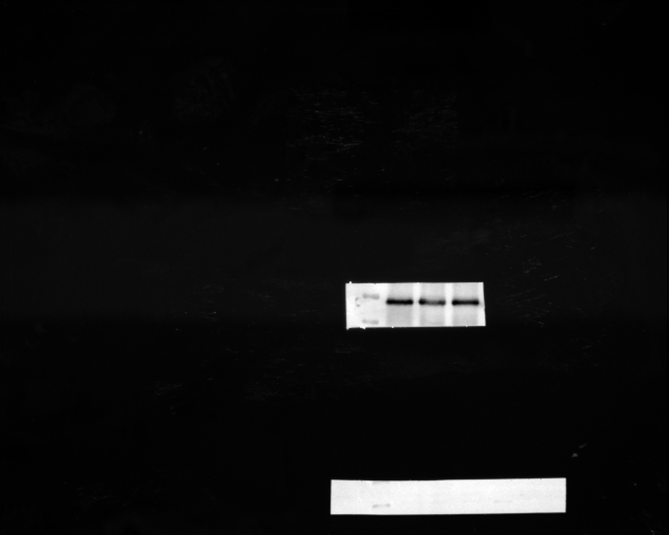


5G input GAPDH


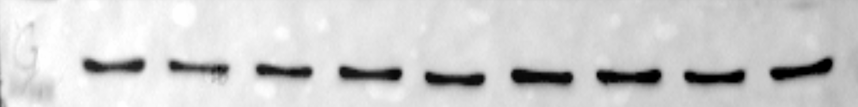


5G input FAT4


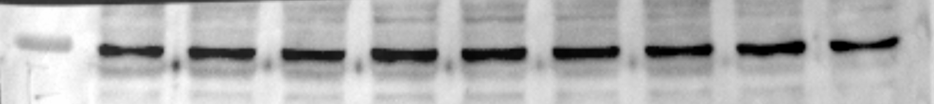


5G input UBE4B


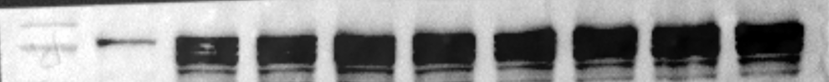


5G IP MYC


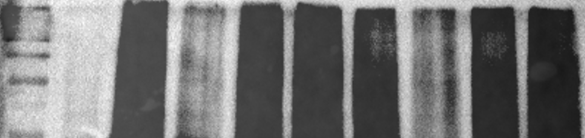


5G IP FAT4


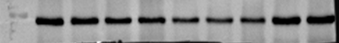


5H input GAPDH


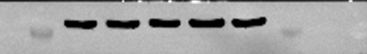


5H input FAT4


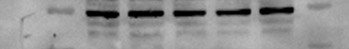


5H input UBE4B


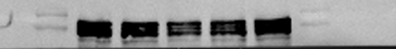


5H IP MYC


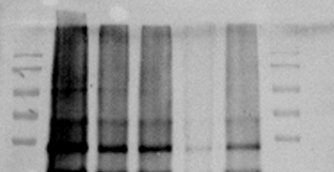


5H IP FAT4


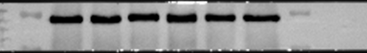

Supplement: Supplementary file 2 — original gel data [file 41419_2025_7794_MOESM2_ESM.docx]
